# Leveraging machine learning with real-world data for hypothesis generation by identifying exposomic predictors in Parkinson's disease among farmers

**DOI:** 10.1177/1877718X261453798

**Published:** 2026-08-01

**Authors:** Pascal Petit, François Berger, Vincent Bonneterre, Nicolas Vuillerme

**Affiliations:** 1 Univ. Grenoble Alpes, CNRS, Grenoble INP, LIG, SANGRIA, Grenoble, France; 2 Univ. Grenoble Alpes, Inserm, Braintech Lab, Grenoble, France; 3 Univ. Grenoble Alpes, CNRS, VetAgro Sup, CHU Grenoble Alpes, Grenoble INP, TIMC, Grenoble, France; 4 CHU Grenoble Alpes, Centre Régional de Pathologies Professionnelles et Environnementales, Grenoble, France; 5 Institut Universitaire de France, Paris, France

**Keywords:** Parkinson's disease, administrative health data, artificial intelligence, explainable machine learning, XGBoost, agriculture, farming exposome, digital public health, environmental epidemiology

## Abstract

**Background:**

Machine learning offers new avenues for complementing traditional epidemiological approaches by analyzing routinely collected, population-based administrative health data.

**Objective:**

This study aimed to identify potential exposomic predictors (hypothesis generation) for Parkinson's disease (PD) across the entire French agricultural workforce.

**Methods:**

We applied XGBoost adapted for Cox proportional hazards modeling to assess approximately 180 exposomic factors derived from nationwide administrative health data within the TRACTOR project. Shapley Additive Explanation (SHAP) values were used to assess the importance of each predictor. To provide both model-based and statistical perspectives, SHAP analysis was complemented with classical Cox regression, allowing for transparent assessment of each predictor's contribution to the model and its statistical association with survival. Sensitivity analyses incorporating different exposure lags were conducted. The study included 424,725 farm managers (6,265 PD cases) and 544,788 farmworkers (2,848 PD cases) aged 50+, analyzed separately due to differences in available variables and coding structures.

**Results:**

Several occupational factors, including duration of involvement in crop farming and viticulture, emerged as key promoting predictors, surpassing age in predictive importance. Beyond conventional predictors such as type 2 diabetes, less conventional predictors were identified, including work diversification, seasonal employment, hypercholesterolemia, epilepsy, antidepressant use, anxiolytic use, and antibiotic use.

**Conclusions:**

These results contribute to a growing body of evidence supporting the integration of occupational health considerations into PD research and highlight the importance of exploring and identifying potential farming-related risk factors in PD development.

**Plain language summary title:**

**Using nationwide French farming data and machine learning to explore potential factors linked to Parkinson's disease. This study investigates how work-related and health-related factors may contribute to Parkinson's disease risk among farm managers and farmworkers**

## Plain language summary

**Why was the study done?:**

While genetics explain only a small fraction of cases, environmental factors, such as those related to farming, may play a major role in Parkinson's disease (PD). Understanding these factors could help identify people at higher risk and guide prevention strategies.

**What did the researchers do?:**

We analyzed administrative health data of over 969,000 French agricultural workers aged 50 and older, including both farm managers and farmworkers. Using a type of artificial intelligence called machine learning, we looked at nearly 180 different factors, including types of farming work, years of experience, medical conditions, and medication use, to investigate which were most strongly associated with PD.

We also used traditional statistical methods to confirm these associations and conducted additional analyses to test the robustness of our findings.

**What did the researchers find?:**

Several work-related factors, such as long involvement in crop farming and in viticulture were linked to a higher risk of PD. Age and some health conditions, including hypercholesterolemia, type 2 diabetes, epilepsy, and use of certain medications like anxiolytics and antibiotics, also appeared associated with PD risk. Conversely, factors such as work diversification, seasonal employment, and total years of farming experience were linked to a lower risk. These associations were observed consistently across farm managers and farmworkers.

**What do the findings mean?:**

The study highlights the importance of considering occupational exposures when studying PD. Certain types of farming work and medical factors may help identify groups at higher risk. While the results do not prove that these factors cause PD, they provide new directions for research, including potential links to environmental exposures. Ultimately, these insights could inform future strategies to prevent or reduce the risk of PD among agricultural populations.

## Background

In recent decades, increasing concern about the health of agricultural workers has elevated this issue to a public health priority. Farming populations exhibit distinct health profiles, particularly a higher prevalence of certain neurodegenerative diseases such as Parkinson's disease (PD).^1–4^

PD is the most common movement disorder globally.^5–8^ It is a progressive neurodegenerative condition that affects millions of individuals worldwide, substantially impairing quality of life.^1,6^ Its etiology is multifactorial, involving a complex interplay between genetic, behavioral, and environmental factors.^9^ Notably, only about 5–10% of PD cases are monogenic, caused by pathogenic variants in single genes.^5,6,8,10–14^ Overall heritability is commonly estimated to be around 30%, while currently identified genetic variants explain approximately 16–36% of this heritable risk.^8,11,15–19^ Aggregate polygenic liability likely interacts with environmental influences to increase disease risk among individuals without monogenic forms.^17–19^ Several of environmental factors, such as smoking and alcohol consumption,^1,5,7,10^ have been identified as potential contributors to PD risk.^16,20–25^ Understanding these associations is critical for informing prevention strategies.^8,16^ However, the precise role of many environmental factors remains unclear, particularly regarding whether they are causative, contributory, or protective.^1,8,26–29^ This ambiguity may stem from several methodological challenges, including the long prodromal phase of PD, which complicates the differentiation between early symptoms and independent risk factors.^30^ Additional obstacles include small sample sizes, limited follow-up durations, and geographical heterogeneity across study populations, as well as the restricted range of risk factors typically examined in individual studies.^1,26,30^ Consequently, certain environmental exposures, especially pesticides, remain central to ongoing scientific and public health debates regarding PD etiology.^8,11,12,26–28^^,31^

A recent review emphasized the urgent need for large-scale cohort studies to advance the understanding of PD risk factors.^10^ Although traditional epidemiological designs remain essential, they are often constrained by limited sample sizes, restricted geographic coverage, and resource-intensive data collection. In this context, administrative health data provide a valuable complementary approach.^3^ These population-based datasets encompass large cohorts over extended time periods and enable retrospective analyses without requiring new data collection efforts.^3^ Recent progress in artificial intelligence, coupled with growing access to real-world data, has further expanded opportunities in public health research, particularly through the application of machine learning to identify risk factors.^3^ Applying artificial intelligence to administrative health data may therefore yield important insights into public health challenges such as identifying potential PD predictors for hypothesis generation. Overall, the integration of artificial intelligence with administrative health data represents a promising avenue for uncovering potential contributors to PD at the population scale.

Investigating PD risk factors specific to agricultural populations remains particularly complex and insufficiently explored.^3^ The diversity of farming practices and associated exposures poses a substantial challenge for identifying consistent, occupation-specific risk factors. Nevertheless, this effort is essential for tailoring preventive strategies and more effectively targeting at-risk subgroups.^3^ Our previous research on the entire French farm manager (FM) workforce revealed major variation in PD risk across different farming activities, suggesting that heterogeneous environmental exposures (i.e., exposome) may play a role and warrant further investigation.^32^ Despite these insights, no study has yet provided a comprehensive assessment of potential PD predictors among farmers using both administrative health data and machine learning approaches.^3^ Building on our earlier work,^32^ the present study aims to advance the understanding of PD risk, particularly for hypothesis generation, in farming populations by applying explainable machine learning techniques to nationwide administrative health data covering the entire French farming workforce.

## Methods

This study adheres to the REporting of studies Conducted using Observational Routinely-collected health Data guidelines (supporting information).

### Data source

In France, farmers are covered by a specific social security system known as the Mutualité Sociale Agricole (MSA).^32^ Selected routinely collected data from the MSA were made accessible for research under the TRACTOR project.^32,33^ The MSA records distinguish between two occupational subgroups: FMs and farmworkers. FMs are individuals who own or operate farms and are actively engaged in farming, whereas farmworkers are employed by FMs to support farming activities, encompassing both permanent and contingent laborers.

MSA collects two primary types of administrative health data: insurance contribution records and insurance claims. Contribution records contain sociodemographic and occupational variables, including farm characteristics and specific farming activities (Tables S1–S2). Insurance claims include diagnoses of long-term chronic diseases, occupational diseases covered under workers’ compensation law, workplace accident reports, and records of reimbursed medications. The insurance contribution data available spanned 2002–2016, while insurance claims covered the period from 2012–2016. Long-term chronic diseases were coded using the International Classification of Diseases, 10^th^ revision, and medications were classified using the Anatomical Therapeutic Chemical system. While the structure of insurance claims was consistent across both subgroups, the contribution data varied substantially in scope, structure, and coding conventions. For instance, farming activities were categorized into 26 distinct classes for FMs (e.g., pig farming) and 22 for farmworkers (e.g., small animal farming), reflecting categorizations defined by national thesauri codified in French legislation (Tables S1–S2).^32^

### Study population and outcome

The cohort comprised all individuals recorded as having worked in agriculture at least once between 2002 and 2016. For PD risk assessment, the analysis was restricted to those aged 50+, in alignment with the typical onset age of PD.^10^ Given differences in available variables, data structures, and coding systems, separate analyses were conducted for FMs and farmworkers.

Building upon previous research within the same population, individuals were identified as PD cases if, after January 1, 2013, they had at least one insurance claim for PD.^32^ In addition, individuals with at least one reimbursement for medications exclusively indicated for PD treatment were also classified as PD cases (Table S3). However, those reimbursed solely for anticholinergics or neuroleptics were excluded from PD case classification.^32^

### Predictors

Predictor selection was informed by an integrative, expert-guided review of the literature.^1,2,4–7^^,10,26–28,31,34,35^ We considered potential true determinants, intermediate factors, disease consequences (e.g., prodromal features or progression-related events), and possible confounders, and included them when available in the MSA data. This strategy was motivated by the substantial difficulty of disentangling the effects of multiple environmental exposures (i.e., exposome) on PD,^8,11,26–28^^,31^ as well as persistent uncertainty regarding the roles of many factors, whether causal, intermediate, or consequential.^1,11,26–28^ We also hypothesized that occupational factors (e.g., type of work) could influence PD onset and progression, particularly given that PD is considered a farming-related disease in France.^36^ Consequently, the final models included 184 (of 6852 initially available) and 183 (of 6838 initially available) independent exposomic variables for FMs and farmworkers, respectively (Table S4). These variables encompassed sociodemographic characteristics (e.g., age, sex), occupational history (e.g., cumulative years of experience), and health-related information (e.g., prior medical diagnoses), and were selected to reflect existing knowledge and our hypotheses. Sex was recorded at birth in the MSA administrative files. Information on gender identity, ethnicity, genetic background, or family history was not available.

Lifestyle-related confounders such as smoking and alcohol consumption, both relevant to PD etiology,^1,5,7,10^ were not captured in MSA data. To partially address this limitation, we imputed categorical variables for smoking (current vs. non-smoker) and alcohol consumption (yes/no) using prevalence estimates derived from the AGRICAN study, the largest French agricultural prospective cohort, which is representative of the agriculture population in France.^37–39^ AGRICAN participants were drawn from the same national source (MSA) as our study population,^37^ and therefore represent a subset of the same underlying workforce. Although our study covered the period 2002–2016 and AGRICAN recruited participants in 2005–2007,^37^ both populations share similar occupational backgrounds. We therefore assumed that lifestyle prevalences estimated in AGRICAN were broadly representative of the underlying population and could serve as reasonable proxies, while acknowledging potential temporal and selection differences. Despite these limitations, AGRICAN estimates provide the most contextually appropriate population-level reference for imputing lifestyle factors in our data. Under this assumption, smoking and alcohol consumption prevalences reported in AGRICAN were used as external proxies to define the distribution of these behaviors in our dataset. Prevalence estimates were applied using a probabilistic (Bernoulli) draw procedure to ensure that group-specific distributions reflected those observed in AGRICAN. Random number generation was seeded to ensure replicability. Specifically, smoking prevalence among PD cases was set at 2.8%.^38^ Among non-cases, prevalences of 3.2%, 10.0%, 6.3%, and 16.0% were applied for female FMs, male FMs, female farmworkers, and male farmworkers, respectively.^37^ Alcohol consumption prevalence among PD cases was set at 69.2%,^38^ with corresponding prevalences of 59.5%, 82.6%, 55.8%, and 90.5% for the same four non-case groups.^37^ As subgroup-specific prevalence data by farming activity were not available in published AGRICAN materials, estimates were applied uniformly within each sex-farming activity-occupational sector stratum, acknowledging the potential for residual confounding. More broadly, this approach assumes that the distribution of these behaviors is reasonably stable across farming activities and over time, and that smoking and alcohol consumption are independent of other covariates. These assumptions may not fully hold, and the imputation does not capture potential variation by farming activity or other sociodemographic factors. Consequently, residual confounding due to misclassification or unmodelled heterogeneity cannot be excluded. Pesticide exposure, another potential key factor in PD etiology,^8,11,12,26–28^^,31^ could not be included because detailed prevalence data stratified by sex, farming activity, or case status were not available in published AGRICAN materials.

The observation window for health-related predictors was limited to 2012–2016. Accordingly, the year 2012 (or the first year of observation for individuals entering farming between 2013–2016) was treated as a run-in period. This run-in period served to limit several sources of bias, including immortal-time bias, differential misclassification, and reverse causation. The formal follow-up period began on January 1, 2013. No MSA data recorded after the start of follow-up were used to compute baseline predictors. Medication exposure was defined as the number of reimbursed prescriptions during the run-in period (i.e., 2012, or the first year of observation for those entering farming between 2013–2016). Disease history (binary: yes/no) was defined by the presence of at least one disease insurance claim in the same period. Baseline sociodemographic and occupational variables were defined at the start of follow-up. For example, cumulative years in a given farming activity were computed from the individual's first recorded farming year (e.g., 2002) to the beginning of follow-up (e.g., 2013). Categorical predictors (e.g., sex) were one-hot encoded to avoid imposing ordinal structure.

To assess potential protopathic bias, we conducted several sensitivity analyses using alternative exposure lag periods, consistent with previous work.^40–43^ In the first sensitivity analysis, we applied a uniform 1-year exposure lag to all predictors. Individuals diagnosed or censored before January 1, 2014, and those entering farming between 2014–2016 with fewer than two years of follow-up, were excluded to ensure that all predictors preceded disease onset by at least one year. In the second sensitivity analysis, health-related predictors were lagged by one year, while sociodemographic and occupational variables were assigned a 5-year exposure lag. For the second sensitivity analysis, we excluded individuals diagnosed or censored before January 1, 2014, as well as those entering farming between 2014–2016 with fewer than two years of follow-up, to maintain a minimum 1-year lag for health-related variables. Additionally, only individuals with at least five years of historical sociodemographic and occupational data before follow-up were retained. Longer exposure lag periods were considered but deemed infeasible because they would require excluding most participants.

In a third sensitivity analysis, we re-estimated models after removing the smoking and alcohol variables, which were based on external estimates. In a fourth sensitivity analysis, we re-estimated the models after removing age and sex. In a fifth sensitivity analysis, we further excluded smoking and alcohol consumption in addition to age and sex. Finally, in a sixth sensitivity analysis, we estimated the baseline model including only age and sex.

### Statistical analysis

All analyses were conducted using R version 4.5.1^®^ on Windows 11. Descriptive statistics were computed by summarizing continuous variables using means and standard deviations or medians and interquartile ranges, as appropriate, while categorical variables were described using absolute frequencies and percentages. Pairwise correlations were also assessed, using Spearman's correlation coefficient for continuous-continuous pairs, point-biserial correlation for continuous-binary pairs, and the phi coefficient for binary-binary pairs.

Separate survival models were developed for FMs and farmworkers using XGBoost with a Cox proportional hazards objective to predict time to PD diagnosis.^44,45^ The outcome was defined as the duration from the start of follow-up to the earliest of either an insurance claim or a PD-specific medication reimbursement for cases, or to the censoring date or end of follow-up for non-cases. Each subject's data comprised a binary event indicator and a corresponding follow-up time.

Model training involved minimizing the negative partial log-likelihood of the Cox proportional hazards model implemented in XGBoost. Hyperparameters were optimized on the training data using random search over a predefined grid with five-fold cross-validation. This cross-validation was used solely for hyperparameter tuning and was not implemented in a nested framework. The final model was then fitted on the entire training set using the selected hyperparameters. Model performance was evaluated using the concordance index (C-index), a standard metric of survival model discrimination ranging from 0 (random prediction) to 1 (perfect prediction).^46^ The dataset was split into 70% training and 30% test sets using stratified k-means-based multi-strata sampling, which jointly stratified individuals by survival time and event status to preserve proportional representation and improve the robustness of model evaluation. To address class imbalance between PD and non-PD cases, inverse-frequency weighting was applied, assigning each observation a weight inversely proportional to the frequency of its event class in the training data.^47^ From a predefined hyperparameter grid, up to 10 parameter combinations were randomly selected and evaluated during the tuning process. The grid included the following candidate values within predefined ranges: learning rate (0.05–0.1), maximum tree depth (3–5), minimum child weight (5–20), number of boosting rounds (50–200), and subsample and column sampling ratios (0.7–1.0). These ranges were chosen to balance predictive performance with computational efficiency. To address potential optimism, final model performance was assessed on the independent 30% holdout test set, which provides an approximately unbiased estimate of predictive performance.

To enhance model interpretability, SHAP values were computed on the test dataset to quantify the marginal contribution of each predictor to model predictions.^48,49^ SHAP values, grounded in Shapley game-theoretic principles, capture both the main effect of a predictor and its interactions with all other features, providing a comprehensive measure of feature contribution. The directionality of each feature's association with predicted risk was inferred through pairwise comparisons between feature values and corresponding SHAP values. For binary features, SHAP distributions of the two groups (e.g., exposed vs. unexposed) were compared, with the proportion of instances in which the higher-value group exhibited a greater SHAP value corresponding to the normalized Mann-Whitney U statistic. For continuous features, values were discretized into 200 bins, and all bin pairs with differing mean feature values were compared to determine concordant (higher-feature bin has higher mean SHAP) and discordant (higher-feature bin has lower mean SHAP) pairs. Features were classified as promoting predictors if ≥55% of comparable pairs were concordant, mitigating predictors if ≥55% were discordant, and neutral/uncertain otherwise. The 55% threshold was chosen as a heuristic to reflect a minimal departure from equality (50%), reducing misclassification when concordant and discordant pairs were nearly balanced. To further explore the relationship between key features and PD, SHAP dependence plots were generated. These plots illustrate how variation in individual features influences the model's output while accounting for interactions with other variables.^50^ They also help identify potential non-linear associations, threshold effects, and interactions.^50^

It is important to note that SHAP values quantify each feature's contribution to the model's predictions, including interactions with other predictors, but do not represent causal effects or epidemiological effect sizes. To provide both model-based and statistical perspectives, SHAP analysis was complemented with classical Cox regression. Mean absolute SHAP values were used to quantify each feature's relative influence in the XGBoost survival model, and SHAP direction indicated whether higher feature values increased (promoting) or decreased (mitigating) predicted risk. For each feature, classical Cox proportional hazards models adjusted for age and sex were fitted using the test dataset to estimate hazard ratios (HRs) with 95% confidence intervals. Features were classified as positively associated, negatively associated, or uncertain if the 95% confidence interval included 1. To control for multiple testing and reduce the risk of false-positive findings, the Benjamini-Hochberg approach was applied. The C-index from each feature-specific model was also reported to assess its discriminative ability. As the C-index reflects ranking performance only and may be biased under high censoring or small hazard differences,^51^ these values should be interpreted as supportive rather than definitive. This combined approach enables direct comparison between machine learning-derived feature importance and classical statistical effect estimates, highlighting predictors that are both influential in the model and statistically associated with survival, while avoiding overinterpretation of either measure alone. In addition, survival plots (Kaplan-Meier curves) stratified by age category and sex were also generated to provide a descriptive visualization of survival patterns across key demographic strata and to provide an interpretable complement to model-based measures.

## Results

### Population characteristics

The final FM cohort included 424,725 individuals aged 50+, of whom 6,265 (2.01%) were identified as incident PD cases. The mean baseline age was 60.7 years (SD = 9.03), and the mean age at diagnosis was 62.0 years (SD = 8.18). Females represented 41.8% of the FM cohort and accounted for 46.0% of PD cases. Additional descriptive statistics are reported in Tables S5-S6. Among FMs, 11,182 (2.63%), 7,944 (1.87%), 6,941 (1.63%), 6,417 (1.51%), and 585 (1.38%) individuals began working in 2012, 2013, 2014, 2015, and 2016, respectively. Age was negatively correlated with median farm surface area (r = −0.26), but positively correlated with years of experience (r = 0.50), working as a solidarity contributor (defined as managing a farm under 12.5 hectares or working fewer than 1200 h annually) (r = 0.50), and years spent in most farming activities, particularly crop farming (r = 0.12), cow farming (r = 0.09), and unspecified or mixed farming (r = 0.10) (Figure S1). Years of experience were strongly positively correlated with time spent in farming activities. Working as a solidarity contributor was negatively correlated with median farm surface area (r = −0.46).

The farmworker population comprised 544,788 individuals aged 50+, among whom 2,848 (0.52%) were diagnosed with incident PD. The mean baseline age was 56.5 years (SD = 4.97), and the mean age at diagnosis was 56.8 years (SD = 4.88). Female farmworkers represented 38.0% of the cohort and 36.0% of PD cases. Further descriptive results are provided in Tables S6–S7. Among farmworkers, 18,006 (3.31%), 22,420 (4.12%), 14,544 (2.67%), 13,807 (1.51%), and 12,901 (1.38%) individuals started working in 2012, 2013, 2014, 2015, and 2016, respectively. Age was negatively correlated with seasonal employment (r = −0.23), but positively correlated with years of experience (r = 0.40), employment in companies with fewer than 20 workers (r = 0.13), and years spent in viticulture (r = 0.05) (Figure S2). Seasonal employment was negatively correlated with years of experience (r = −0.44) and the proportion of part-time work (r = −0.24), but positively correlated with years spent in viticulture (r = 0.28).

In the first sensitivity analysis, the cohort included 367,527 FMs, of whom 5,850 (1.59%) had PD. The corresponding farmworker cohort included 321,385 individuals, with 2,088 (0.65%) PD cases. In the second sensitivity analysis, the cohort comprised 245,334 FMs, of whom 3,618 (1.47%) had PD. The corresponding farmworker subset included 142,350 individuals, with 802 (0.56%) PD cases. For the third, fourth, fifth, and sixth sensitivity analyses, both FM and farmworker cohorts were comparable to those included in the main analysis.

### Model performance

The predictive models demonstrated high and consistent performance across both farming populations. The optimal hyperparameters selected for training are shown in Table S8. Among FMs, the C-index from five-fold cross-validation on the training set was 0.942 [0.940; 0.945], and the model achieved a C-index of 0.936 [0.931; 0.940] on the independent test set (Table 1). Among farmworkers, the corresponding C-indices were 0.917 [0.911; 0.924] (training) and 0.905 [0.892; 0.916] (test), reflecting strong discriminative performance with minimal overfitting. Performance remained robust in sensitivity analyses (Table 1), with C-indices ranging from 0.931 [0.928; 0.933] to 0.941 [0.936; 0.947] (training) and 0.928 [0.923; 0.932] to 0.935 [0.925; 0.944] (test) for FMs, and from 0.882 [0.872; 0.889] to 0.918 [0.913; 0.923] (training) and 0.881 [0.869; 0.891] to 0.905 [0.897; 0.912] (test) for farmworkers. The only exception was the baseline model (sixth sensitivity analysis), which included only age and sex and showed substantially lower performance, with C-indices of 0.671 [0.664; 0.677] (training) and 0.667 [0.656; 0.675] (test) for FMs, and 0.650 [0.637; 0.664] (training) and 0.638 [0.620; 0.656] (test) for farmworkers, highlighting the substantial improvement in predictive performance gained by incorporating additional predictors. The consistently small differences between training and test performance support the generalizability of the models.

**Table 1.** Comparison of model performance across analyses and farming populations.

Analysis	Farm manager		Farmworker	
	C-index train [95% CI]	C-index test [95% CI]	C-index train [95% CI]	C-index test [95% CI]
**Main analysis**	0.942 [0.940; 0.945]	0.936 [0.931; 0.940]	0.917 [0.911; 0.924]	0.905 [0.892; 0.916]
**SA1**: application of an uniform 1-year lag to all predictors	0.941 [0.936; 0.947]	0.935 [0.925; 0.944]	0.918 [0.913; 0.923]	0.905 [0.897; 0.912]
**SA2**: application of a 1-year lag to all health-related predictors and a 5-year lag to all other predictors	0.940 [0.937; 0.943]	0.929 [0.923; 0.937]	0.915 [0.907; 0.924]	0.883 [0.865; 0.898]
**SA3**: not considering smoking and alcohol consumption	0.937 [0.935; 0.939]	0.933 [0.930; 0.938]	0.910 [0.903; 0.917]	0.904 [0.891; 0.914]
**SA4**: not considering age and sex	0.935 [0.932; 0.938]	0.929 [0.924; 0.933]	0.893 [0.886; 0.901]	0.883 [0.867; 0.896]
**SA5**: not considering age, sex, smoking and alcohol consumption	0.931 [0.928; 0.933]	0.928 [0.923; 0.932]	0.882 [0.872; 0.889]	0.881 [0.869; 0.891]
**SA6**: baseline model including only age and sex	0.671 [0.664; 0.677]	0.667 [0.656; 0.675]	0.650 [0.637; 0.664]	0.638 [0.620; 0.656]

Note: 95% CI: 95% confidence interval.

### Most important predictors

**Figure 1.**
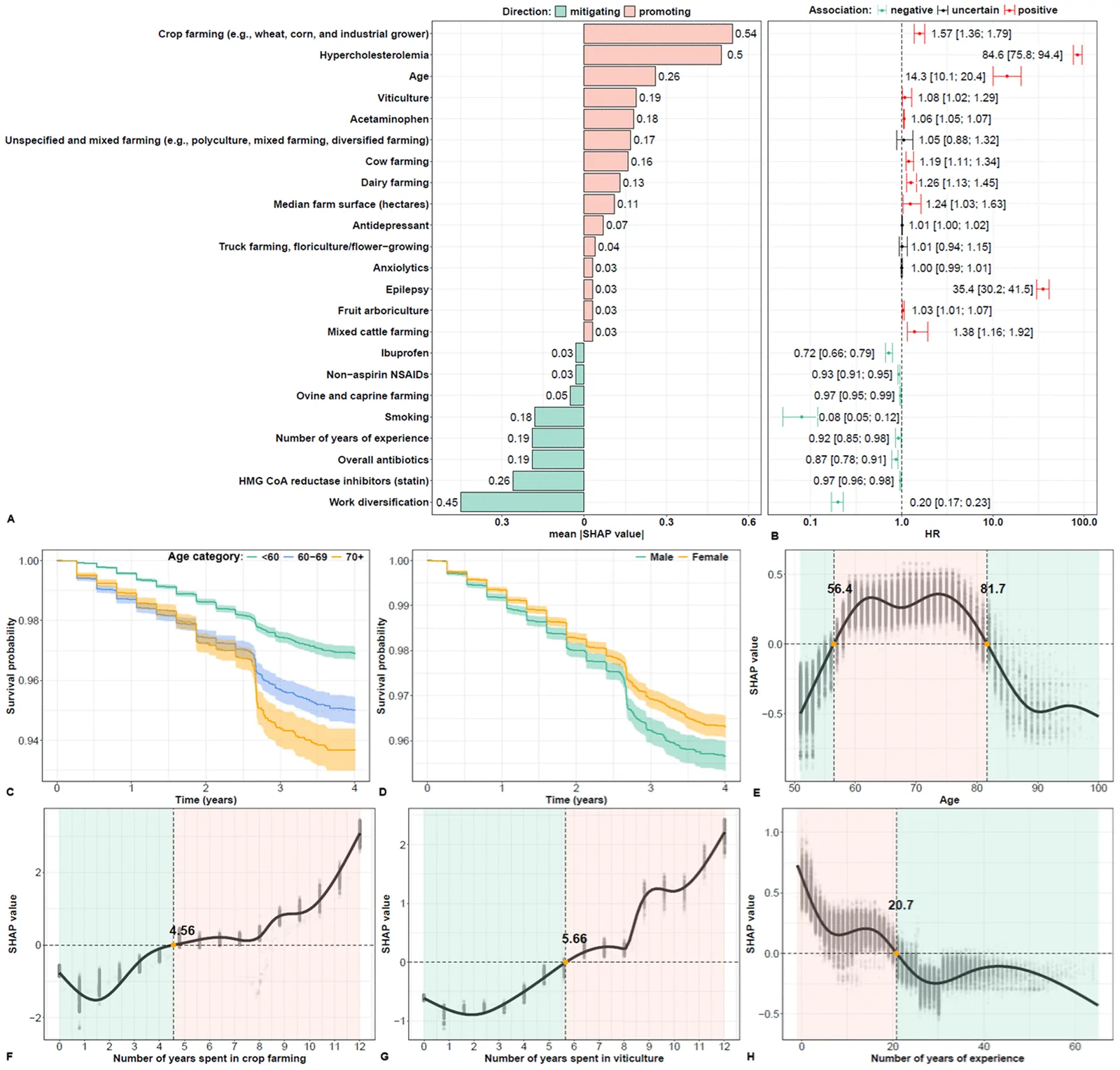
Panel plot of the most important predictors and their impact on PD risk in farm managers (main analysis). ***A***: Summary plot of the most important predictors (mean |SHAP value| ≥ 0.01) ranked by mean absolute SHAP value. The bar direction indicates the direction of association, with features showing positive SHAP contributions represented by bars extending to the right of zero (promoting predictors) and those with negative contributions represented by bars extending to the left of zero (mitigating predictors). ***B***: Error bar plot showing hazard ratios (HRs) and 95% confidence intervals (CIs) for the corresponding key predictors from panel A. With the exception of age and sex, which were mutually adjusted, HRs were estimated using age- and sex-adjusted Cox proportional hazards models. The direction of association is indicated by the position of the error bars, with error bars strictly below 1 indicating negative associations (HR < 1, 95% CI entirely < 1), error bars including 1 indicating uncertain associations (95% CI includes 1), and error bars strictly above 1 indicating positive associations (HR > 1, 95% CI entirely > 1). ***C–D***: Survival plots (Kaplan-Meier curve) stratified by age category and sex, respectively. ***E–H***: SHAP dependence plots for key predictors. Each point represents an individual FM. Background shading indicates regions of positive (SHAP > 0) and negative (SHAP < 0) SHAP values. The fitted line represents a smoothed trend estimated using a generalized additive model (GAM), and the marker indicates where the SHAP value crosses zero.

**Figure 2.**
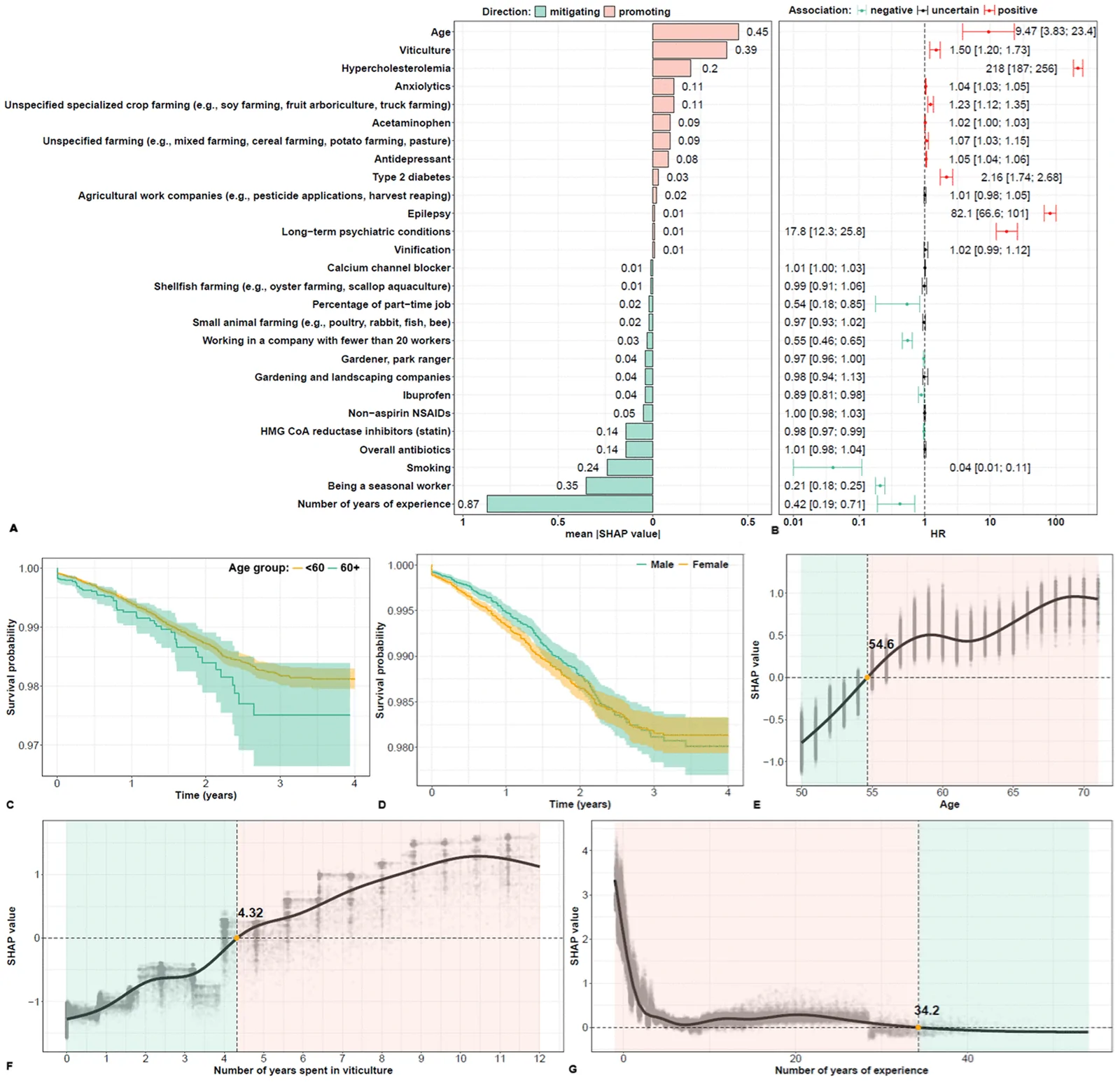
Panel plot of the most important predictors and their impact on PD risk in farmworkers (main analysis). ***A***: Summary plot of the most important predictors (mean |SHAP value| ≥ 0.01) ranked by mean absolute SHAP value. The bar direction indicates the direction of association, with features showing positive SHAP contributions represented by bars extending to the right of zero (promoting predictors) and those with negative contributions represented by bars extending to the left of zero (mitigating predictors). ***B***: Error bar plot showing hazard ratios (HRs) and 95% confidence intervals (CIs) for the corresponding key predictors from panel A. With the exception of age and sex, which were mutually adjusted, HRs were estimated using age- and sex-adjusted Cox proportional hazards models. The direction of association is indicated by the position of the error bars, with error bars strictly below 1 indicating negative associations (HR < 1, 95% CI entirely < 1), error bars including 1 indicating uncertain associations (95% CI includes 1), and error bars strictly above 1 indicating positive associations (HR > 1, 95% CI entirely > 1). ***C–D***: Survival plots (Kaplan-Meier curve) stratified by age category and sex, respectively. ***E–H***: SHAP dependence plots for key predictors. Each point represents an individual farmworker. Background shading indicates regions of positive (SHAP > 0) and negative (SHAP < 0) SHAP values. The fitted line represents a smoothed trend estimated using a generalized additive model (GAM), and the marker indicates where the SHAP value crosses zero.

The most influential predictors of PD risk, based on mean absolute SHAP values, are presented in Figures 1–2, with full results in Table S9.

Among FMs, years spent in crop farming emerged as the strongest promoting predictor, closely followed by hypercholesterolemia, both with mean absolute SHAP values approximately twice that of age (Figure 1). Other influential occupational promoting predictors included years of involvement in viticulture, unspecified and mixed farming, cow farming, dairy farming, and median farm surface area. Conventional health-related promoting predictors included acetaminophen use and type 2 diabetes, while less conventional promoting factors included epilepsy and the use of antidepressants and anxiolytics. Work diversification was the strongest mitigating predictor, with a mean absolute SHAP value nearly twice that of age. Other mitigating occupational predictors included total farming experience and involvement in ovine and caprine farming. Health-related mitigating predictors included smoking and the use of non-aspirin NSAIDs, ibuprofen, and statins, as well as antibiotics and sulfonamides. These patterns were broadly consistent with results from age- and sex-adjusted classical Cox models, although HRs for age were more than tenfold higher (HR = 14.3 [10.1;20.4]) than those of the strongest promoting predictors. The only exceptions were hypercholesterolemia and epilepsy. Smoking (HR = 0.08 [0.05; 0.12]) and work diversification (HR = 0.20 [0.17; 0.23]) showed the lowest HRs among mitigating predictors. In addition, other negative associations emerged in classical Cox regression, in particular for macrolides and lincosamides antibiotics (HR = 0.66 [0.58; 0.74]), sulfonamides and trimethoprim antibiotics (HR = 0.77 [0.69; 0.87]), and working as a solidarity contributor (HR = 0.64 [0.57; 0.70]). To further explore the relationship between key features (i.e., age, crop farming) and PD, SHAP dependence plots were generated (Figure 1). For age, SHAP values showed a marked increase around 56.4 years, indicating an elevated contribution to PD risk beyond this point, followed by a plateau from approximately 62 to 75 years, during which contributions remained positive, and then a decrease with SHAP values becoming negative again after about 81.7 years. Survival plots stratified by age category (<60, 60–69, ≥70) showed lower survival probabilities for the oldest age group, with the lowest survival in those aged ≥70, followed by 60–69, and <60. For years spent in crop farming, SHAP values showed a marked increase around 4.56 years, indicating an elevated contribution to PD risk beyond this point. Years spent in viticulture, exhibited an increasing relationship with SHAP values, with a notable inflection around 5.66 years. Total farming experience exhibited a decreasing relationship with SHAP values, with a notable inflection around 20.7 years.

For farmworkers, age was the most influential promoting predictor, closely followed by years of involvement in viticulture (Figure 2). Additional promoting predictors included acetaminophen use and type 2 diabetes. Less conventional promoting predictors included hypercholesterolemia, engagement in unspecified farming (e.g., mixed, cereal, potato, and pasture farming) and unspecified specialized crop farming (e.g., soy, fruit arboriculture, and truck farming), as well as epilepsy and the use of antidepressants and anxiolytics. The strongest mitigating predictor was total farming experience, with a mean absolute SHAP value almost twice that of age. Other mitigating predictors included seasonal employment, smoking, antibiotic use, proportion of part-time work, non-aspirin NSAID use, ibuprofen use, and calcium channel blocker use. Age- and sex-adjusted Cox models fitted separately for each feature showed consistent directions of association, with HRs for age nearly tenfold higher (HR = 9.47 [3.83; 23.4]) than those of the strongest promoting predictors, except for hypercholesterolemia, epilepsy, bipolar disorders, and long-term psychiatric conditions. Being male had a small positive contribution, which was also apparent in survival plots. For age, the dependence plot revealed an increasing relationship with SHAP values, with contributions becoming positive after about 54.6 years and increasing until plateauing around 67.5 years (Figure 2). For years spent in viticulture, SHAP values showed a marked increase around 4.32 years, indicating an elevated contribution to PD risk beyond this point. Total farming experience exhibited a decreasing relationship with SHAP values, with contributions becoming negative around 34.2 years.

Sensitivity analyses produced results largely consistent with the main analysis (Tables S10-S16; Figures S3-S16). In the first sensitivity analysis, among FMs, only hypercholesterolemia (promoting), crop farming (promoting), statin use (mitigating) and work diversification (mitigating) had mean absolute SHAP values exceeding that of age (Figure S3, Table S10). Overall antibiotic use (mitigating) and years spent in viticulture (promoting) had mean absolute SHAP values similar to that of age. Among farmworkers, viticulture (promoting), total farming experience (mitigating), and seasonal employment (mitigating) were the only predictors with SHAP values exceeding that of age, while hypercholesterolemia (promoting) had a value similar to that of age (Table S10, Figure S5). For both populations, age- and sex-adjusted Cox regression models fitted separately for each feature showed directions of association consistent with the main analysis. Notably, age remained the feature with the highest HR, except for hypercholesterolemia and epilepsy (in both populations), bipolar disorders (farmworkers only), and long-term psychiatric conditions (farmworkers only).

In the second sensitivity analysis (Table S11), several predictors (similar to those in other analyses, with some additions such as overall antibiotic use (mitigating) for FMs and anxiolytics use (promoting) for farmworkers) had mean absolute SHAP values exceeding that of age in both population (Figures S5-S6). However, age remained the feature with the highest HR, except for hypercholesterolemia and epilepsy in FMs, as these features were not selected in the final model for farmworkers. Notably, in FMs, antibiotic use (HR = 0.87 [0.84; 0.90]) and work diversification (HR = 0.20 [0.17; 0.24]) showed low HRs, as did seasonal employment in farmworkers (HR = 0.24 [0.17; 0.33]).

In the third sensitivity analysis, hypercholesterolemia (promoting), crop farming (promoting), statin use (mitigating) and work diversification (mitigating) had mean absolute SHAP values exceeding that of age for FMs, while only viticulture (promoting) and total farming experience (mitigating) exceeded age for farmworkers (Table S12, Figures S7-S8). For both populations, age remained the feature with the highest HR, except for hypercholesterolemia and epilepsy (both populations) and bipolar disorders (farmworkers only). Work diversification (HR = 0.18 [0.16; 0.21]) and macrolides and lincosamides antibiotics (HR = 0.72 [0.65; 0.81]) continued to show some of the lowest HRs among mitigating predictors in FMs.

In the fourth (Table S13) and fifth sensitivity analyses (Table S14), which did not include age and sex, crop farming and hypercholesterolemia remained the strongest promoting predictors for FMs, while work diversification, total farming experience, statin use, and overall antibiotic use remained the strongest mitigating predictors (Figures S9 and S11). Among farmworkers, viticulture and hypercholesterolemia remained the strongest promoting predictors, while total farming experience, seasonal work, and overall antibiotic use remained the strongest mitigating predictors (Figures S10 and S12).

In the sixth sensitivity analysis, the baseline model including only age and sex, age had the highest mean absolute SHAP value, with a negligible contribution of sex among farmworkers (Table S15, Figures S13-S14).

## Discussion

This exploratory study aimed to generate hypotheses by identifying potential predictors of PD in farmers using nationwide, population-based administrative health data and an interpretable machine learning framework. Several occupational variables emerged as top-ranking predictors, in some cases exceeding the predictive importance of age, although age consistently showed the highest HRs among the top promoting predictors, with a few exceptions, in particular for hypercholesterolemia. Importantly, these findings do not imply causality but instead reflect associations intended to support hypothesis generation and to motivate further investigation. SHAP values quantify each predictor's contribution to the model's predictions and do not represent causal effects or epidemiological effect sizes. Accordingly, while this approach is well suited for identifying potential promoting or mitigating predictors, subsequent analyses (e.g., regression-based, pharmaco-epidemiological, or epidemiological studies) are required to validate any true underlying associations.

### Possible promoting predictors

A central finding of this study is that routinely collected exposomic data, encompassing sociodemographic characteristics, occupational records, diagnostic codes, and medication reimbursements, can provide substantial predictive value for PD risk, as evidenced by the performance gain between the baseline model including only age and sex and models incorporating a broader set of predictors. This study comprehensively integrates all these data types into a unified, knowledge-driven machine learning model for PD prediction, offering a holistic view of predictor importance.

Age was confirmed as a strong promoting predictor, consistent with established literature.^6,10^ This aligns with the central role of ageing in PD pathogenesis, including cumulative mitochondrial dysfunction, impaired proteostasis, neuroinflammation, and reduced resilience of dopaminergic neurons.^21,35,52,53^ In both populations, SHAP values for age showed a marked increase around 55–56 years, indicating an elevated contribution to PD risk beyond this point, followed by a plateau during which contributions remained positive. Similar plateau patterns have been reported in other PD studies.^50^ However, among FMs, the contribution of age saturated after 75 years and then reversed, becoming negative after about 81.7 years, which is consistent with existing literature.^35^ This bell-shaped or inverted U-shaped pattern may reflect a ceiling in age-related vulnerability within our 4-year study window or the increasing dominance of other predictors at advanced ages. It may also reflect data-driven effects, as relatively few incident PD cases occurred after this age, with only 26,249 FMs aged ≥80 years, including 265 (1.0%) PD cases, and 45,079 FMs aged ≥75 years, including 642 (1.4%) PD cases. Competing risks and the selection of relatively healthy survivors may also reduce the observed incremental PD hazard at very advanced ages.^35^ Modeling artifacts (e.g., regularization, shallow trees, or monotonicity constraints) may similarly contribute to this pattern, as gradient boosted trees approximate relationships through discrete splits rather than smooth functions. Accordingly, negative SHAP values at the oldest ages in FMs should be interpreted as reflecting lower predicted study-period PD risk relative to the model baseline, rather than a true biological decrease in lifetime susceptibility. Overall, the observed plateau and, in FMs, subsequent decrease, may indicate a saturation effect whereby further age progression contribute minimally to additional risk and other interacting factors become increasingly influential.

Several occupational factors, particularly work diversification, duration of involvement in crop farming, viticulture, and years of farming experience, had mean absolute SHAP values exceeding those of age. This indicates that, within the model, these features contributed more to differentiating cases from non-cases than age. SHAP dependence plots identified a tipping point approximately 4–6 years for involvement in crop farming and viticulture, beyond which their contribution to PD risk became positive. These findings suggest that longer involvement in crop farming and viticulture is associated with increased PD risk. However, despite their higher SHAP contributions, age consistently exhibited HRs more than tenfold greater than those of these predictors, underscoring its dominant epidemiological effect. These differences likely reflect heterogeneity in the farming exposome,^3^ particularly with regard to pesticide use, application methods, and exposure duration.^38,54,55^ Both farming activities are characterized by intensive used of pesticides,^56^ which have been implicated in PD through mechanisms such as microglial activation, oxidative stress, mitochondrial dysfunction, and disruption of dopaminergic signaling.^8,11,12,20,21,26–28^^,31,57,58^ While our data do not capture specific agents, doses, or time-varying exposure patterns, the observed associations support a role for long-term, pesticide-related occupational exposures in PD risk and motivate more detailed exposure assessment in future work.

In contrast, total years of farming experience emerged as the strongest mitigating predictor, potentially reflecting reduced exposure through improved adherence to protective practices over time,^59^ or a shift toward more administrative roles with seniority.^60^ The latter interpretation may be partially supported by dependence plots that identified tipping points around 20 years for FMs and 34–42 years for farmworkers, beyond which the contribution of total farming experience to PD risk became negative. However, it may also partly reflect a “healthy worker survivor” effect, whereby workers who remain in employment for longer are, on average, healthier. Work diversification, working as a solidarity contributor (i.e., managing a small-scale farm or working part-time), and seasonal employment appeared as mitigating predictors, possibly due to reduced cumulative exposure to environmental hazards such as neurotoxic agents.^61^

Although the aforementioned hypotheses about occupational-related factors are plausible, they warrant further investigation. Overall, the strong contribution of occupational variables to PD risk prediction highlights the need for new studies evaluating the potential role of work-related factors as risk or protective determinants, and suggests that incorporating detailed occupational data may be valuable for future epidemiological and public health research on PD.

We also observed consistent findings with several well-established risk and protective factors, such as smoking,,^1,5,7,10,62^ bipolar disorder,^63,64^ depression,^1,7^ type 2 diabetes,^1,34^ and use of statins.^2,7,63,65^ Several less conventional predictors also emerged, including the use of antidepressants,^66,67^ anxiolytics,^66,67^ as well as diagnoses of epilepsy,^15,68,69^ and hypercholesterolemia.^62,70–73^ Epilepsy and hypercholesterolemia consistently showed HR exceeding that of age in both populations. Whether these associations are driven by the underlying disease (e.g., epilepsy), direct pharmacological effects due to chronic use of associated drugs (e.g., antiepileptic drugs), increased comorbidity, residual confounding, or a combination of these factors remains to be determined. The nature of these associations is uncertain, as it is unclear whether these factors represent early manifestations of PD (i.e., prodromal features or treatment of prodromal symptoms of PD) or independent risk factors.^30,31^ For example, the findings related to neuropsychiatric predictors are compatible with the growing evidence that mood and anxiety disorders can precede motor symptom onset and represent prodromal manifestations of PD.^1,7,27,63,64,66,67,74–78^ Proposed mechanisms include early involvement of brainstem and limbic structures, altered monoaminergic neurotransmission, and neuroinflammatory processes.^1,7,27,63,64,66,67,74–78^ In this context, it is difficult to disentangle the contribution of the underlying neuropsychiatric condition from the pharmacological effects of its treatment, and both disease-related and drug-related pathways may be involved. Regarding the association between epilepsy and PD, the mechanisms remain uncertain, but potential explanations include shared vulnerability of cortical-basal ganglia circuits, chronic excitotoxicity, and overlapping genetic or metabolic susceptibilities.^15,68,69^ Regarding hypercholesterolemia, which was among the most influential promoting predictors, existing evidence remains inconsistent, with reports of increased, null, or even decreased risk.^62,70–73^ Mechanistically, elevated cholesterol can impair lysosomal function and promote α-synuclein aggregation, providing a biologically plausible pathway for increased PD susceptibility.^72,79^ At the same time, individuals with hypercholesterolemia often have multiple vascular comorbidities and receive intensive pharmacological treatment, complicating causal interpretation.^62,70–73^ Interestingly, statins, the treatment of choice for hypercholesterolemia, consistently emerged as mitigating predictors in our models. Statins have been hypothesized to exert neuroprotective effects through anti-inflammatory, antioxidant, and vascular mechanisms, but the observational literature is heterogeneous and subject to indication and survival biases.^2,5,7,27,63,80^ In our setting, the opposing directions for hypercholesterolemia and statin use likely reflect a mixture of underlying biological pathways, treatment effects, and complex confounding structures rather than straightforward cause-effect relationships. The design of this study does not allow for precise attribution of the role of these less conventional predictors, underscoring the need for further research to clarify their epidemiological impact and potential clinical relevance.

One notable predictor identified in this study was antibiotic use, for which evidence remains limited. A recent study reported a negative association between PD and lincosamides.^81^ Antibiotic use may be linked to alterations in the gut microbiome, which aligns with growing evidence suggesting that gut dysbiosis may contribute to PD pathogenesis.^26,82–86^ Environmental exposures common in farming, such as fungicides or contact with livestock, may alter both nasal and intestinal microbiota, potentially leading to neurotoxicity through mechanisms including mitochondrial dysfunction and lysosomal disruption.^5,56,86^ Prior research has shown that interactions with livestock can influence the human microbiota,^87–89^ with variations depending on the specific animals and tasks involved.^90,91^ Our finding that PD risk varied across types of animal-farming activities, particularly with higher SHAP values observed in cattle farming, warrants further investigation into potential microbiome-mediated mechanisms.

### Strengths and limitations of this work

This study employed XGBoost, a high-performance machine learning algorithm capable of modeling complex, non-linear relationships and high-dimensional feature interactions while effectively handling collinearity.^44,45,50,92–95^ XGBoost inherently models interaction effects among predictors by allowing tree splits across combinations of features, which is particularly relevant for multifactorial conditions such as PD onset and progression.^50^ We used the XGBoost implementation of the Cox proportional hazards model and extended the workflow to incorporate sample weighting, enabling the algorithm to better address class imbalance within the survival analysis framework. To our knowledge, this framework has not previously been applied to investigate PD predictors. To further enhance interpretability, we incorporated SHAP values and Cox estimates, allowing for transparent assessment of each predictor's contribution to the model and its statistical association with survival.^48,49^

A strength of this large-scale study is the integration of occupational, sociodemographic, diagnostic, and medication data from administrative health data to build explainable survival machine learning models for PD. Several identified predictors are supported by previous literature, reinforcing the feasibility and relevance of our proposed approach. Unlike studies that rely exclusively on diagnostic codes, we combined diagnosis with medication reimbursements to improve case specificity.^96^ Our use of a literature-informed variable selection strategy further mitigated overfitting, enhanced computational efficiency, and reduced sparsity, key challenges in high-dimensional data modeling.^96^

Nonetheless, several limitations should be acknowledged. Feature importance estimated from SHAP values does not imply causality or indicate an epidemiologic effect. The accuracy of administrative coding for health-related variables can vary due to billing practices or reporting bias,^3,93,96,97^ which may attenuate true associations. Moreover, alternative case definitions could influence the results. However, in a previous study conducted in the FM population, we tested multiple case definitions using Cox proportional hazards models and obtained consistent findings.^32^ Another limitation relates to drug-related data. Drug indications were not recorded by MSA and, as was done in other studies, drug exposure was inferred from reimbursement records, assuming that the reimbursed medications were subsequently used.^81^

The MSA data lacked information on key confounders such as genetics, education, ethnicity, and detailed environmental exposures (e.g., pesticide type or dose). The inability to account for genetic susceptibility, which may interact with several predictors, is a limitation. Although occupational categories may serve as indirect proxies,^3^ integrating omics, clinical, genetic, and biomarker data would substantially strengthen future analyses.^92,93,98^ Smoking and alcohol consumption were not directly available and were approximated using prevalence estimates from the AGRICAN cohort.^37,38^ While this approach provided contextually relevant estimates and yielded results consistent with existing literature,^1,5,7,10^ it relies on strong assumptions regarding population comparability. Although AGRICAN and our study population both derive from the MSA and overlap temporally, differences remain. Our study spans 2002–2016, whereas AGRICAN baseline data were collected in 2005–2007. Temporal changes in lifestyle behaviors, such as declining smoking prevalence in France between 2014 and 2019,^99^ may limit the transportability of these estimates. If similar trends occurred within the agricultural population, true smoking prevalence during later years of follow-up may be lower than that assumed in our imputation procedure. However, because the same prevalence parameters were applied uniformly within case and non-case groups, this temporal mismatch would primarily affect the absolute level of exposure rather than the direction of confounding. In addition, AGRICAN relied on voluntary postal recruitment, which may introduce selection bias and healthy volunteer bias, and affect the representativeness of lifestyle behaviors. Such differences may influence the reported prevalence of lifestyle behaviors and may limit the direct applicability and generalizability of AGRICAN-derived prevalence estimates to our full population (i.e., the entire agricultural workforce). Smoking was imputed as a binary indicator of current smoking versus non-smoking, which may lead to exposure misclassification. We chose not to introduce a separate former-smoker category because no information on smoking duration or time since cessation was available. We adopted this simplified categorization because AGRICAN prevalence estimates stratified by detailed smoking history (e.g., duration or pack-years) were not available in published materials and because modeling multiple smoking categories without information on smoking intensity or duration would introduce additional uncertainty. The imputation procedure also assumed independence between lifestyle factors and other covariates. The imputed lifestyle variables were introduced to approximate potential confounding by smoking and alcohol consumption in the absence of individual-level measurements and should therefore be interpreted as probabilistic proxies rather than observed exposures. Future studies could improve upon this approach by using more advanced missing-data methods that leverage external prediction models. For example, recently proposed approaches allow univariate imputation of missing variables using externally estimated regression coefficients and their covariance structure, enabling the transfer of predictive information between datasets without sharing individual-level data.^100^ Applying such methods would require external cohorts such as AGRICAN to publish or share prediction models. Another promising avenue for future research would be collaboration with AGRICAN to access individual-level lifestyle data or to construct a job exposure matrix incorporating information on specific pesticides, demographics and lifestyle characteristics by sex, farming activity, and health status.^101,102^ Alternatively, MSA could consider systematically collecting lifestyle and occupational exposure data, which is not currently practiced.

Another limitation is the restricted temporal coverage of medical data (2012–2016). Although several strategies and sensitivity analyses were implemented to mitigate protopathic, differential, and immortal time biases, these approaches may not fully capture the prodromal period of PD.^30,31^ Reverse causation remains a concern, as prodromal symptoms may influence healthcare utilization, diagnoses, and medication use prior to PD onset. Longer exposure lags could improve specificity but would reduce sample size, potentially compromising statistical power and potentially introducing selection bias. Moreover, the optimal exposure window and lag period for PD risk modeling remain uncertain.^41–43^^,81^ Our choices therefore reflect a necessary balance between internal validity and data availability, underscoring the need for future studies with extended historical data to validate and refine these temporal assumptions.

Lastly, model development and validation were conducted exclusively in a French agricultural population. In the absence of comparable external datasets, generalizability to other populations remains uncertain. Farming practices, regulatory frameworks, and environmental exposures vary internationally, and replication in independent cohorts with detailed exposure and occupational histories is essential. However, while replication in a truly external dataset would be valuable, identifying datasets with comparable variables and detailed occupational and exposure information is highly challenging. In addition, competing risks, such as death, could not be accounted for due to data constraints.

### Potential implications

This study primarily enabled hypothesis generation and the identification of less conventional predictors of PD that warrant further investigation. The observed associations may reflect overall morbidity, reverse causation related to prodromal PD, direct effects of the exposures, residual confounding, or combinations of these mechanisms. Disentangling these processes is not possible within the current design and will require targeted studies tailored to each signal. Observed associations between antibiotic use, hypercholesterolemia, epilepsy, specific occupational exposures (e.g., crop farming, viticulture, cattle farming), and PD risk highlight areas for targeted mechanistic, pharmacoepidemiological, and epidemiological research. Although these findings are not immediately translatable to clinical decision-making, they provide a foundation for the development of occupational surveillance tools and early risk stratification strategies. Identifying modifiable and underappreciated predictors also contributes to broader public health objectives, including PD prevention and the enhancement of occupational health monitoring.

## Conclusions

In summary, this study identified key predictors of PD and developed interpretable, high-performance machine learning survival models using nationwide administrative health data. Our results indicate that occupational exposures, alongside conventional predictors, may influence PD risk in agricultural populations. By integrating explainable machine learning with large-scale real-world data, we provide a complementary approach to traditional epidemiological methods. Future research should aim to validate these findings across diverse populations and further explore emerging associations, particularly those related to antibiotic use and occupational exposures.

## Supplemental Material

sj-pdf-1-pkn-10.1177_1877718X261453798 - Supplemental material for Leveraging machine learning with real-world data for hypothesis generation by identifying exposomic predictors in Parkinson's disease among farmers Supplemental material, sj-pdf-1-pkn-10.1177_1877718X261453798 for Leveraging machine learning with real-world data for hypothesis generation by identifying exposomic predictors in Parkinson's disease among farmers by Pascal Petit, François Berger, Vincent Bonneterre and Nicolas Vuillerme in Journal of Parkinson's Disease
