# Supplementary material for: Leveraging machine learning with real-world data for hypothesis generation by identifying exposomic predictors in Parkinson's disease among farmers
Source: J Parkinsons Dis. 2026 Aug 1;16(6):1174–90. doi: 10.1177/1877718X261453798 (PMC13442685; doi:10.1177/1877718X261453798)
Supplement: Supplementary file 1 [file sj-pdf-1-pkn-10.1177_1877718X261453798.pdf]

Supporting information

Title
Leveraging machine learning with real-world data for hypothesis generation by identifying exposomic predictors in Parkinson's disease among farmers

Authors
Pascal Petit, François Berger, Vincent Bonneterre, Nicolas Vuillerme

Table of contents

Figure S1: Correlation matrix of the main predictors for farm managers 3

Figure S2: Correlation matrix of the main predictors for farmworkers 4

Figure S3: Panel plot of the most important predictors and their impact on PD risk in farm managers (sensitivity analysis 1) 5

Figure S4: Panel plot of the most important predictors and their impact on PD risk in farmworkers (sensitivity analysis 1) 6

Figure S5: Panel plot of the most important predictors and their impact on PD risk in farm managers (sensitivity analysis 2) 7

Figure S6: Panel plot of the most important predictors and their impact on PD risk in farmworkers (sensitivity analysis 2) 8

Figure S7: Panel plot of the most important predictors and their impact on PD risk in farm managers (sensitivity analysis 3) 9

Figure S8: Panel plot of the most important predictors and their impact on PD risk in farmworkers (sensitivity analysis 3) 10

Figure S9: Panel plot of the most important predictors and their impact on PD risk in farm managers (sensitivity analysis 4) 11

Figure S10: Panel plot of the most important predictors and their impact on PD risk in farmworkers (sensitivity analysis 4) 12

Figure S11: Panel plot of the most important predictors and their impact on PD risk in farm managers (sensitivity analysis 5) 13

Figure S12: Panel plot of the most important predictors and their impact on PD risk in farmworkers (sensitivity analysis 5) 14

Figure S13: Panel plot of the most important predictors and their impact on PD risk in farm managers (sensitivity analysis 6) 15

Figure S14: Panel plot of the most important predictors and their impact on PD risk in farmworkers (sensitivity analysis 6) 16

Figure S15: Comparison of the most important predictors and their impact on PD risk from the main analyses, sensitivity analysis and literature for farm managers 17

Figure S16: Comparison of the most important predictors and their impact on PD risk from the main analysis, sensitivity analyses and literature for farmworkers 18

Table S1: List and definition of farming activities available using MSA datasets for farm managers 19

Table S2: List and definition of agricultural activities available using MSA datasets for farmworkers 20

Table S3: Number of farmers with a LTI, ODC or ATC code of interest 21

Table S4: List of potential covariates considered in the analyses 22

Table S5: Baseline characteristics (sociodemographic and farm characteristics) of the FM population 24

Table S6: Baseline characteristics (medical covariates) of both farming populations 26

Table S7: Baseline characteristics (sociodemographic and farm characteristics) of the farmworker population. 28

Table S8: Best model hyperparameters and model tuning duration 29

Table S9: Mean absolute SHAP value of each model's feature, directionality of each feature's effect on PD risk, univariate association with Cox model, and comparison with the literature for the main analysis 30

Table S10: Mean absolute SHAP value of each model's feature, directionality of each feature's effect on PD risk, univariate association with Cox model, and comparison with the literature for the sensitivity analysis 1 32

Table S11: Mean absolute SHAP value of each model's feature, directionality of each feature's effect on PD risk, univariate association with Cox model, and comparison with the literature for the sensitivity analysis 2 34

Table S12: Mean absolute SHAP value of each model's feature, directionality of each feature's effect on PD risk, univariate association with Cox model, and comparison with the literature for the sensitivity analysis 3 36

Table S13: Mean absolute SHAP value of each model's feature, directionality of each feature's effect on PD risk, univariate association with Cox model, and comparison with the literature for the sensitivity analysis 4 38

Table S14: Mean absolute SHAP value of each model's feature, directionality of each feature's effect on PD risk, univariate association with Cox model, and comparison with the literature for the sensitivity analysis 5 40

Table S16: Mean absolute SHAP value of each model's feature, directionality of each feature's effect on PD risk, univariate association with Cox model, and comparison with the literature for the sensitivity analysis 6 42

RECORD Statement Checklist 43

References 46

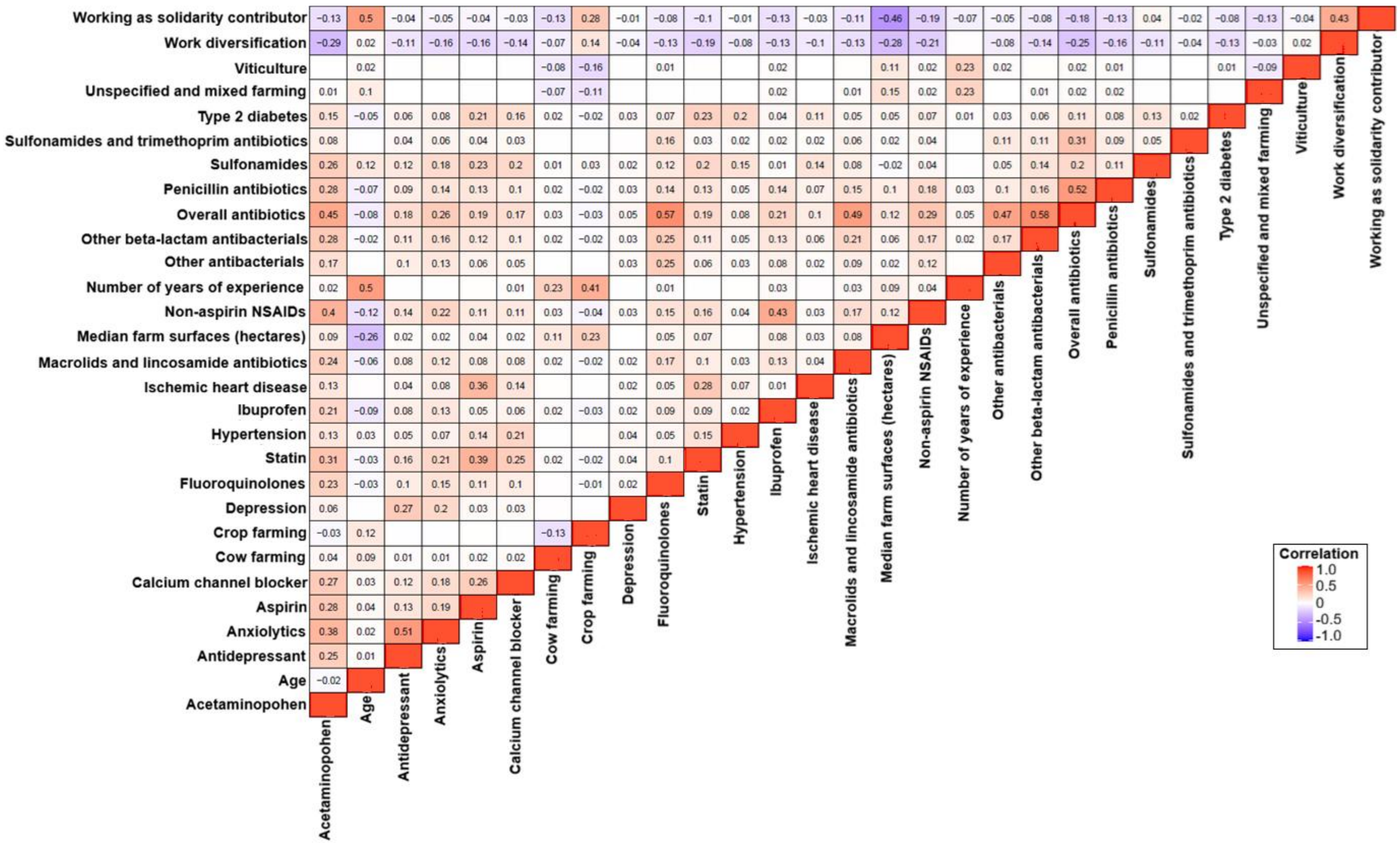

Figure S1: Correlation matrix of the main predictors for farm managers

Only features included in the final XGBoost models are represented in this correlation matrix to facilitate interpretation. To further ease reading, only predictors with an absolute correlation coefficient ≥ 0.20 were selected for display. White cells correspond to absolute correlation coefficients < 0.01. Cells with blue backgrounds indicate negative correlations, and cells with red backgrounds indicate positive correlations.

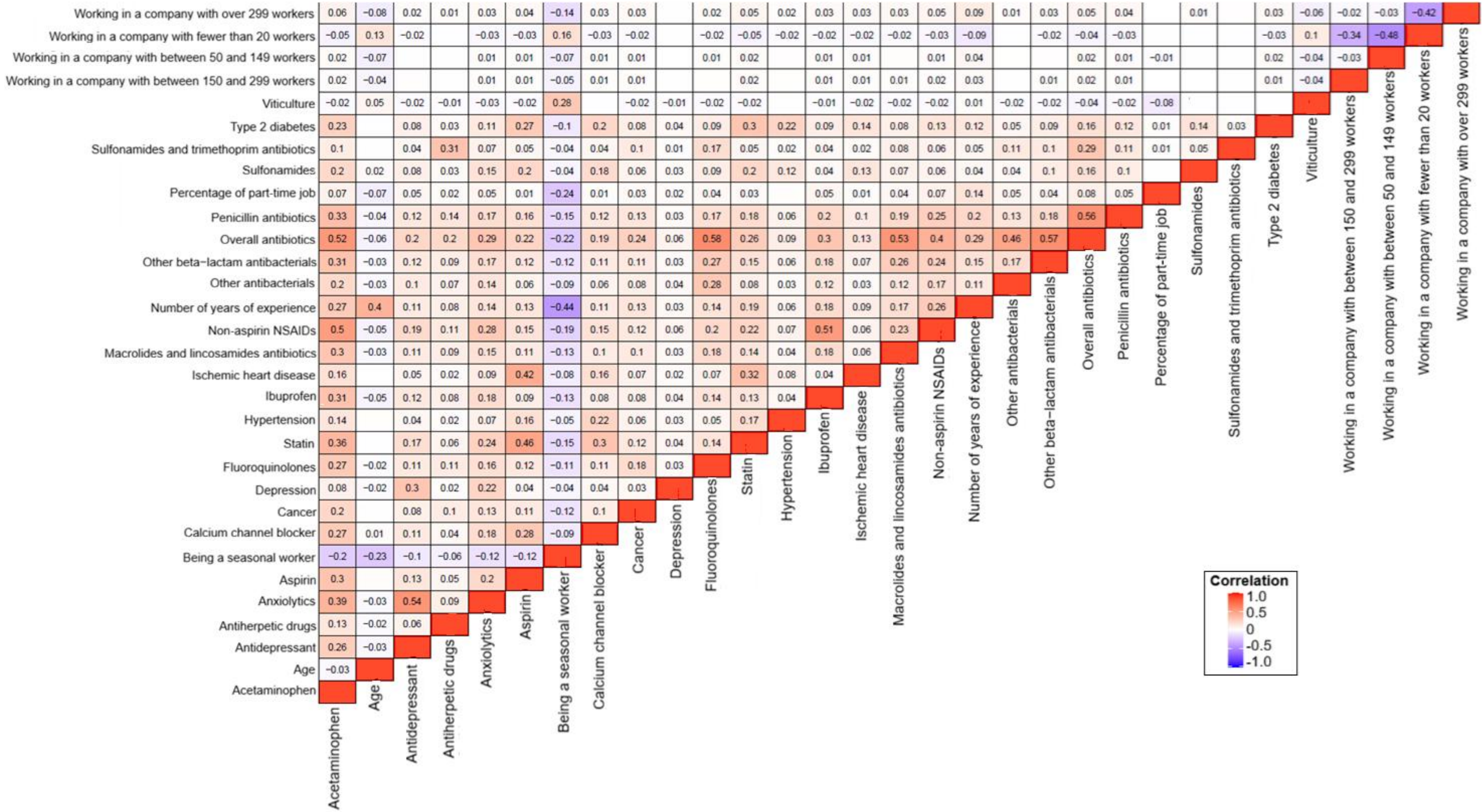

Figure S2: Correlation matrix of the main predictors for farmworkers

Only features included in the final XGBoost models are represented in this correlation matrix to facilitate interpretation. To further ease reading, only predictors with an absolute correlation coefficient ≥ 0.20 were selected for display. White cells correspond to absolute correlation coefficients < 0.01. Cells with blue backgrounds indicate negative correlations, and cells with red backgrounds indicate positive correlations.

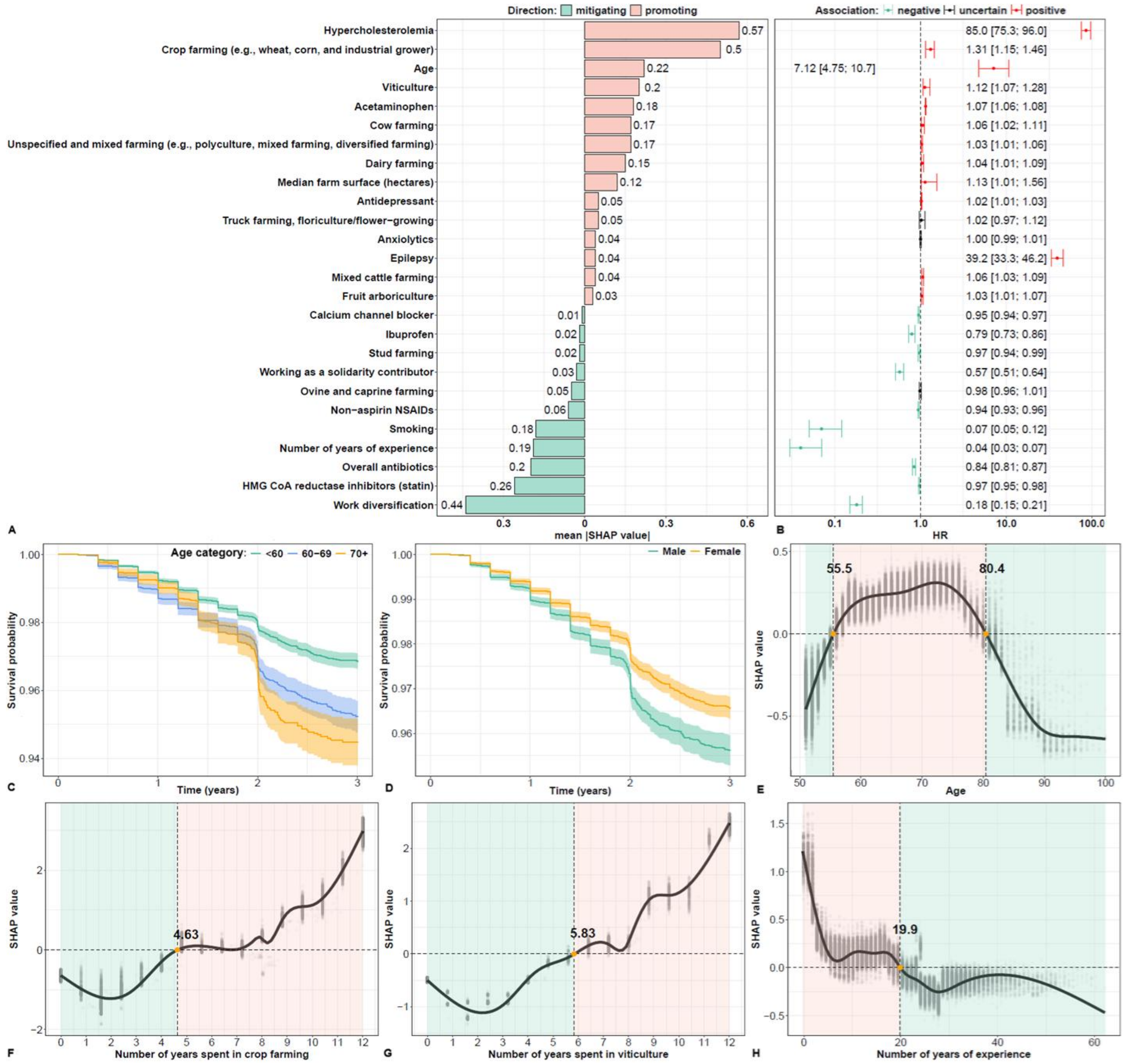

Figure S3: Panel plot of the most important predictors and their impact on PD risk in farm managers (sensitivity analysis 1)

SA1: application of an uniform 1-year exposure lag to all predictors.

A: Bar plot representing the most important predictors (mean |SHAP value| ≥ 0.01) ranked by mean absolute SHAP value. The color bar indicates the direction of association, with features showing positive SHAP contributions in red (promoting predictors) and those with negative contributions in green (mitigating predictors).

B: Error bar plot showing hazard ratios (HRs) and 95% confidence intervals for the corresponding key predictors from panel A. With the exception of age and sex, which were mutually adjusted, HRs were estimated using age- and sex-adjusted Cox proportional hazards models. The color of each error bar indicates the direction of association, with green for negative associations, black for uncertain associations (95% confidence interval includes 1), and red for positive associations.

C-D: Survival plots (Kaplan-Meier curve) stratified by age category and sex, respectively.

E-H: SHAP dependence plots for key predictors. Each grey point represents an individual FM. Background colors indicate regions of positive (red) and negative (green) SHAP values. The black line represents a smoothed trend estimated using a generalized additive model (GAM), and the orange point indicates where the SHAP value crosses zero.

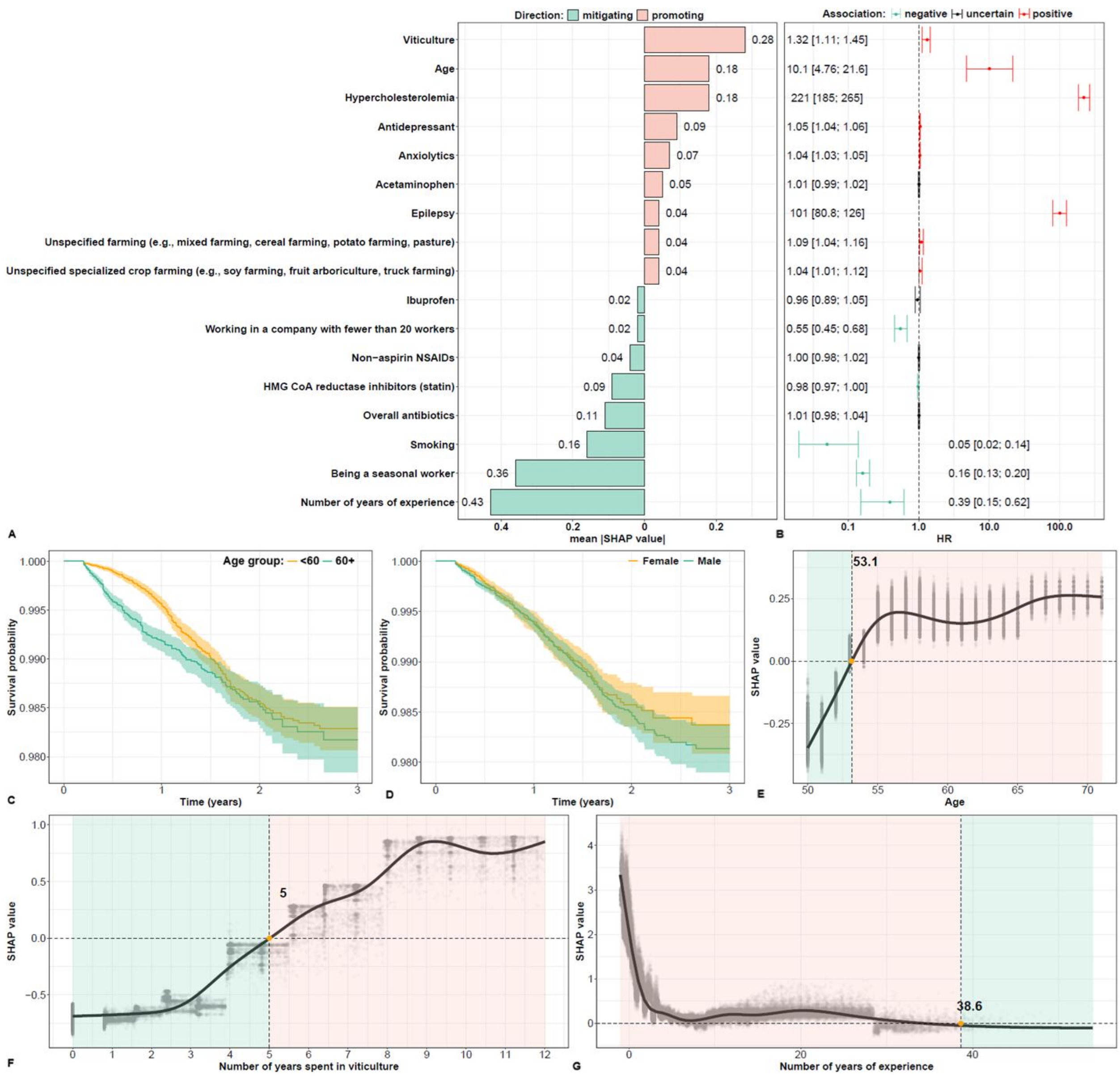

Figure S4: Panel plot of the most important predictors and their impact on PD risk in farmworkers (sensitivity analysis 1)

SA1: application of an uniform 1-year exposure lag to all predictors.

A: Bar plot representing the most important predictors (mean |SHAP value| ≥ 0.01) ranked by mean absolute SHAP value. The color bar indicates the direction of association, with features showing positive SHAP contributions in red (promoting predictors) and those with negative contributions in green (mitigating predictors).

B: Error bar plot showing hazard ratios (HRs) and 95% confidence intervals for the corresponding key predictors from panel A. With the exception of age and sex, which were mutually adjusted, HRs were estimated using age- and sex-adjusted Cox proportional hazards models. The color of each error bar indicates the direction of association, with green for negative associations, black for uncertain associations (95% confidence interval includes 1), and red for positive associations.

C-D: Survival plots (Kaplan-Meier curve) stratified by age category and sex, respectively.

E-G: SHAP dependence plots for key predictors. Each grey point represents an individual farmworker. Background colors indicate regions of positive (red) and negative (green) SHAP values. The black line represents a smoothed trend estimated using a generalized additive model (GAM), and the orange point indicates where the SHAP value crosses zero.

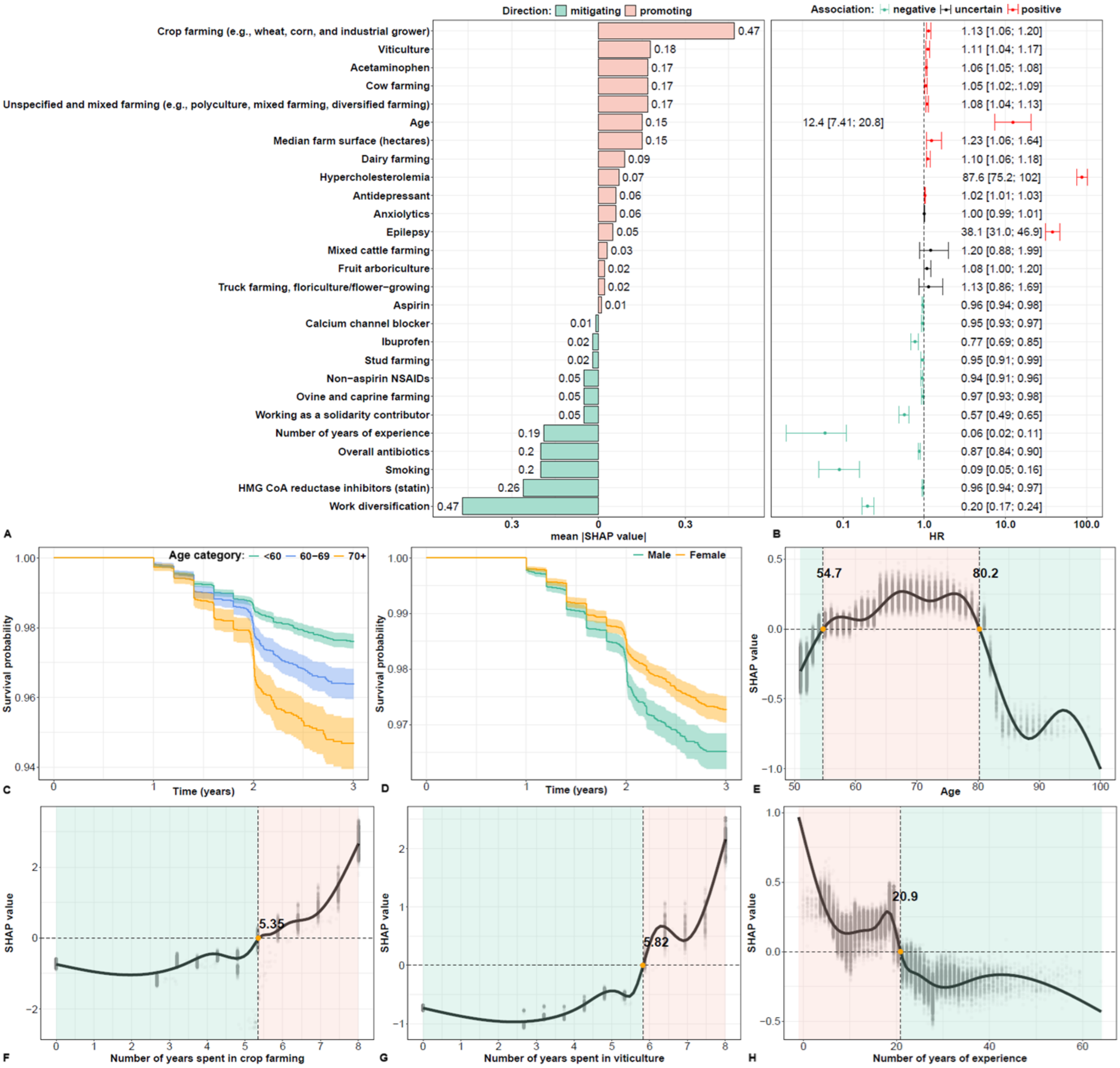

Figure S5: Panel plot of the most important predictors and their impact on PD risk in farm managers (sensitivity analysis 2)

SA2: application of a 1-year exposure lag to all health-related predictors and a 5-year exposure lag to all other predictors.

A: Bar plot representing the most important predictors (mean |SHAP value| ≥ 0.01) ranked by mean absolute SHAP value. The color bar indicates the direction of association, with features showing positive SHAP contributions in red (promoting predictors) and those with negative contributions in green (mitigating predictors).

B: Error bar plot showing hazard ratios (HRs) and 95% confidence intervals for the corresponding key predictors from panel A. With the exception of age and sex, which were mutually adjusted, HRs were estimated using age- and sex-adjusted Cox proportional hazards models. The color of each error bar indicates the direction of association, with green for negative associations, black for uncertain associations (95% confidence interval includes 1), and red for positive associations.

C-D: Survival plots (Kaplan-Meier curve) stratified by age category and sex, respectivel.

E-H: SHAP dependence plots for key predictors. Each grey point represents an individual FM. Background colors indicate regions of positive (red) and negative (green) SHAP values. The black line represents a smoothed trend estimated using a generalized additive model (GAM), and the orange point indicates where the SHAP value crosses zero.

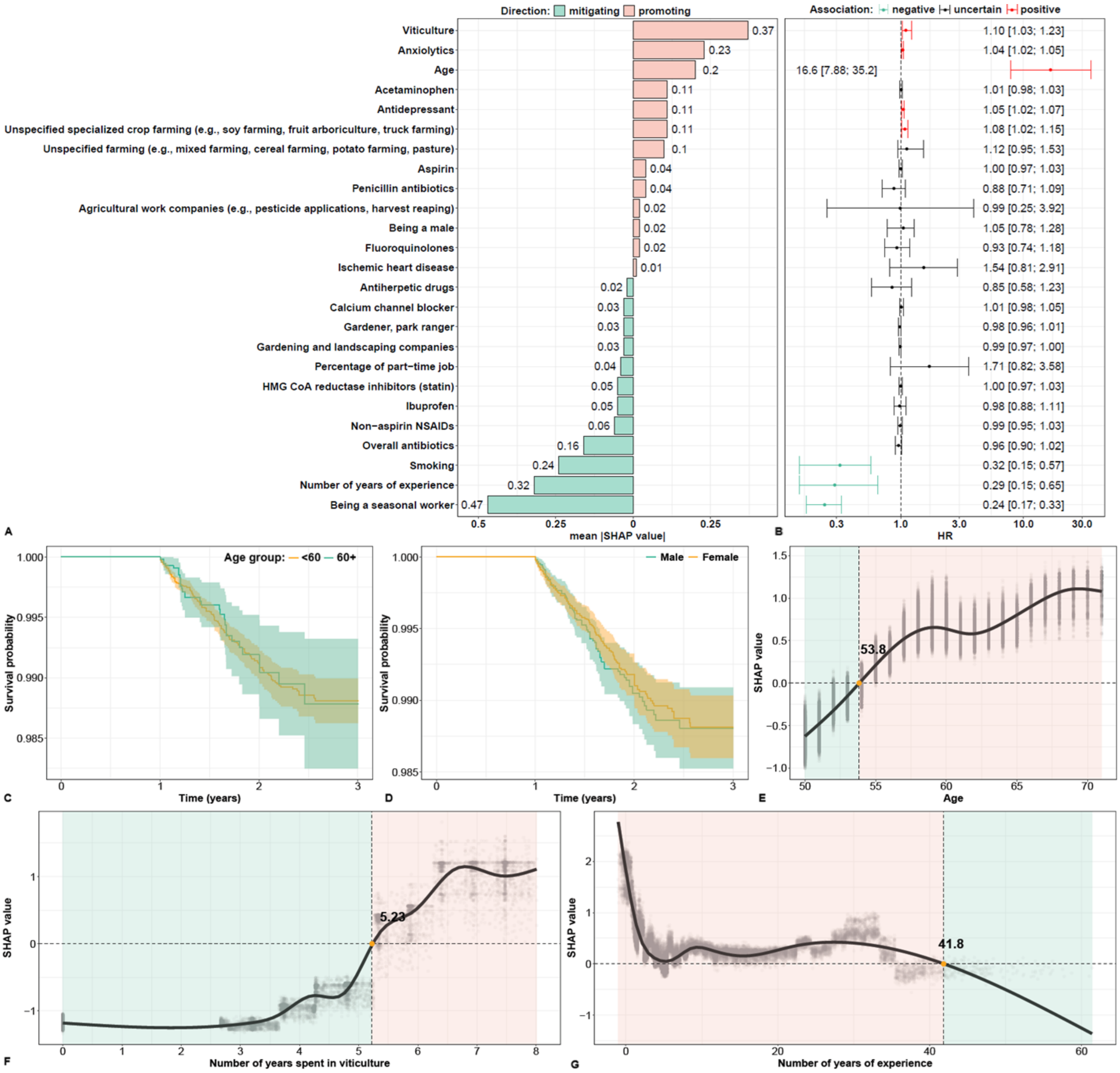

Figure S6: Panel plot of the most important predictors and their impact on PD risk in farmworkers (sensitivity analysis 2)

SA2: application of a 1-year exposure lag to all health-related predictors and a 5-year exposure lag to all other predictors.

A: Bar plot representing the most important predictors (mean |SHAP value| ≥ 0.01) ranked by mean absolute SHAP value. The color bar indicates the direction of association, with features showing positive SHAP contributions in red (promoting predictors) and those with negative contributions in green (mitigating predictors).
B: Error bar plot showing hazard ratios (HRs) and 95% confidence intervals for the corresponding key predictors from panel A. With the exception of age and sex, which were mutually adjusted, HRs were estimated using age- and sex-adjusted Cox proportional hazards models. The color of each error bar indicates the direction of association, with green for negative associations, black for uncertain associations (95% confidence interval includes 1), and red for positive associations.
C-D: Survival plots (Kaplan-Meier curve) stratified by age category and sex, respectively.
E-H: SHAP dependence plots for key predictors. Each grey point represents an individual farmworker. Background colors indicate regions of positive (red) and negative (green) SHAP values. The black line represents a smoothed trend estimated using a generalized additive model (GAM), and the orange point indicates where the SHAP value crosses zero.

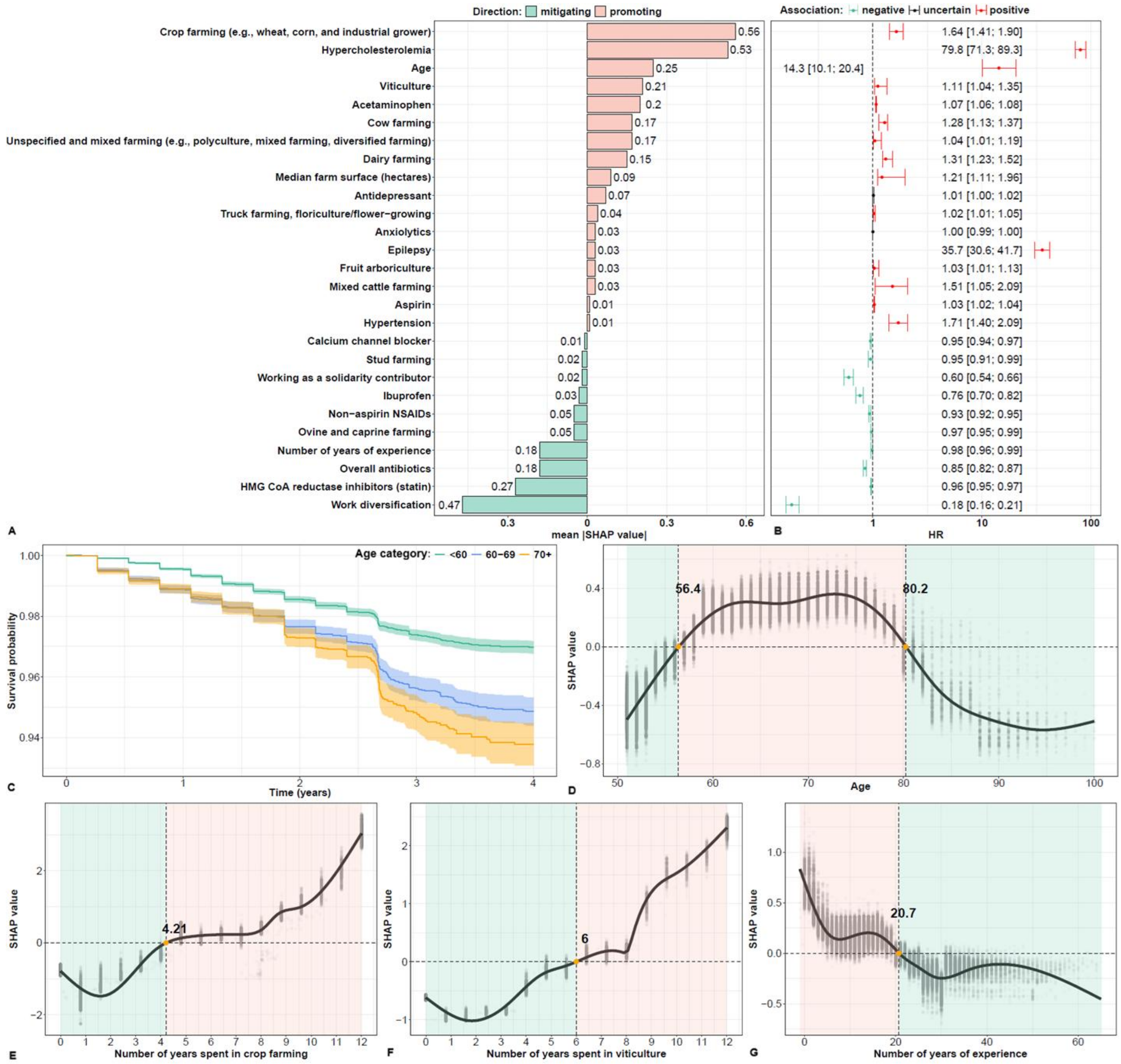

Figure S7: Panel plot of the most important predictors and their impact on PD risk in farm managers (sensitivity analysis 3)

SA3: not considering smoking and alcohol consumption.

A: Bar plot representing the most important predictors (mean |SHAP value| ≥ 0.01) ranked by mean absolute SHAP value. The color bar indicates the direction of association, with features showing positive SHAP contributions in red (promoting predictors) and those with negative contributions in green (mitigating predictors).

B: Error bar plot showing hazard ratios (HRs) and 95% confidence intervals for the corresponding key predictors from panel A. With the exception of age and sex, which were mutually adjusted, HRs were estimated using age- and sex-adjusted Cox proportional hazards models. The color of each error bar indicates the direction of association, with green for negative associations, black for uncertain associations (95% confidence interval includes 1), and red for positive associations.

C: Survival plot (Kaplan-Meier curve) stratified by age category.

E-G: SHAP dependence plots for key predictors. Each grey point represents an individual FM. Background colors indicate regions of positive (red) and negative (green) SHAP values. The black line represents a smoothed trend estimated using a generalized additive model (GAM), and the orange point indicates where the SHAP value crosses zero.

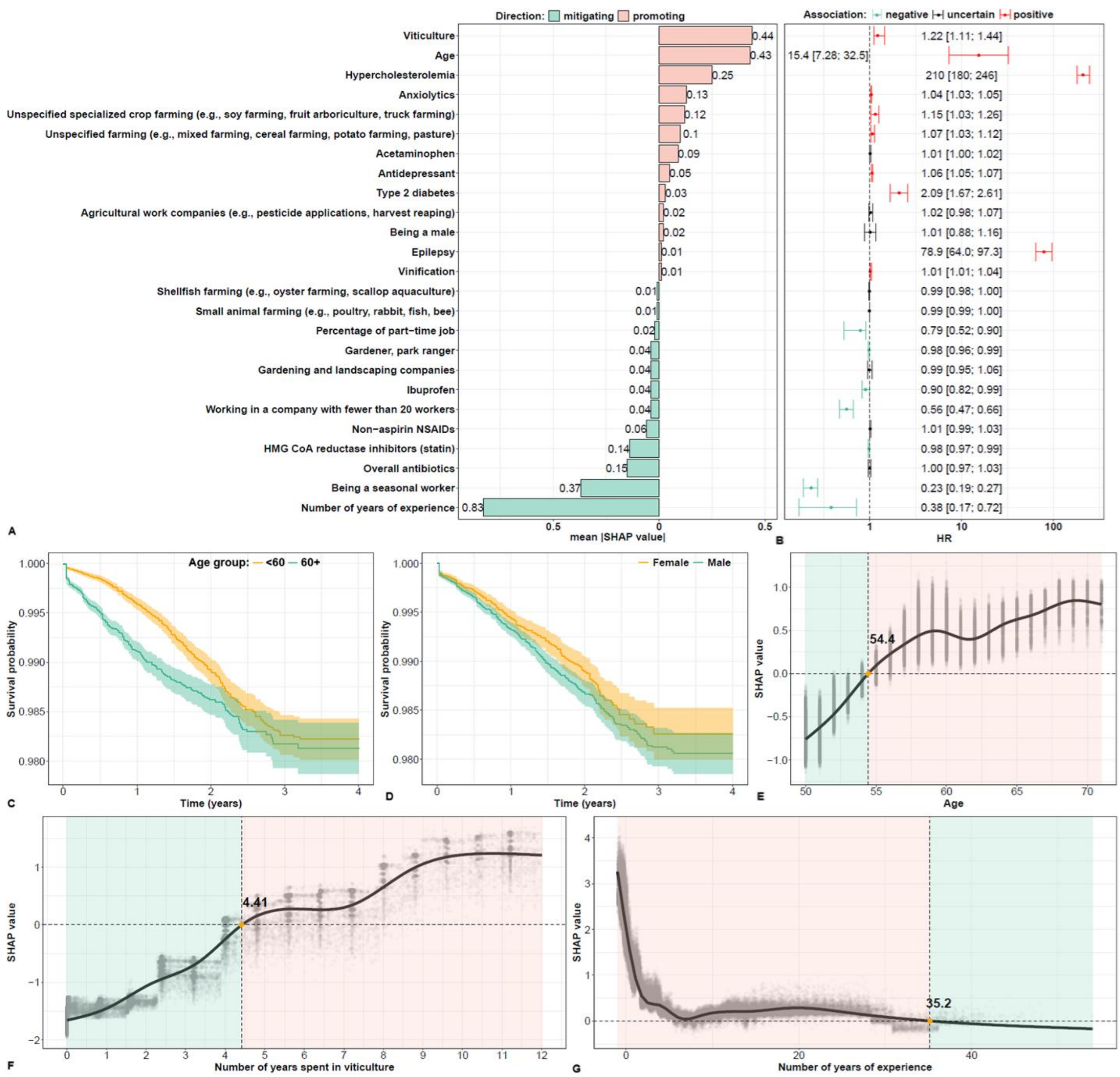

Figure S8: Panel plot of the most important predictors and their impact on PD risk in farmworkers (sensitivity analysis 3)

SA3: not considering smoking and alcohol consumption.

A: Bar plot representing the most important predictors (mean |SHAP value| ≥ 0.01) ranked by mean absolute SHAP value. The color bar indicates the direction of association, with features showing positive SHAP contributions in red (promoting predictors) and those with negative contributions in green (mitigating predictors).

B: Error bar plot showing hazard ratios (HRs) and 95% confidence intervals for the corresponding key predictors from panel A. With the exception of age and sex, which were mutually adjusted, HRs were estimated using age- and sex-adjusted Cox proportional hazards models. The color of each error bar indicates the direction of association, with green for negative associations, black for uncertain associations (95% confidence interval includes 1), and red for positive associations.

C-D: Survival plots (Kaplan-Meier curve) stratified by age category and sex, respectively.

E-H: SHAP dependence plots for key predictors. Each grey point represents an individual farmworker. Background colors indicate regions of positive (red) and negative (green) SHAP values. The black line represents a smoothed trend estimated using a generalized additive model (GAM), and the orange point indicates where the SHAP value crosses zero.

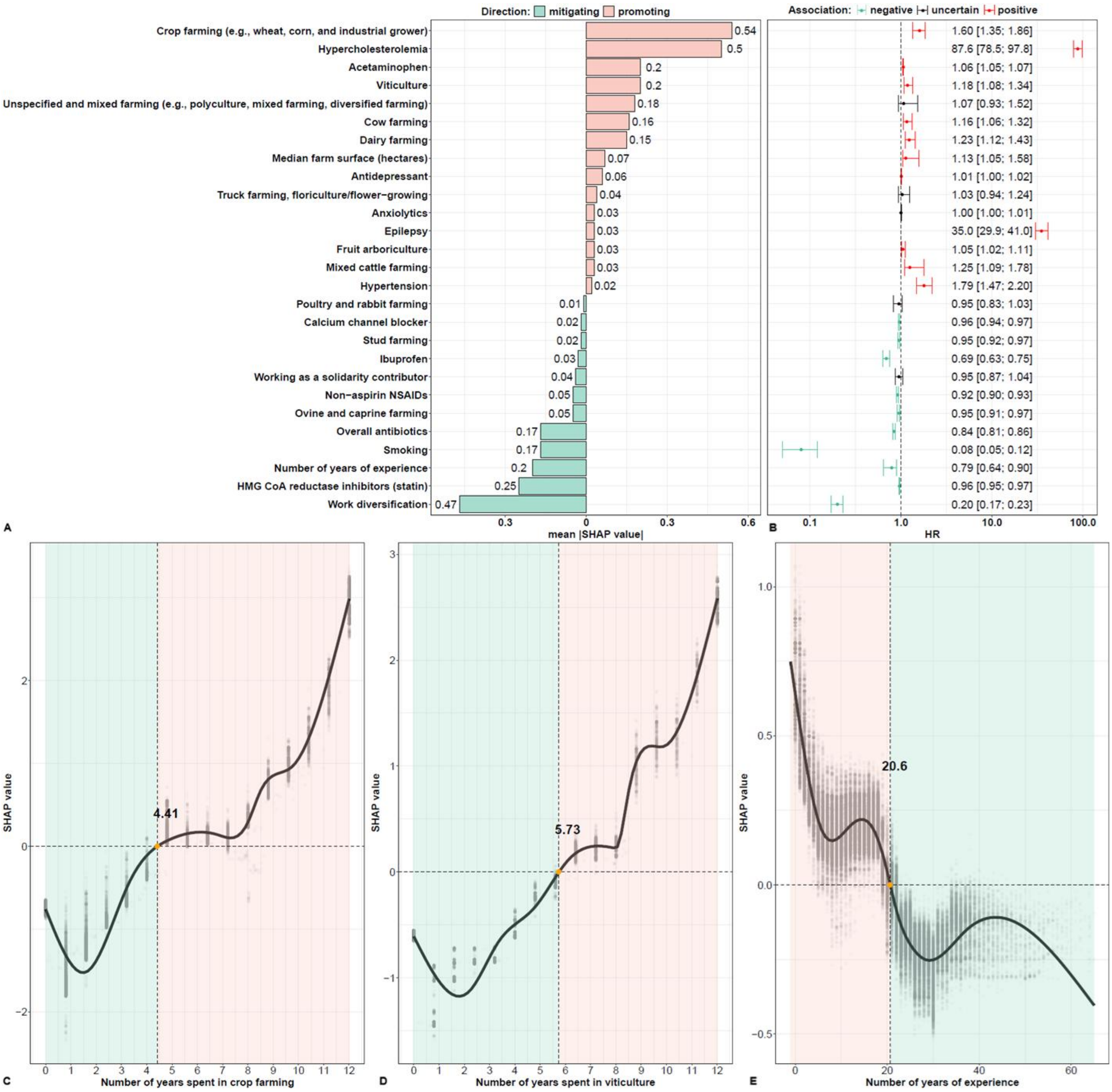

Figure S9: Panel plot of the most important predictors and their impact on PD risk in farm managers (sensitivity analysis 4)

SA4: not considering age and sex.

A: Bar plot representing the most important predictors (mean |SHAP value| ≥ 0.01) ranked by mean absolute SHAP value. The color bar indicates the direction of association, with features showing positive SHAP contributions in red (promoting predictors) and those with negative contributions in green (mitigating predictors).

B: Error bar plot showing hazard ratios (HRs) and 95% confidence intervals for the corresponding key predictors from panel A. With the exception of age and sex, which were mutually adjusted, HRs were estimated using age- and sex-adjusted Cox proportional hazards models. The color of each error bar indicates the direction of association, with green for negative associations, black for uncertain associations (95% confidence interval includes 1), and red for positive associations.

C-E: SHAP dependence plots for key predictors. Each grey point represents an individual FM. Background colors indicate regions of positive (red) and negative (green) SHAP values. The black line represents a smoothed trend estimated using a generalized additive model (GAM), and the orange point indicates where the SHAP value crosses zero.

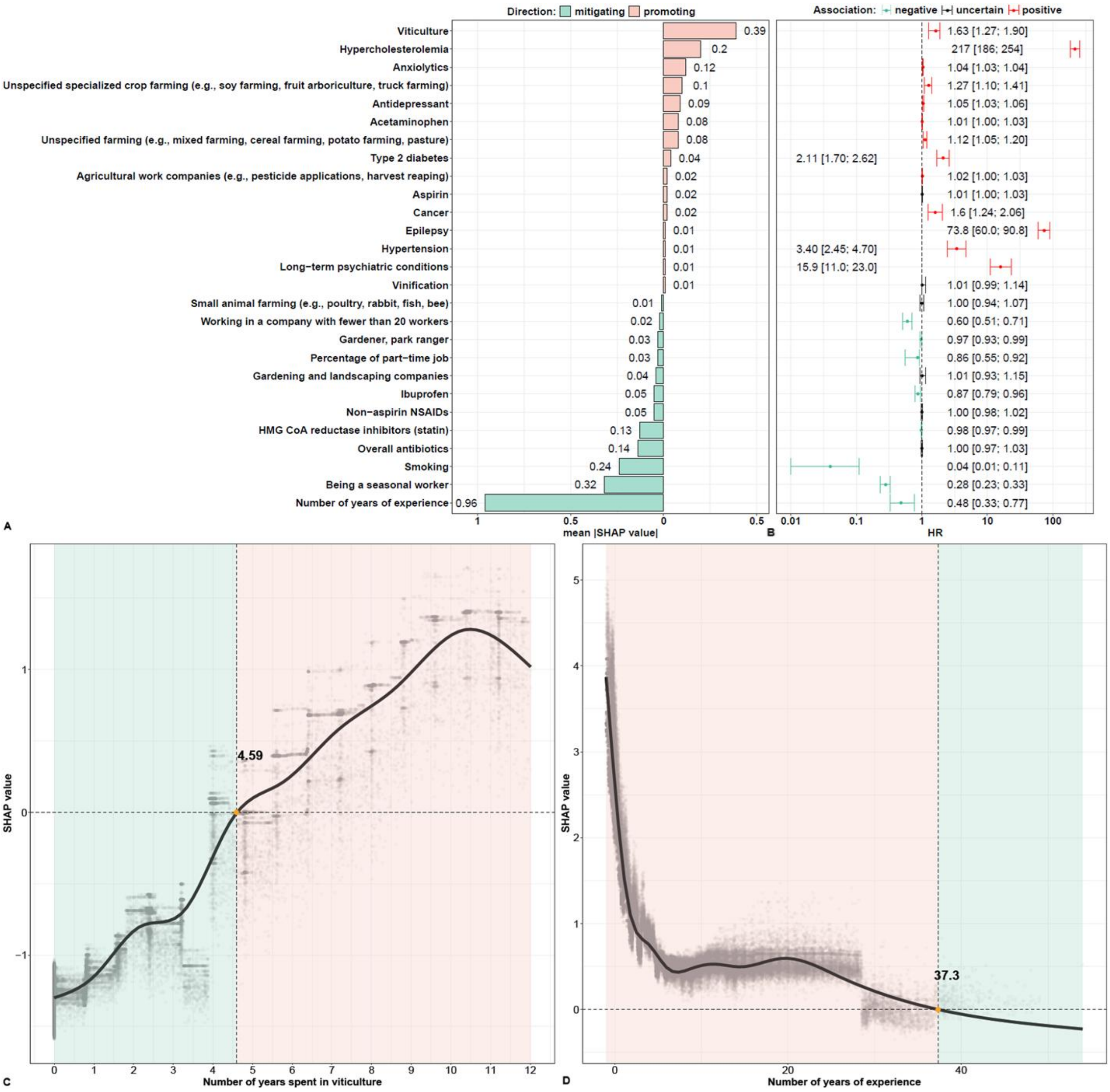

Figure S10: Panel plot of the most important predictors and their impact on PD risk in farmworkers (sensitivity analysis 4)

SA4: not considering age and sex.

A: Bar plot representing the most important predictors (mean |SHAP value| ≥ 0.01) ranked by mean absolute SHAP value. The color bar indicates the direction of association, with features showing positive SHAP contributions in red (promoting predictors) and those with negative contributions in green (mitigating predictors).

B: Error bar plot showing hazard ratios (HRs) and 95% confidence intervals for the corresponding key predictors from panel A. With the exception of age and sex, which were mutually adjusted, HRs were estimated using age- and sex-adjusted Cox proportional hazards models. The color of each error bar indicates the direction of association, with green for negative associations, black for uncertain associations (95% confidence interval includes 1), and red for positive associations.

C-D: SHAP dependence plots for key predictors. Each grey point represents an individual farmworker. Background colors indicate regions of positive (red) and negative (green) SHAP values. The black line represents a smoothed trend estimated using a generalized additive model (GAM), and the orange point indicates where the SHAP value crosses zero.

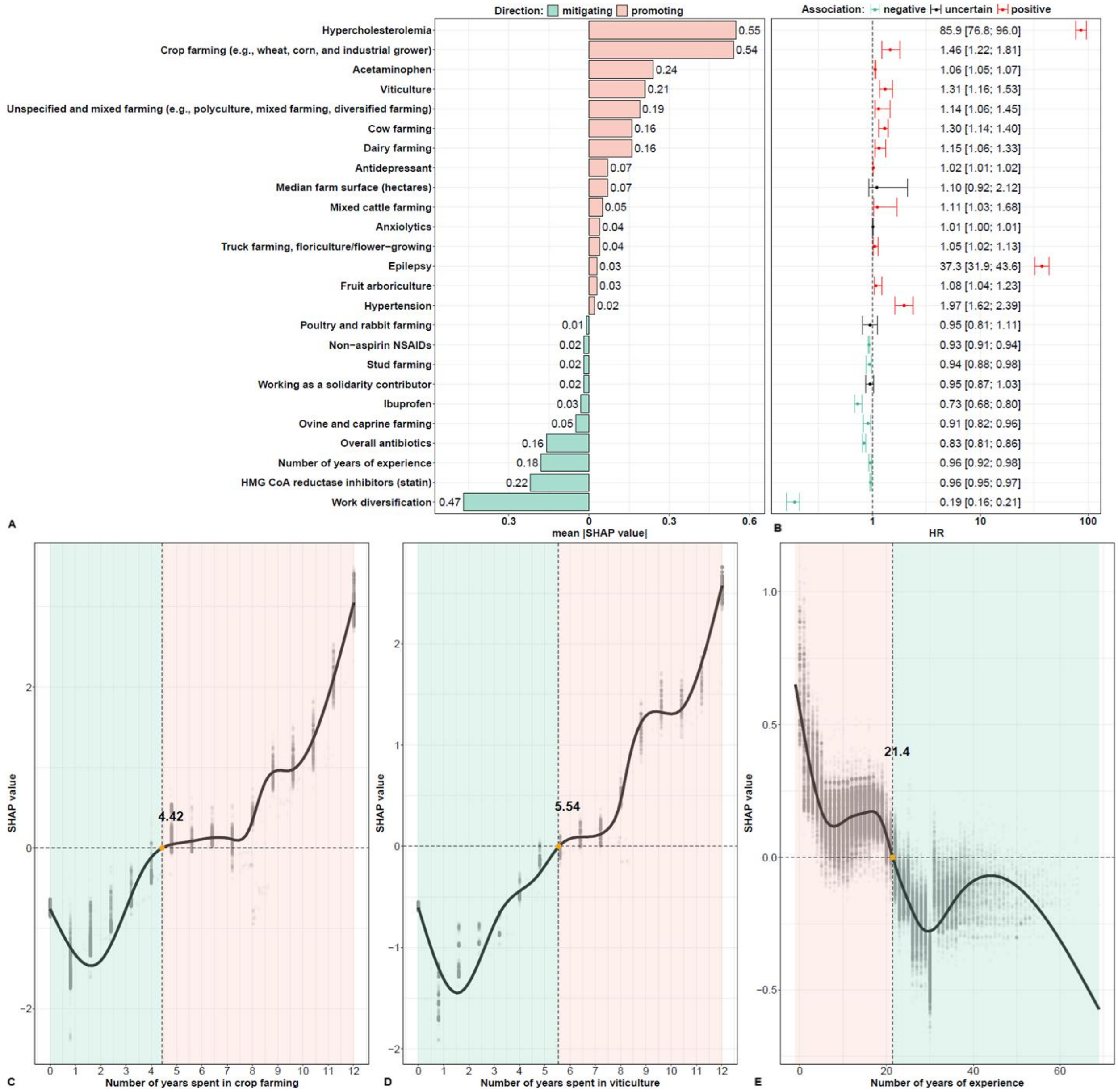

Figure S11: Panel plot of the most important predictors and their impact on PD risk in farm managers (sensitivity analysis 5)

SA5: not considering age, sex, alcohol consumption and smoking.

A: Bar plot representing the most important predictors (mean |SHAP value| ≥ 0.01) ranked by mean absolute SHAP value. The color bar indicates the direction of association, with features showing positive SHAP contributions in red (promoting predictors) and those with negative contributions in green (mitigating predictors).

B: Error bar plot showing hazard ratios (HRs) and 95% confidence intervals for the corresponding key predictors from panel A. With the exception of age and sex, which were mutually adjusted, HRs were estimated using age- and sex-adjusted Cox proportional hazards models. The color of each error bar indicates the direction of association, with green for negative associations, black for uncertain associations (95% confidence interval includes 1), and red for positive associations.

C-E: SHAP dependence plots for key predictors. Each grey point represents an individual FM. Background colors indicate regions of positive (red) and negative (green) SHAP values. The black line represents a smoothed trend estimated using a generalized additive model (GAM), and the orange point indicates where the SHAP value crosses zero.

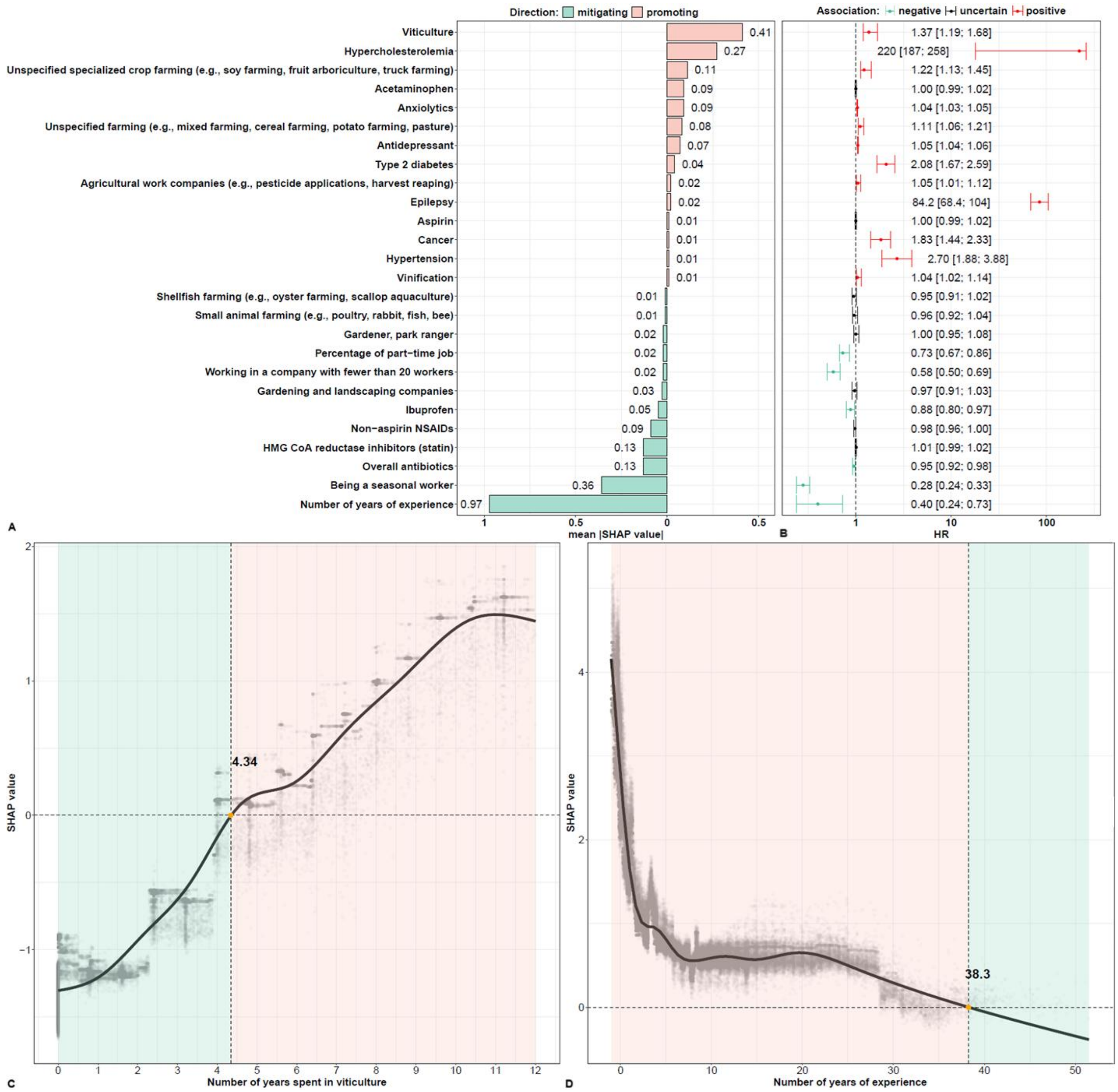

Figure S12: Panel plot of the most important predictors and their impact on PD risk in farmworkers (sensitivity analysis 5)

SA5: not considering age, sex, alcohol consumption and smoking.

A: Bar plot representing the most important predictors (mean |SHAP value| ≥ 0.01) ranked by mean absolute SHAP value. The color bar indicates the direction of association, with features showing positive SHAP contributions in red (promoting predictors) and those with negative contributions in green (mitigating predictors).

B: Error bar plot showing hazard ratios (HRs) and 95% confidence intervals for the corresponding key predictors from panel A. With the exception of age and sex, which were mutually adjusted, HRs were estimated using age- and sex-adjusted Cox proportional hazards models. The color of each error bar indicates the direction of association, with green for negative associations, black for uncertain associations (95% confidence interval includes 1), and red for positive associations.

C-D: SHAP dependence plots for key predictors. Each grey point represents an individual FM. Background colors indicate regions of positive (red) and negative (green) SHAP values. The black line represents a smoothed trend estimated using a generalized additive model (GAM), and the orange point indicates where the SHAP value crosses zero.

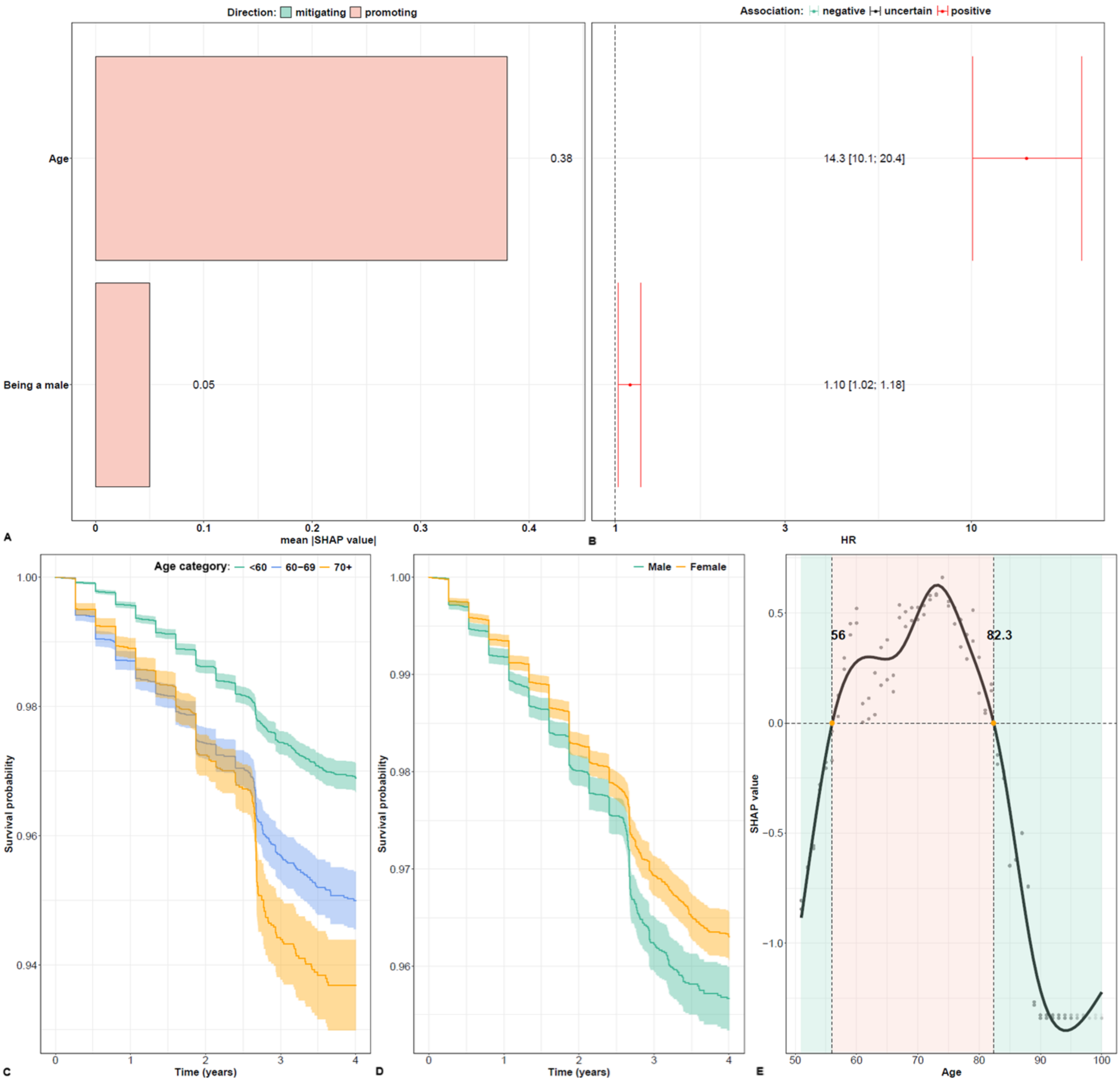

Figure S13: Panel plot of the most important predictors and their impact on PD risk in farm managers (sensitivity analysis 6)

SA6: baseline model including only age and sex.

A: Bar plot representing the most important predictors (mean |SHAP value| ≥ 0.01) ranked by mean absolute SHAP value. The color bar indicates the direction of association, with features showing positive SHAP contributions in red (promoting predictors) and those with negative contributions in green (mitigating predictors).

B: Error bar plot showing hazard ratios (HRs) and 95% confidence intervals for the corresponding key predictors from panel A. With the exception of age and sex, which were mutually adjusted, HRs were estimated using age- and sex-adjusted Cox proportional hazards models. The color of each error bar indicates the direction of association, with green for negative associations, black for uncertain associations (95% confidence interval includes 1), and red for positive associations.

C: Survival plots (Kaplan-Meier curves) stratified by age category and sex, respectively.

D-E: SHAP dependence plots for key predictors. Each grey point represents an individual FM. Background colors indicate regions of positive (red) and negative (green) SHAP values. The black line represents a smoothed trend estimated using a generalized additive model (GAM), and the orange point indicates where the SHAP value crosses zero.

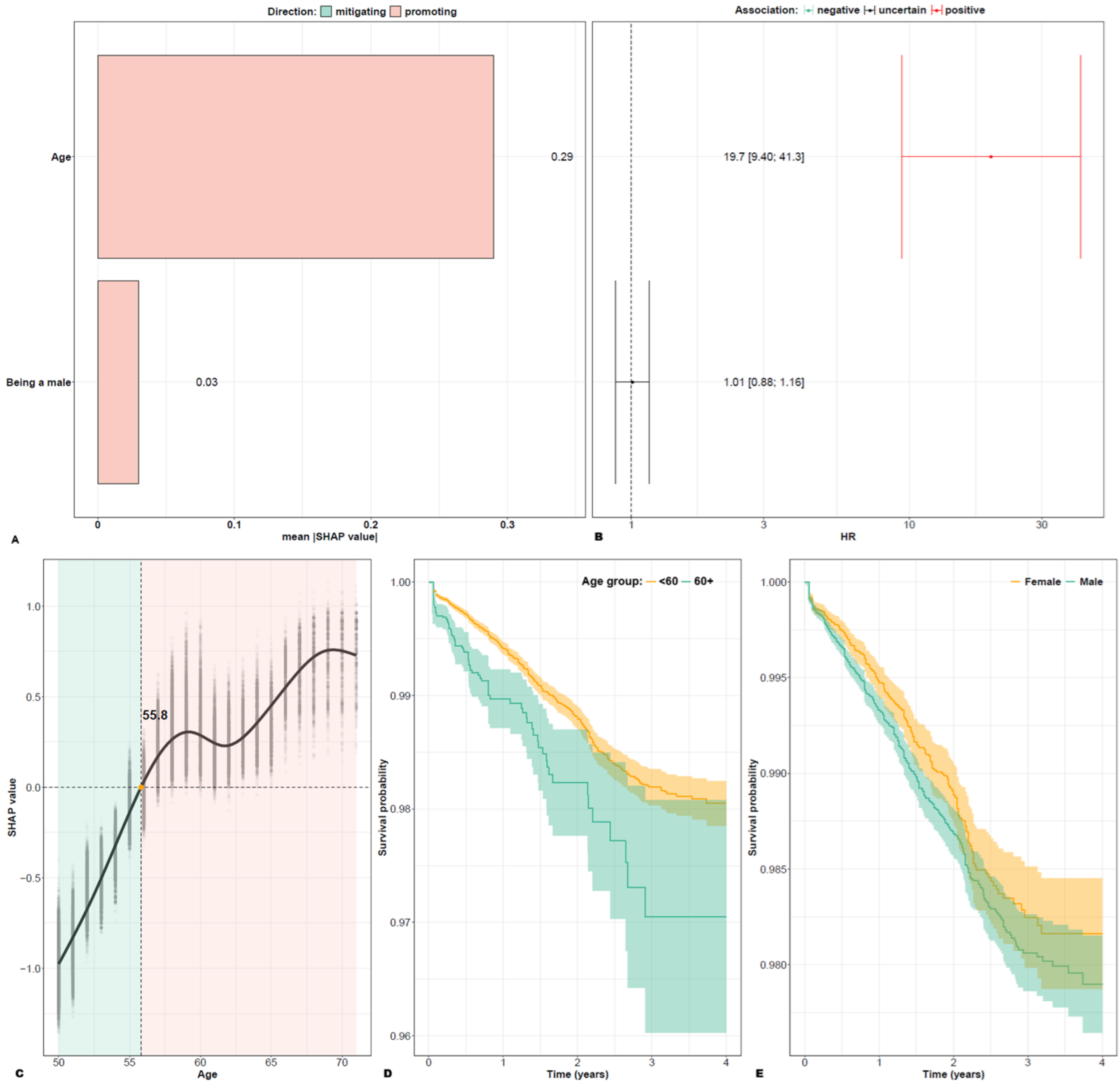

Figure S14: Panel plot of the most important predictors and their impact on PD risk in farmworkers (sensitivity analysis 6)

SA6: baseline model including only age and sex.

A: Bar plot representing the most important predictors (mean |SHAP value| ≥ 0.01) ranked by mean absolute SHAP value. The color bar indicates the direction of association, with features showing positive SHAP contributions in red (promoting predictors) and those with negative contributions in green (mitigating predictors).

B: SHAP dependence plots for key predictors. Each grey point represents an individual farmworker. Background colors indicate regions of positive (red) and negative (green) SHAP values. The black line represents a smoothed trend estimated using a generalized additive model (GAM), and the orange point indicates where the SHAP value crosses zero.

C: Dependance plot for age. Each grey point represents an individual farmworker. The light green and red backgrounds denote regions of positive and negative SHAP values, respectively. The black line represents a smoothed trend estimated by a generalized additive model (GAM), with the orange point indicating where the SHAP value crosses zero.

D-E: Survival plots (Kaplan-Meier curves) stratified by age category and sex, respectively.

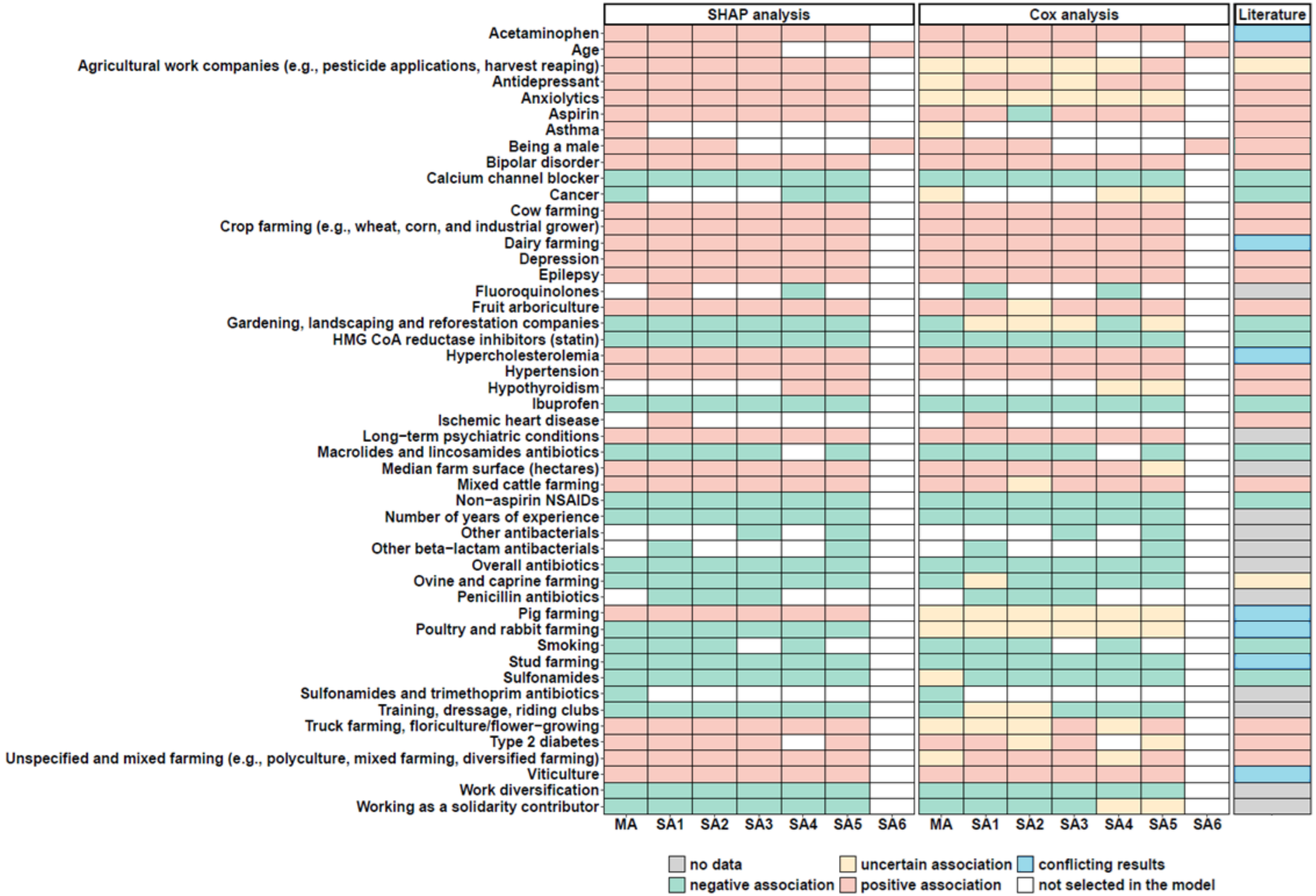

Figure S15: Comparison of the most important predictors and their impact on PD risk from the main analyses, sensitivity analysis and literature for farm managers

The association direction is represented with the background color of each cell. MA: main analysis. SA1: application of an uniform 1-year exposure lag to all predictors. SA2: application of a 1-year exposure lag to all health-related predictors and a 5-year exposure lag to all other predictors. SA3: not considering smoking and alcohol consumption. SA4: not considering age and sex. SA5: not considering age, sex, smoking and alcohol consumption. SA6: baseline model including only age and sex.

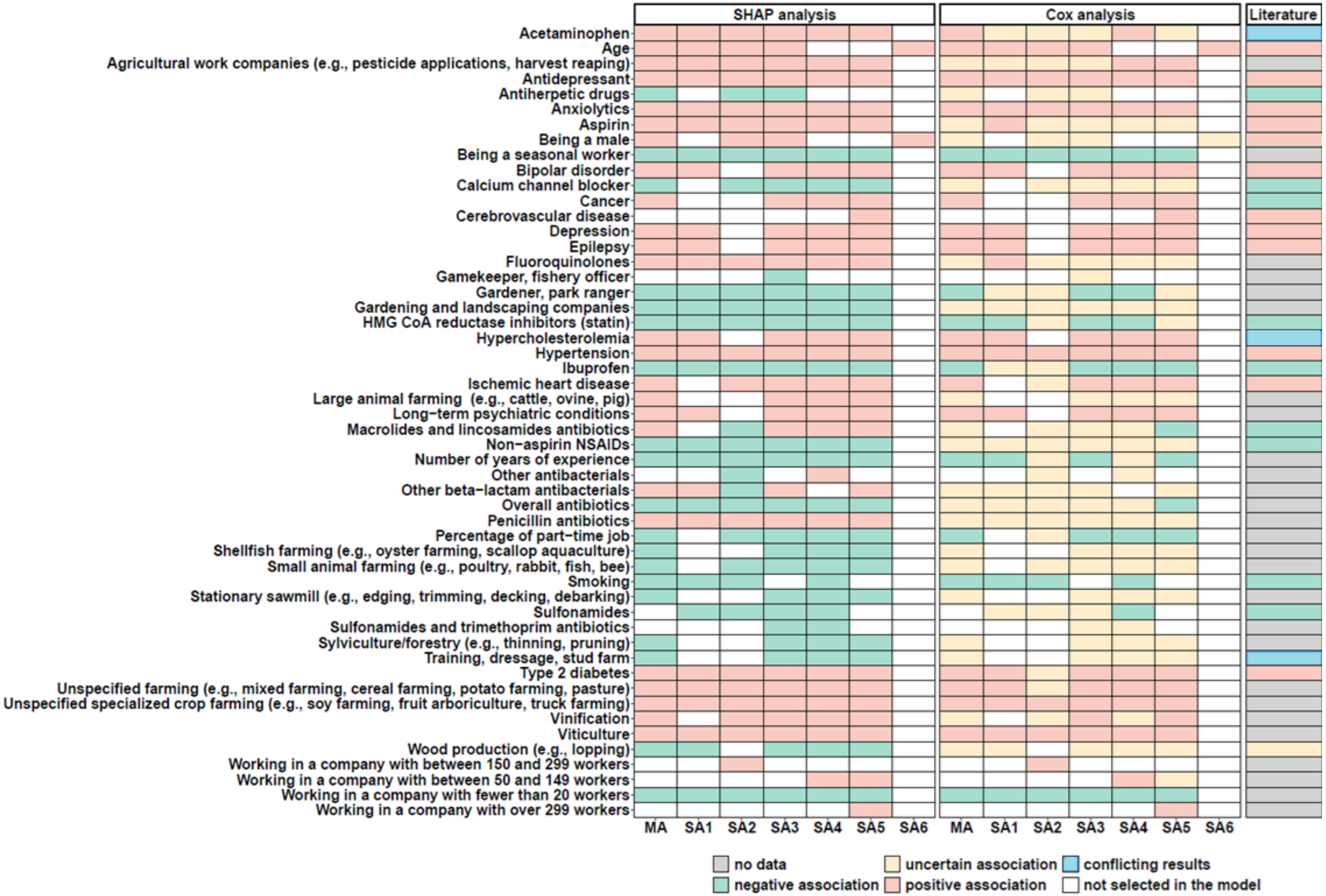

Figure S16: Comparison of the most important predictors and their impact on PD risk from the main analysis, sensitivity analyses and literature for farmworkers

The association direction is represented with the background color of each cell. MA: main analysis. SA1: application of an uniform 1-year exposure lag to all predictors. SA2: application of a 1-year exposure lag to all health-related predictors and a 5-year exposure lag to all other predictors. SA3: not considering smoking and alcohol consumption. SA4: not considering age and sex. SA5: not considering age, sex, smoking and alcohol consumption. SA6: baseline model including only age and sex.

Table S1: List and definition of farming activities available using MSA datasets for farm managers

Activity name	Definition	Example of task
Overall population	When considering all farming activities	Crop farming, dairy farming
Truck farming, floriculture/flower-growing	Practice of growing and harvesting fruits, vegetables, and flowers for sale	Proper crop selection, soil and water management, pest and disease management, harvesting, and post-harvest handling
Fruit arboriculture	Cultivation of fruits growing on trees	Pruning branches, removing damaged or diseased branches, fertilizing trees, monitoring, and controlling pests and diseases
Garden center/tree nursery	Propagation, breeding and growing of plants and trees to a desired size	Planting, watering, weeding, digging, and using tools such as a sieve, or spade
Crop farming (e.g., wheat, corn, and industrial grower)	Cultivation of plants (e.g. cereals, vegetables) for food	Monitoring and controlling pests and diseases, fertilizing, harvesting and post-harvest handling, grading, soil and water management, tractor driving
Viticulture	Cultivation and harvesting of grapes, and winemaking	Monitoring and controlling pests and diseases, fertilizing, soil and water management, canopy management, monitoring fruit development and characteristics, harvesting and post-harvest handling, and vine pruning
Sylviculture/forestry (e.g., thinning, pruning)	Controlling the growth, composition/structure, as well as quality of forests	Thinning, pruning, and spacing
Unspecified specialized farming (e.g., herbs, mushrooms)	Cultivation and harvesting of herbs, spices, medical plants, and mushrooms	Layering, cutting, grafting, pruning, mulching, and weed management
Dairy farming	Raising and breeding of domestic cows for milk production	Breeding, milking (manually, or using a machine), feeding, bulk handling, disease prevention, and managing waste
Cow farming	Raising and breeding of domestic cows as livestock	Feeding, maintaining facilities, monitoring the herd for signs of illness, assisting with calving, performing artificial insemination, and managing waste
Both/mixed dairy and cow farming	Raising and breeding of domestic cows as livestock and for milk production	Breeding, milking, feeding, bulk handling, and disease prevention
Ovine and caprine farming	Raising and breeding of domestic sheep and goats	Breeding, milking, wool production, maintenance of the property, mustering, milking, shearing and drenching livestock, sterilizing machines, and collecting, grading and packaging produce
Pig farming	Raising and breeding of domestic pigs as livestock	Maintain equipment and buildings, spread manure, feeding, monitoring the pigs for signs of illness, assist in artificial insemination, and transporting stock to farms or processing plants
Stud farming	Raising and breeding of domestic horses	Feeding, breeding, assist in artificial insemination, and disease prevention
Unspecified large animal farming (e.g., ostrich, llama)	Raising and breeding of large animals such as wild animals, ostrich, bison, boar, or llama	Feeding, breeding, and disease prevention
Poultry and rabbit farming	Raising and breeding of domestic birds and rabbits	Feeding, breeding, disease prevention, assist in artificial insemination, gathering eggs, cleaning rooms and equipment/facility maintenance
Unspecified small animal farming (e.g., frogs, snails, bees)	Raising and breeding of small animals such as frogs, bees, insects, minks, beavers, or snails	Feeding, breeding, and disease prevention
Training, dressage, riding clubs	Activity including training and dressage of domestic equines	Feeding, disease prevention, teaching, maintenance of the property, and tourism
Shellfish farming (e.g., oyster farming, scallop aquaculture)	Cultivation and harvest of aquatic invertebrates, such as oysters, clams, and mussels	Breeding, rearing, harvesting, using a rack, processing, and shipment
Unspecified and mixed farming (e.g., polyculture, mixed farming, diversified farming)	Activity involving both the growing of crops and the raising of livestock, or the growing of more than one crop species	Planning and coordinating the production and marketing of crops and livestock, breeding and raising livestock for the production of meat, wool, skins, milk or eggs, and growing grain, seed crops, vegetables, fruit or nuts
Salt works/salt evaporation pond	Production of salt from sea water	Harvesting, and filtering
Wood production (e.g., lopping)	Harvesting of round wood (trunk and branch wood) from coniferous (softwood) and non-coniferous (hardwood) trees	Harvesting, lopping, and transport
Stationary sawmill (e.g., edging, trimming, decking, debarking)	Cutting logs into lumber using large bandsaw blades	Edging, trimming, decking, debarking, use of machines
Agricultural work companies (e.g., pesticide applications, harvest reaping)	Companies that assist or perform farm works such as harvest reaping	Harvest reaping, harrowing, sowing seeds, pesticide application, tilling, and clearing
Gardening, landscaping and reforestation companies	Companies that perform gardening, landscaping and reforestation work	Gardening, landscaping, chainsaw and skidder operation, climbing on trees, monitoring and controlling pests and diseases
Company representative/authorized representative	Self-employed insurance broker responsible for representing a legal entity (agricultural insurance company)	Traveling, administrative tasks, and visiting farms
Rural craftsperson (e.g., mason, mechanics)	Self-employed person performing construction or hand-crafted work in rural areas, including jobs such as well-diggers, painters, electricians, masons, carpenters, saddlers, blacksmiths, mechanics, wheelwright, or farriers	Fixing agricultural machinery, painting, welding, and hammering

Table S2: List and definition of agricultural activities available using MSA datasets for farmworkers

Activity name	Definition	Example of task
Overall population	When considering all farming activities	Crop farming, dairy farming
Unspecified farming (e.g., mixed farming, cereal farming, potato farming, pasture)	Cultivation of plants (e.g., cereals, potatoes, rice) as well as grassland and grazing operations	Monitoring and controlling pests and diseases, fertilizing, harvesting and post-harvest handling, grading, soil and water management, tractor driving
Viticulture	Cultivation and harvesting of grapes, and winemaking	Monitoring and controlling pests and diseases, fertilizing, soil and water management, canopy management, monitoring fruit development and characteristics, harvesting and post-harvest handling, and vine pruning
Vinification	Production of wine	Selection of the fruit, fruit fermentation into alcohol, and bottling of the finished liquid
Sylviculture/forestry (e.g., thinning, pruning)	Controlling the growth, composition/structure, as well as quality of forests	Thinning, pruning, and spacing
Resin extraction	Incising the outer layers of a pine tree in order to collect the sap or resin	Tool handling
Unspecified specialized crop farming (e.g., soy farming, fruit arboriculture, truck farming)	Cultivation of plants (e.g., vegetables) and fruits for food as well as practice of growing and harvesting fruits, vegetables, and flowers for sale	Layering, cutting, grafting, pruning, mulching, weed management, removing damaged or diseased branches, fertilizing trees, monitoring, controlling pests and diseases, proper crop selection, soil and water management, harvesting, and post-harvest handling
Mushroom farming	Cultivation of mushrooms for food	Planting, pest and disease management, harvesting, and post-harvest handling
Large animal farming (e.g., cattle, ovine, pig)	Raising and breeding of large animals such as wild cattle, pig, ovine, or llama as livestock and for products (e.g., milk) production	Maintain equipment and buildings, breeding, milking, wool production, maintenance of the property, mustering, bulk handling, spread manure, feeding, monitoring the animals for signs of illness, assist in artificial insemination, transporting stock to farms or processing plants, shearing and drenching livestock, sterilizing machines, and collecting, grading and packaging produce, and assisting with calving
Small animal farming (e.g., poultry, rabbit, fish, bee)	Raising and breeding of small animals such as poultry, rabbits, fish, frogs, bees, insects, minks, beavers, or snails	Feeding, breeding, disease prevention, assist in artificial insemination, gathering eggs, cleaning rooms and equipment/facility maintenance
Training, dressage, stud farming	Activity including raising and breeding of domestic horses as well as training and dressage of domestic equines	Feeding, breeding, assist in artificial insemination, disease prevention, teaching, maintenance of the property, and tourism
Shellfish farming (e.g., oyster farming, scallop aquaculture)	Cultivation and harvest of aquatic invertebrates, such as oysters, clams, and mussels	Breeding, rearing, harvesting, using a rack, processing, and shipment
Cooperatives (e.g., provision of supplies)	Employees of agricultural cooperatives (e.g., provision of supplies, slaughterhouse)	Animal handling, tool handling, vehicle driving
Gardener, park ranger	Persons entrusted with protecting and preserving lands	Law enforcement, education, firefighting, maintenance, administration
Gamekeeper, fishery officer	Person who manages an area of countryside (e.g., areas of woodland, moorland, waterway or farmland) to make sure that there is enough game for hunting, or fish for fishing, and acts as guide to those pursuing them	Gamekeeping, maintenance, administration
Salt works/salt evaporation pond	Production of salt from sea water	Harvesting, and filtering
Wood production (e.g., lopping)	Harvesting of round wood (trunk and branch wood) from coniferous (softwood) and non-coniferous (hardwood) trees	Harvesting, lopping, and transport
Stationary sawmill (e.g., edging, trimming, decking, debarking)	Cutting logs into lumber using large bandsaw blades	Edging, trimming, decking, debarking, use of machines
Agricultural work companies (e.g., pesticide applications, harvest reaping)	Companies that assist or perform farm works such as harvest reaping	Harvest reaping, harrowing, sowing seeds, pesticide application, tilling, and clearing
Gardening and landscaping companies	Companies that perform gardening and landscaping work	Gardening, landscaping, chainsaw and skidder operation, climbing on trees, monitoring and controlling pests and diseases
Administrative jobs	Employees of banks and federations	Administrative tasks
Construction rural craftsperson (e.g., mason, electrician, painter)	Employees performing construction work in rural areas, including jobs such as mason, electrician, painter	Fixing agricultural machinery, painting, welding, and hammering
Unspecified rural craftsperson (e.g., cooper, saddler, blacksmith)	Employees performing hand-crafted work in rural areas, including jobs such as cooper, saddler, blacksmith	Hammering, welding

Table S3: Number of farmers with a LTI, ODC or ATC code of interest

A. Number of PD farmers with a PD drug reimbursement based on the drug name

ATC code	Drug name	Indication*	Number of FMs No. (%)	Number of farmworkers No. (%)
N04	Anti-parkinson drugs solely used for PD	PD only (cf. below)	5354 (1.26)	2627 (0.48)
N04BA02	Levodopa and decarboxylase inhibitor	PD only	3083 (0.73)	1170 (0.21)
N04BC08	Piribedil	PD only	1747 (0.41)	940 (0.17)
N04BC04	Ropinirole	PD only	945 (0.22)	653 (0.12)
N04BD02	Rasagiline	PD only	939 (0.22)	600 (0.11)
N04BA03	Levodopa, decarboxylase inhibitor and COMT inhibitor	PD only	637 (0.15)	298 (0.05)
N04BX02	Entacapone	PD only	77 (0.02)	41 (7.5e-03)
N04BC07	Apomorphine	PD only	66 (0.02)	29 (5.3e-03)
N04BD01	Selegiline	PD only	64 (0.02)	30 (5.5e-03)
N04BC01	Bromocriptine	PD only	38 (8.9e-03)	32 (5.8e-03)
N04BX01	Tolcapone	PD only	3 (7.1e-04)	2 (3.7e-04)
N04BA01	Levodopa	PD only	0	0
N04BC02	Pergolide	PD only	0	0

Abbreviations: COMT: catechol-O-methyltransferase, FM: farm manager, No: number of, PD: Parkinson's disease.

*According to the Vidal (https://www.vidal.fr/), Thesorimed (https://theso.prod-un.thesorimed.org/monographie) and BCB databases (https://www.bcb.fr/v2/app/recherche.jsp).

B. Number of PD farmers with a LTI or ODC declaration based on the ICD-10 code

Declaration type	ICD-10 code	Definition	Number of FMs No. (%)	Number of farmworkers No. (%)
LTI	G20	Parkinson's disease	2331 (0.55)	1084 (0.20)
LTI	G21	Secondary parkinsonism	72 (0.02)	35 (6.4e-03)
LTI	F02	Dementia in other diseases classified elsewhere	10 (2.4e-03)	5 (9.2e-04)
ODC	/	Work-related Parkinson's disease	102 (0.02)	39 (7.2e-03)

Abbreviations: ICD-10: 10th revision of the International Statistical Classification of Diseases and Related Health Problems, FM: farm manager, No: number of, ODCs: occupational diseases covered under workers' compensation statutes, PD: Parkinson's disease.

Table S4: List of potential covariates considered in the analyses

Dependent variable	Modality	Data type	Data source	Population
Disease status	2 categories: being diagnosed with Parkinson's disease or not	sociodemographic	LTD and ODC declarations, drug prescriptions	All
**Independent variables – potential covariates selected based on VIF**				
Sex[a]	2 categories: female or male	sociodemographic	social security contributions	All
Age	Continuous			
Smoking status	2 categories: yes or no		AGRICAN	All
Alcohol consumption	2 categories: yes or no			
Family status	2 categories: single or as a couple		social security contributions	FMs
Partner work status	2 categories: partner not helping FM or partner helping FM with farming tasks			
Number of years of experience	continuous	occupational	social security contributions	All
Median farm surface	continuous			FMs
Number of farms	2 categories: 1 farm or > 1 farm			
Farm/Work location	96 categories: administrative departments			All
Farm type (farm clustering)	2 categories: individual farm or farm with work partners			FMs
Number of years involved in a specific farming activity (n=26)	continuous			
Median number of associates	continuous			
Median number of employees	continuous			
Secondary activity	2 categories: yes or no			
Number of helping family members	2 categories: 0 or ≥ 1			
Work status	2 categories: working as a FM or as a solidarity contributor[b]			
Seasonal work	2 categories: yes or no			Farmworkers
Having a permanent job	2 categories: yes or no			
Being a middle manager or senior executive	2 categories: yes or no			
Contract income	continuous			
Percentage of partial time	continuous			
Number of years involved in a specific farming activity (n=22)	continuous			
Company size	5 categories: <20, 20-49, 50-149, 150-299, and >299 workers			
Number of pre-existing medical comorbidities	continuous	medical	LTD declarations	All
Pre-existing disability	2 categories: yes or no		Insurance claims	FMs
Previous LTD declaration	28 categories: individuals granted with a LTD[c]		LTD declarations	All
COPD (≥ 1 declaration (ICD-10 codes): J42-J44)	2 categories: yes or no			
Abnormalities of gait and mobility (≥ 1 declaration (ICD-10 code): R26)				
Tremor (≥ 1 declaration (ICD-10 codes): G25, R25)				
Anxiety (≥ 1 declaration (ICD-10 code): F41)				
Bipolar disorder (≥ 1 declaration (ICD-10 code): F31)				
Cognitive impairment (≥ 1 declaration (ICD-10 code): U51)				
Depression (≥ 1 declaration (ICD-10 codes): F32, F33)				
Long-term psychiatric conditions (≥ 1 declaration LTD #23)				
Crohn's disease (≥ 1 declaration (ICD-10 code): K50)				
Inflammatory bowel disease (≥ 1 declaration (ICD-10 codes): K50, K51)				
Ulcerative colitis (≥ 1 declaration (ICD-10 code): K51)				
Asthma (≥ 1 declaration (ICD-10 codes): J45)				
Psychosis (≥ 1 declaration (ICD-10 codes): F28, F29)				
Hepatitis (≥ 1 declaration (ICD-10 codes): B15-B19)				
CNS tumor (≥ 1 declaration (ICD-10 codes): C70-C72, D32, D33, D42, D43)				
Breast cancer (≥ 1 declaration (ICD-10 codes): C50, D24)				
Skin cancer (≥ 1 declaration (ICD-10 codes): C43, C44, D23)				
Colon cancer (≥ 1 declaration (ICD-10 code): C18)				
Cancer (≥ 1 declaration (ICD-10 codes): C00-C97)				
Anemia (≥ 1 declaration (ICD-10 codes): D63, D64)				
Type 2 diabetes (≥ 1 declaration (ICD-10 codes): E11)				
Dyslipidemia (≥ 1 declaration (ICD-10 codes): E78)				
Alcohol related disorder (≥ 1 declaration (ICD-10 codes): F10)				
Sleep disorder (≥ 1 declaration (ICD-10 codes): F51, G47)				
Cataract (≥ 1 declaration (ICD-10 codes): H25-H28)				
Glaucoma (≥ 1 declaration (ICD-10 codes): H40)				
Hearing loss (≥ 1 declaration (ICD-10 codes): H90, H91)				
Hypertension (≥ 1 declaration (ICD-10 codes): I10)				
Cerebrovascular disease (≥ 1 declaration (ICD-10 codes): I60-I69)				
Ischemic heart disease (≥ 1 declaration (ICD-10 codes): I20-I25)				
Arthrosclerosis (≥ 1 declaration (ICD-10 codes): I70)				
Multiple sclerosis (≥ 1 declaration (ICD-10 code): G35)				
Gout (≥ 1 declaration (ICD-10 codes): M10)				
Antiherpetic drugs (ATC codes: J05AB01-15, J05AD01-2, S01AD01-7, S01AD09, D06BB02, D06BB03, D06BB08, D06BB11)	continuous (number of drug reimbursements)		Drug reimbursements	All
Antihypertensive (ATC codes: C02, C03, C07, C08, C09)				
Antiplatelet drugs (ATC codes: B01AC)				
Immunosuppressant or immunomodulator (ATC codes: L03, L04)				
Antidepressant (ATC codes: N06A)				
Anxiolytics (ATC codes: N05)				
Aspirin (ATC codes: B01AC06, N02BA01, N02BA51)				

Ibuprofen (ATC codes: C01EB16, G02CC01, M01AE01, M01AE51, M02AA13, N02AJ19, R02AX02)				
Acetaminophen (ATC code: N02BE01)				
Non-aspirin NSAIDs (ATC codes: M01A)				
Selective beta-2-adrenoreceptor agonists (ATC codes: R03CC)				
Sulfonamides (ATC codes: C03CA)				
Calcium channel blocker (ATC codes: C08)				
Adrenergic in combination with anticholinergics (ATC codes: R03AL)				
Preparations inhibiting uric acid production (ATC codes: M04AA)				
Thiazolidinedione (ATC codes: A10BG)				
Insulins and analogues (ATC codes: A10AD)				
Muscle relaxant use (ATC codes: M03C)				
Oral contraceptives (ATC codes: G03A)				
Postmenopausal estrogen drugs (ATC codes: G03FA, G03FB)				
HMG CoA reductase inhibitors (statin) (ATC codes: C10AA)				
Hyperthyroidism (ATC codes: H03BA, H03BB)				
Hypothyroidism (ATC codes: H03AA)				
Overall antibiotics (ATC codes: J01, A02BD, A07A, P01AB)				
Tetracyclines antibiotics (ATC codes: J01AA)				
Penicillin antibiotics (ATC codes: J01CA, J01CE, J01CF)				
Other beta-lactam antibacterials (ATC codes: J01DB, J01DC, J01DD)				
Sulfonamides and trimethoprim antibiotics (ATC codes: J01EA, J01EB, J01EE)				
Macrolides and lincosamides antibiotics (ATC codes: J01FA, J01FF)				
Fluoroquinolones (ATC codes: J01MA)				
Other antibacterials (ATC codes: J01XC01, J01XE01, J01XX01, J01XX05, J01XX08, P01AB01-03, J01BA01, A07AA09)				
Antimycotics for systematic use (ATC codes: J02)				
Hypercholesterolemia (ATC codes: C10AA, C10AB, C10B; or ≥ 1 declaration (ICD-10 code): E78)	2 categories: yes or no		Drug reimbursements and LTD declarations	
Epilepsy (ATC codes: N03; or ≥ 1 declaration (ICD-10 code): G40)	2 categories: yes or no			
Head injury	2 categories: yes or no		WA declarations	

Note: ATC: Anatomical Therapeutic Chemical classification system, FM: farm manager, ICD-10: 10th revision of the International Statistical Classification of Diseases and Related Health Problems, LTD: long-term disease, LTD #: long-term disease category as defined by French laws (https://www.legifrance.gouv.fr/codes/section_lc/LEGITEXT000006073189/LEGISCTA000006156610/2020-01-01), ODC: occupational diseases covered under workers' compensation statutes, VIF: variance inflation factor, WA: workplace accident.

[a] The analysis was adjusted on sex only for "both sexes"; otherwise, the sex was used for subgroup analyses.

[b] A FM who farms on a small surface (<12.5ha) and/or who works less than 1200 hour/year.

[c] There are 30 LTD categories (e.g., cancer, long-term psychiatric conditions) defined by French laws (https://www.legifrance.gouv.fr/codes/section_lc/LEGITEXT000006073189/LEGISCTA000006156610/2020-01-01). LTD declarations for Parkinson's disease (LTD #16) and for Alzheimer's disease and other related dementias (LTD# 15) were not considered.

Table S5: Baseline characteristics (sociodemographic and farm characteristics) of the FM population

	FM without PD (n = 418460)	PD case (n = 6265)
**Main characteristics**	n (%)	n (%)
**Sex**		
Male	243681 (58.2)	3383 (54.0)
Female	174779 (41.8)	2882 (46.0)
**Age (years)**		
Mean (SD)	60.7 (9.03)	62.0 (8.18)
**Family status**		
Single	130734 (31.2)	1679 (26.8)
As a couple	287726 (68.8)	4586 (73.2)
**Number of years of experience**		
Mean (SD)	18.1 (9.86)	18.4 (9.39)
**Farm surface (expressed in hectares)**		
Mean (SD)	9.97 (3.29)	13.5 (3.89)
**Farm location (region)**		
Auvergne-Rhône-Alpes	46686 (11.2)	608 (9.69)
Bourgogne-Franche-Comté	24288 (5.80)	354 (5.65)
Bretagne	25339 (6.06)	322 (5.14)
Centre - Val de Loire	19600 (4.68)	322 (5.13)
Corse	1564 (0.37)	14 (0.22)
Grand Est	27562 (6.59)	476 (7.60)
Hauts-de-France	17299 (4.13)	377 (6.02)
Île-de-France	4692 (1.12)	60 (0.95)
Normandie	36211 (8.65)	549 (8.77)
Nouvelle-Aquitaine	73736 (17.6)	1153 (18.4)
Occitanie	69783 (16.7)	1028 (16.4)
Provence-Alpes-Côte d'Azur	45003 (10.8)	639 (10.2)
Pays de la Loire	26697 (6.38)	365 (5.83)
**Number of farms**		
1 farm	411446 (98.3)	6140 (98.0)
>1 farm	7014 (1.68)	122 (1.95)
**Partner work status**		
Perform task to help farm manager	364378 (87.1)	5369 (85.7)
Do not perform task to help farm manager	54082 (12.9)	896 (14.3)
**Number of associates**		
0	340646 (81.4)	5006 (79.9)
≥ 1	77814 (18.6)	1259 (20.1)
**Work diversification** (activity performed in parallel to a main declared activity)		
No	274203 (65.5)	5700 (91.0)
≥ 1 activity performed in in parallel to a main activity	144257 (34.5)	565 (9.02)
**Number of different yearly activities performed**		
Only one activity during the observation period	376614 (90)	5751 (91.8)
Two activities during the observation period	39335 (9.4)	495 (7.9)
At least three activities during the observation period	2511 (0.6)	19 (0.3)
**Lack of job security**		
Has never been unemployed during the observation period	411744 (98.4)	6194 (98.9)
Has been unemployed during the observation period	6716 (1.60)	71 (1.14)
**Median yearly insurance premium (euros)**		
Median (IQR)	6.0e+04 (6.0e+05)	1.7e+04 (2.6e+05)
**Employees**		
No employee	303384 (72.5)	5056 (80.7)
At least one employee	115076 (27.5)	1209 (19.3)
**Work status**		
Working as a farm manager	260449 (62.2)	4147 (66.2)
Working as a solidarity contributor[a]	158011 (37.8)	2118 (33.8)
**Pre-existing disability**		
Did not become disabled before the disease diagnosis or end of follow-up	411424 (98.3)	6058 (96.7)
Became disabled before the disease diagnosis or end of follow-up	7036 (1.68)	207 (3.30)
**Number of pre-existing comorbidities (long term illness)**		
Median (IQR)	0.13 (0.47)	0.16 (0.52)
**Retirement status**		
FMs who did not retire between 2002-2016	128883 (30.8)	652 (20.4)
FMs who retired between 2002-2016	289577 (69.2)	5613 (89.6)
FMs who retired between 2012-2016	40870 (9.62)	4997 (79.8)
**Number of years involved in a farming activity**: Mean (SD)		
Truck farming, floriculture/flower-growing	4.82 (3.25)	5.54 (3.93)
Fruit arboriculture	5.46 (3.56)	5.96 (4.24)
Garden center/tree nursery	5.82 (3.84)	5.55 (3.64)
Crop farming (e.g., wheat, corn, and industrial grower)	4.95 (3.45)	5.54 (4.30)
Viticulture	5.41 (3.46)	6.50 (4.43)
Sylviculture/forestry (e.g., thinning, pruning)	5.70 (4.01)	6.22 (4.06)
Unspecified specialized farming (e.g., herbs, mushrooms)	5.62 (3.99)	5.29 (3.52)
Dairy farming	4.64 (2.95)	5.37 (3.57)
Cow farming	5.34 (3.26)	6.51 (4.16)
Mixed cattle farming	5.38 (3.35)	6.32 (3.90)
Ovine and caprine farming	6.13 (4.11)	5.21 (3.43)

Pig farming	5.60 (3.44)	4.96 (3.03)
Stud farming	6.20 (3.95)	5.23 (3.78)
Unspecified large animal farming (e.g., ostrich, llama)	5.22 (3.58)	6.01 (4.01)
Poultry and rabbit farming	5.25 (3.53)	4.73 (2.84)
Unspecified small animal farming (e.g., frogs, snails, bees)	5.56 (4.26)	6.08 (3.33)
Training, dressage, riding clubs	6.09 (4.34)	4.40 (2.44)
Shellfish farming (e.g., oyster farming, scallop aquaculture)	5.12 (4.07)	4.67 (1.63)
Unspecified and mixed farming (e.g., polyculture, mixed farming, diversified farming)	5.31 (3.25)	6.01 (4.07)
Salt works/salt evaporation pond	6.10 (4.21)	3.00 (0.00)
Wood production (e.g., lopping)	5.43 (3.84)	4.77 (3.56)
Stationary sawmill (e.g., edging, trimming, decking, debarking)	6.31 (3.74)	2.50 (2.12)
Agricultural work companies (e.g., pesticide applications, harvest reaping)	5.76 (4.21)	5.14 (3.42)
Gardening, landscaping and reforestation companies	5.42 (3.78)	4.69 (2.92)
Company representative/authorized representative	3.78 (2.88)	3.23 (2.28)
Rural craftsperson (e.g., mason, mechanics)	4.93 (3.11)	5.75 (1.71)

Note: FM: farm manager, IQR: interquartile range, PD: Parkinson’s disease, SD: arithmetic standard deviation.
*FMs who farm on a small surface (<12.5 ha) or who work less than 1200 h/year.

Table S6: Baseline characteristics (medical covariates) of both farming populations

Factor	FM without PD n (%) [norm%[a]] median (IQR)[b]	FM with PD n (%) [norm%[a]] median (IQR)[b]	Employee without PD n (%) [norm%[a]] median (IQR)[b]	Employee with PD n (%) [norm%[a]] median (IQR)[b]
Abnormalities of gait and mobility	5 (1.2e-03) [100]	0 (0) [0]	2 (3.7e-04) [100]	0 (0) [0]
Acetaminophen	3.26 (5.71)	2 (4.23)	1.52 (3.94)	2.25 (4.38)
Adrenergic in combination with anticholinergics	0.03 (0.55)	8.2e-03 (0.18)	2.92 (3.11)	2.74 (2.33)
Alcohol related disorder	244 (0.06) [36.4]	6 (0.10) [63.6]	352 (0.07) [8.10]	21 (0.74) [91.9]
Anemia	6 (1.4e-03) [100]	0 (0) [0]	2 (3.7e-04) [100]	0 (0) [0]
Antidepressant	0.90 (4.10)	1.17 (3.99)	0.41 (2.74)	1.43 (4.32)
Antiherpetic drugs	0.07 (0.61)	0.03 (0.41)	0.04 (0.47)	0.06 (0.50)
Antihypertensive	5.24 (8.85)	3.08 (6.16)	2.20 (5.98)	3.15 (6.22)
Antimycotics for systematic use	0.03 (0.33)	0.02 (0.20)	0.02 (0.25)	0.03 (0.28)
Antiplatelet drugs	1.75 (5.44)	1.12 (3.87)	0.76 (3.60)	1.23 (4.17)
Anxiety	326 (0.08) [18.2]	22 (0.35) [81.8]	247 (0.05) [9.76]	12 (0.42) [90.2]
Anxiolytics	1.82 (5.89)	1.90 (5.47)	0.87 (4.10)	2.53 (6.18)
Arthrosclerosis	4105 (0.98) [48.7]	65 (1.03) [51.3]	3369 (0.62) [34.9]	33 (1.16) [65.1]
Aspirin	1.60 (5.10)	1.00 (3.62)	0.69 (3.33)	1.13 (3.92)
Asthma	1011 (0.24) [44.1]	19 (0.31) [55.9]	682 (0.13) [28.5]	9 (0.32) [71.5]
Bipolar disorder	807 (0.19) [9.51]	115 (1.84) [90.5]	519 (0.10) [4.42]	59 (2.07) [95.6]
Breast cancer	5113 (1.22) [48.5]	81 (1.30) [51.5]	3243 (0.60) [34.8]	32 (1.12) [65.2]
Calcium channel blocker	1.18 (4.40)	0.67 (2.88)	0.47 (2.72)	0.73 (3.22)
Cataract	4 (9.6e-04) [100]	0 (0) [0]	1 (1.9e-04) [100]	0 (0) [0]
Cerebrovascular disease	6515 (1.56) [46.5]	112 (1.79) [53.5]	3337 (0.62) [21.2]	65 (2.28) [78.8]
CNS tumor	538 (0.13) [44.5]	10 (0.16) [55.5]	349 (0.06) [37.9]	3 (0.11) [62.1]
Cognitive impairment	0 (0) [0]	0 (0) [0]	0 (0) [0]	0 (0) [0]
Colon cancer	2310 (0.55) [58.4]	24 (0.39) [41.6]	1316 (0.24) [33.1]	14 (0.49) [66.9]
COPD	1480 (0.35) [56.1]	18 (0.28) [43.9]	1016 (0.19) [32.7]	11 (0.39) [67.3]
Crohn's disease	172 (0.04) [48.5]	3 (0.04) [51.5]	181 (0.03) [100]	0 (0) [0]
Depression	2316 (0.55) [20.2]	137 (2.18) [79.8]	1937 (0.36) [10.5]	87 (3.05) [89.5]
Epilepsy	580 (0.14) [1.69]	504 (8.04) [98.3]	403 (0.07) [0.61]	345 (12.1) [99.4]
Fluoroquinolones	0.18 (0.72)	0.11 (0.50)	0.09 (0.49)	0.14 (0.57)
Glaucoma	97 (0.02) [61.4]	1 (0.01) [38.6]	31 (5.7e-03) [100]	0 (0) [0]
Gout	9 (2.2e-03) [100]	0 (0) [0]	4 (7.4e-04) [100]	0 (0) [0]
Head injury	1755 (0.42) [56.7]	20 (0.32) [43.3]	0 (0) [0]	8 (0.28) [100]
Hearing loss	21 (5.0e-03) [100]	0 (0) [0]	15 (2.8e-03) [100]	0 (0) [0]
Hepatitis	227 (0.05) [22.3]	12 (0.19) [77.7]	335 (0.06) [37]	3 (0.11) [63]
HMG CoA reductase inhibitors (statin)	1.93 (5.19)	1.21 (3.59)	0.96 (3.67)	1.54 (4.26)
Hypercholesterolemia	16 (3.8e-03) [0.02]	1165 (18.6) [100]	24 (4.4e-03) [0.02]	644 (22.6) [100]
Hypertension	10645 (2.54) [33.6]	315 (5.03) [66.4]	5232 (0.97) [21.9]	98 (3.44) [78.1]
Hyperthyroidism	466 (0.11) [60.5]	4 (0.07) [39.5]	16 (3.0e-03) [100]	0 (0) [0]
Hypothyroidism	19577 (4.68) [47.7]	321 (5.13) [52.3]	1641 (0.30) [38.1]	14 (0.49) [61.9]
Ibuprofen	0.30 (1.10)	0.15 (0.78)	0.19 (0.88)	0.22 (0.78)
Immunosuppressant or immunomodulator	0.09 (1.09)	0.04 (0.59)	0.05 (0.78)	0.06 (0.68)
Inflammatory bowel disease	402 (0.10) [48.5]	6 (0.10) [51.5]	795 (0.15) [37.4]	7 (0.25) [62.6]
Insulins and analogues	0.04 (0.84)	0.03 (0.53)	0.02 (0.56)	0.05 (0.86)
Ischemic heart disease	15843 (3.79) [48.4]	253 (4.03) [51.6]	10248 (1.89) [30.5]	123 (4.32) [69.5]
Long-term psychiatric conditions	469 (0.11) [8.98]	71 (1.14) [91]	853 (0.16) [4.74]	90 (3.16) [95.3]
Macrolides and lincosamides antibiotics	0.17 (0.65)	0.09 (0.40)	0.09 (0.46)	0.12 (0.51)
Muscle relaxant use	1.3e-03 (0.14)	8.3e-04 (0.07)	5.5e-04 (0.10)	8.0e-03 (0.32)
Non-aspirin NSAIDs	1.55 (3.60)	1.01 (2.75)	0.86 (2.63)	1.23 (2.85)
Oral contraceptives	2.1e-03 (0.13)	0 (0)	2.1e-03 (0.11)	4.3e-03 (0.20)
Other antibacterials	0.12 (0.73)	0.07 (0.44)	0.06 (0.45)	0.08 (0.40)
Other beta-lactam antibacterials	0.20 (0.74)	0.12 (0.51)	0.09 (0.48)	0.12 (0.50)
Overall antibiotics	1.36 (2.71)	0.74 (1.79)	0.70 (1.96)	0.89 (1.92)
Penicillin antibiotics	0.26 (0.78)	0.13 (0.49)	0.14 (0.59)	0.17 (0.54)
Postmenopausal estrogen drugs	0.09 (0.90)	0.05 (0.54)	0.07 (0.76)	0.10 (0.85)
Preparations inhibiting uric acid production	0.51 (2.88)	0.29 (1.90)	0.24 (1.94)	0.33 (2.08)
Psychosis	245 (0.06) [6.93]	50 (0.79) [93.1]	174 (0.03) [2.78]	32 (1.12) [97.2]
Selective beta-2-adrenoreceptor agonists	0.01 (0.48)	0.01 (0.33)	4.9e-03 (0.28)	0.02 (0.43)
Skin cancer	1429 (0.34) [51.6]	20 (0.32) [48.4]	810 (0.15) [24.7]	13 (0.46) [75.3]
Sleep disorder	363 (0.09) [37.3]	9 (0.15) [62.7]	202 (0.04) [51.5]	1 (0.04) [48.5]
Sulfonamides	0.77 (3.81)	0.44 (2.45)	0.18 (1.84)	0.30 (2.12)
Sulfonamides and trimethoprim antibiotics	0.04 (0.41)	0.02 (0.26)	0.02 (0.27)	0.02 (0.24)
Tetracyclines antibiotics	0.03 (0.32)	0.01 (0.16)	0.02 (0.27)	0.02 (0.24)
Thiazolidinedione	0 (0)	0 (0)	0 (0)	0 (0)
Tremor	9 (2.2e-03) [12.9]	1 (0.01) [87.1]	5 (9.2e-04) [100]	0 (0) [0]
Type 2 diabetes	27177 (6.49) [46.4]	471 (7.52) [53.6]	19939 (3.68) [27.7]	274 (9.62) [72.3]
Ulcerative colitis	304 (0.07) [62.4]	3 (0.04) [37.6]	331 (0.06) [36.7]	3 (0.11) [63.3]

Note: ATC: Anatomical Therapeutic Chemical classification system, FM: farm manager, ICD-10: 10th revision of the International Statistical Classification of Diseases and Related Health Problems, LTD: long-term disease, LTD #: long-term disease category as defined by French laws (https://www.legifrance.gouv.fr/codes/section_lc/LEGITEXT000006073189/LEGISCTA000006156610/2020-01-01).

[a] To allow for a direct comparison of the percentage of individuals in each category (either diagnosed or not diagnosed with PD), the relative contribution of each category to the total population (e.g., FM or employee) across both categories was calculated by normalizing the percentage of each category to their combined total. For instance, among FMs, there were 51.2% of FMs not diagnosed with PD and 39.2 diagnosed with PD who used

acetaminophen, resulting in a normalized percentage of 56.7 (51.2/51.2+39.2) and 43.3 (39.2/51.2+39.2), respectively.

[b] Medication use is provided as the median number of reimbursements prior to diagnosis or censoring.

Table S7: Baseline characteristics (sociodemographic and farm characteristics) of the farmworker population

	Farmworker without PD (n = 541940)	PD case (n = 2848)
**Main characteristics**	n (%)	n (%)
**Sex**		
Female	205066 (37.8)	1015 (35.6)
Male	336874 (62.2)	1833 (64.4)
**Age (years)**		
Mean (SD)	56.5 (4.97)	56.8 (4.88)
**Work status**		
Being a seasonal worker	269732 (49.8)	573 (20.1)
Not being a seasonal worker	272208 (50.2)	2275 (79.9)
**Job type**		
Not having a permanent job	390471 (72.1)	1068 (37.5)
Having a permanent job	151469 (27.9)	1780 (62.5)
**Work type**		
Being an employee	509143 (93.9)	2433 (85.4)
Being a middle manager or senior executive	32797 (6.05)	415 (14.6)
**Contract income (euros)**		
Median (IQR)	7140 (1.6e+04)	1.5e+04 (1.9e+04)
**Number of years of experience**		
Mean (SD)	6.13 (7.27)	10.8 (8.63)
**Percentage of partial time**		
Median (IQR)	0.04 (0.15)	0.06 (0.19)
**Work location (region)**		
Auvergne-Rhône-Alpes	56233 (10.4)	241 (8.46)
Bourgogne-Franche-Comté	40147 (7.41)	139 (4.88)
Bretagne	32451 (5.99)	219 (7.69)
Centre - Val de Loire	27192 (5.02)	168 (5.90)
Corse	2447 (0.45)	5 (0.18)
Grand Est	85172 (15.7)	283 (9.94)
Hauts-de-France	22670 (4.18)	170 (5.97)
Île-de-France	19086 (3.52)	170 (5.97)
Normandie	17726 (3.27)	119 (4.18)
Nouvelle-Aquitaine	89292 (16.5)	505 (17.7)
Occitanie	68446 (12.6)	393 (13.8)
Provence-Alpes-Côte d'Azur	64548 (11.9)	313 (11)
Pays de la Loire	42918 (7.92)	221 (7.76)
**Company size**		
Working in a company with fewer than 20 workers	468648 (86.5)	2308 (81)
Working in a company with between 20 and 49 workers	30354 (5.60)	188 (6.60)
Working in a company with between 50 and 149 workers	19055 (3.52)	143 (5.02)
Working in a company with between 150 and 299 workers	9457 (1.75)	79 (2.77)
Working in a company with over 299 workers	14426 (2.66)	130 (4.56)
**Number of pre-existing comorbidities (long term illness)**		
Median (IQR)	0.18 (0.50)	0.53 (0.78)
**Retirement status**		
Farmworkers who did not retire between 2002-2016	497057 (91.7)	2549 (89.5)
Farmworkers who retired between 2002-2016	44883 (8.28)	299 (10.5)
Farmworkers who retired between 2012-2016	40870 (7.54)	253 (8.88)
**Number of years involved in a farming activity**: Mean (SD)		
Unspecified farming (e.g., mixed farming, cereal farming, potato farming, pasture)	2.35 (3.25)	2.76 (2.98)
Viticulture	2.94 (3.75)	2.96 (3.14)
Vinification	2.95 (3.57)	2.16 (2.21)
Sylviculture/forestry (e.g., thinning, pruning)	2.80 (3.48)	2.90 (2.67)
Resin extraction	2.7e-03 (0)	0 (0)
Unspecified specialized crop farming (e.g., soy farming, fruit arboriculture, truck farming)	2.35 (3.25)	2.76 (2.98)
Mushroom farming	2.25 (2.91)	2.11 (2.05)
Large animal farming (e.g., cattle, ovine, pig)	1.90 (2.77)	1.70 (1.87)
Small animal farming (e.g., poultry, rabbit, fish, bee)	2.21 (3.06)	2.56 (2.61)
Training, dressage, stud farming	2.38 (3.24)	2.14 (2.10)
Shellfish farming (e.g., oyster farming, scallop aquaculture)	2.33 (3.33)	1.54 (1.85)
Cooperatives (e.g., provision of supplies)	3.59 (3.57)	3.65 (3.08)
Gardener, park ranger	3.30 (3.77)	3.35 (3.22)
Gamekeeper, fishery officer	3.06 (3.70)	2.50 (3.54)
Salt works/salt evaporation pond	2.79 (2.96)	3.76 (3.76)
Wood production (e.g., lopping)	2.27 (3.16)	3.01 (3.28)
Stationary sawmill (e.g., edging, trimming, decking, debarking)	2.94 (3.38)	2.53 (2.28)
Agricultural work companies (e.g., pesticide applications, harvest reaping)	1.87 (2.94)	1.80 (2.42)
Gardening and landscaping companies	2.37 (3.25)	3.18 (2.93)
Administrative jobs	2.92 (3.11)	2.74 (2.33)
Construction rural craftsperson (e.g., mason, electrician, painter)	2.09 (2.48)	0.25 (0)
Unspecified rural craftsperson (e.g., cooper, saddler, blacksmith)	2.69 (3.27)	2.43 (1.93)

Note: IQR: interquartile range, PD: Parkinson's disease, SD: arithmetic standard deviation.

Table S8: Best model hyperparameters and model tuning duration

Hyperparameters	Main analysis		SA1		SA2		SA3		SA4		SA5		SA6	
	FM	*FW*	*FM*	*FW*	*FM*	*FW*	*FM*	*FW*	*FM*	*FW*	*FM*	*FW*	*FM*	*FW*
Number of boosting rounds	100	100	100	100	100	100	100	100	100	100	100	100	65	98
Learning rate	0.1	0.1	0.1	0.05	0.1	0.1	0.1	0.1	0.1	0.1	0.1	0.1	0.05	0.05
Maximum tree depth	5	5	5	3	5	5	5	5	5	5	5	5	5	3
Minimum child weight	10	10	10	5	10	10	10	10	10	10	10	10	10	5
Subsample ratio	1	1	1	1	1	1	1	1	1	1	1	1	0.9	1
Column-sample ratio	0.9	0.9	0.9	0.9	0.9	0.9	0.9	0.9	0.9	0.9	0.9	0.9	1	0.9
Number of threads	2	2	2	2	2	2	2	2	2	2	2	2	2	2
Model tuning duration (min)	31.5	40.0	30.4	25.9	17.0	13.7	28.0	38.9	34.6	42.7	32.7	35.3	9.73	9.10

FM: farm manager, FW: farmworker, SA: sensitivity analysis.

SA1: application of an uniform 1-year lag to all predictors.

SA2: application of a 1-year lag to all health-related predictors and a 5-year lag to all other predictors.

SA3: not considering smoking and alcohol consumption.

SA4: not considering age and sex.

SA5: not considering age, sex, smoking and alcohol consumption.

SA6: baseline model including only age and sex.

Table S9: Mean absolute SHAP value of each model's feature, directionality of each feature's effect on PD risk, univariate association with Cox model, and comparison with the literature for the main analysis

Population	Feature/Predictor	mean |SHAP| (95% CI)	SHAP direction	HR [95% CI]	Cox direction	Cox (C-index)	Cox p-value	Adjusted Cox p-value	Literature	Reference
Farm manager	Acetaminophen	0.18 (0.01; 0.40)	promoting	1.06 [1.05; 1.07]	positive	0.65	2.5e-13	5.4e-13	conflicting results	[1,2]
Farm manager	Age	0.26 (0.02; 0.55)	promoting	14.3 [10.1; 20.4]	positive	0.63	3.4e-47	6.8e-47	risk factor	[4,5]
Farm manager	Agricultural work companies (e.g., pesticide applications, harvest reaping)	5.2e-03 (4.1e-03; 6.8e-03)	promoting	1.01 [0.93; 1.07]	uncertain	0.63	0.32	0.34	uncertain	[35]
Farm manager	Antidepressant	0.07 (0.03; 0.39)	promoting	1.01 [1.00; 1.02]	uncertain	0.63	0.06	0.08	risk factor	[6,7]
Farm manager	Anxiolytics	0.03 (4.7e-03; 0.10)	promoting	1.00 [0.99; 1.01]	uncertain	0.63	0.41	0.42	risk factor	[6,7]
Farm manager	Aspirin	0.06 (2.6e-03; 0.59)	promoting	1.04 [1.03; 1.05]	positive	0.64	5.8e-13	1.1e-12	risk factor	[9,12]
Farm manager	Asthma	2.3e-03 (7.7e-04; 1.5e-03)	promoting	1.45 [0.72; 2.90]	uncertain	0.63	0.52	0.52	risk factor	[13]
Farm manager	Being a male	4.5e-03 (2e-04; 0.02)	promoting	1.10 [1.02; 1.18]	positive	0.63	3.4e-7	5.8e-07	risk factor	[14]
Farm manager	Bipolar disorder	3.5-03 (1.8e-03; 3.7e-03)	promoting	11.6 [8.58; 15.6]	positive	0.64	3.9e-35	2.1e-34	risk factor	[12,15]
Farm manager	Calcium channel blocker	0.01 (2.2e-03; 0.14)	mitigating	0.95 [0.94; 0.97]	negative	0.64	5.1e-8	9.1e-08	protective factor	[9,12,15-18]
Farm manager	Cancer	2.3e-04 (2.8e-05; 9.3e-04)	mitigating	0.98 [0.83; 1.16]	uncertain	0.63	0.27	0.30	protective factor	[15]
Farm manager	Cow farming	0.16 (0.06; 1.03)	promoting	1.19 [1.11; 1.34]	positive	0.64	4.9e-17	1.1e-16	risk factor	[35]
Farm manager	Crop farming (e.g., wheat, corn, and industrial grower)	0.54 (0.11; 3.48)	promoting	1.57 [1.36; 1.79]	positive	0.69	4.5e-19	1.3e-18	risk factor	[35,36,38]
Farm manager	Dairy farming	0.13 (0.05; 0.67)	promoting	1.26 [1.13; 1.45]	positive	0.63	1.7e-18	4.6e-18	conflicting results	[35,39]
Farm manager	Depression	6.2e-03 (1.8e-03; 6.4e-03)	promoting	4.11 [3.08; 5.47]	positive	0.64	1.37e-29	4.9e-29	risk factor	[9,10,12,20,21]
Farm manager	Epilepsy	0.03 (0.01; 0.04)	promoting	35.4 [30.2; 41.5]	positive	0.66	4.3e-207	6.2e-206	risk factor	[22,29,30]
Farm manager	Fruit arboriculture	0.03 (0.02; 0.04)	promoting	1.03 [1.01; 1.07]	positive	0.63	8.4e-4	1.3e-03	risk factor	[35,36]
Farm manager	Gardening, landscaping and reforestation companies	2.4e-03 (1.1e-03; 5.9e-03)	mitigating	0.98 [0.94; 0.99]	negative	0.63	1.3e-3	1.9e-03	protective factor	[35]
Farm manager	HMG CoA reductase inhibitors (statin)	0.26 (0.10; 0.70)	mitigating	0.97 [0.96; 0.98]	negative	0.63	9.3e-7	1.5e-06	protective factor	[9,12,15,16,23]
Farm manager	Hypercholesterolemia	0.50 (0.23; 0.81)	promoting	84.6 [75.8; 94.4]	positive	0.70	2.6e-231	5.6e-230	conflicting results	[24,31-34]
Farm manager	Hypertension	0.01 (3.2e-03; 0.12)	promoting	1.64 [1.34; 2.01]	positive	0.63	1.8e-30	7.7e-30	risk factor	[10,16,25]
Farm manager	Ibuprofen	0.03 (6.8e-03; 0.12)	mitigating	0.72 [0.66; 0.79]	negative	0.64	2.2e-34	1.1e-33	protective factor	[9,12,16]
Farm manager	Long-term psychiatric conditions	3.0e-03 (1.7e-03; 3.5e-03)	promoting	5.99 [4.01; 8.97]	positive	0.64	5.2e-56	3.2e-55		
Farm manager	Macrolides and lincosamides antibiotics	1.6e-04 (3.9e-05; 8.8e-04)	mitigating	0.66 [0.58; 0.74]	negative	0.63	2.2e-60	1.6e-59	protective factor	[28]
Farm manager	Median farm surface (hectares)	0.11 (4.0e-03; 0.61)	promoting	1.24 [1.03; 1.63]	positive	0.64	3.3e-9	6.2e-09		
Farm manager	Mixed cattle farming	0.03 (0.02; 0.03)	promoting	1.38 [1.16; 1.92]	positive	0.63	3.4e-18	8.6e-18	risk factor	[35-37]
Farm manager	Non-aspirin NSAIDs	0.03 (3.2e-03; 0.07)	mitigating	0.93 [0.91; 0.95]	negative	0.64	3.5e-13	7.2e-13	protective factor	[9,23,26]
Farm manager	Number of years of experience	0.19 (0.01; 0.53)	mitigating	0.92 [0.85; 0.98]	negative	0.65	3.4e-6	5.4e-06		
Farm manager	Overall antibiotics	0.19 (0.06; 0.41)	mitigating	0.87 [0.78; 0.91]	negative	0.63	4.8e-25	1.6e-24		
Farm manager	Ovine and caprine farming	0.05 (0.02; 0.06)	mitigating	0.97 [0.95; 0.99]	negative	0.63	4.2e-3	6.0e-03	uncertain	[35,37]
Farm manager	Pig farming	4.7e-03 (3.5e-03; 5.3e-03)	promoting	1.00 [0.91; 1.27]	uncertain	0.63	0.36	0.38	conflicting results	[35,37]
Farm manager	Poultry and rabbit farming	8.0e-03 (5.0e-03; 9.9e-03)	mitigating	0.98 [0.81; 1.09]	uncertain	0.63	0.22	0.25	conflicting results	[35,37]
Farm manager	Smoking	0.18 (0.04; 1.34)	mitigating	0.08 [0.05; 0.12]	negative	0.66	4.7e-315	1.1e-308	protective factor	[9,10,12,15-17,27,31]
Farm manager	Stud farming	0.02 (0.01; 0.03)	mitigating	0.96 [0.91; 0.99]	negative	0.64	0.04	0.05	conflicting results	[35-37]
Farm manager	Sulfonamides	5.1e-03 (1.4e-03; 0.04)	mitigating	0.91 [0.78; 1.06]	uncertain	0.63	0.16	0.20	protective factor	[3]
Farm manager	Sulfonamides and trimethoprim antibiotics	2.4e-04 (1.2e-04; 1.5e-03)	mitigating	0.77 [0.69; 0.87]	negative	0.63	1.2e-21	3.7e-21		
Farm manager	Training, dressage, riding clubs	2.4e-03 (1.9e-03; 3.1e-03)	mitigating	0.95 [0.90; 0.98]	negative	0.63	0.01	0.01	protective factor	[35]
Farm manager	Truck farming, floriculture/flower-growing	0.04 (0.02; 0.05)	promoting	1.01 [0.94; 1.15]	uncertain	0.63	0.19	0.22	risk factor	[35]
Farm manager	Type 2 diabetes	3.6e-03 (1.0e-04; 0.02)	promoting	1.41 [1.22; 1.64]	positive	0.63	3.7e-30	1.4e-29	risk factor	[10,12,15,16,25]
Farm manager	Unspecified and mixed farming (e.g., polyculture, mixed farming, diversified farming)	0.17 (0.05; 1.06)	promoting	1.05 [0.88; 1.32]	uncertain	0.64	0.18	0.22	uncertain	[35]
Farm manager	Viticulture	0.19 (0.07; 0.87)	promoting	1.08 [1.02; 1.29]	positive	0.65	5.1e-3	7.1e-03	conflicting results	[35,36,40]
Farm manager	Work diversification	0.45 (0.05; 1.19)	mitigating	0.20 [0.17; 0.23]	negative	0.72	5.1e-206	5.5e-205		
Farm manager	Working as a solidarity contributor	0.02 (6.6e-04; 0.09)	mitigating	0.64 [0.57; 0.70]	negative	0.65	2.9e-63	2.5e-62		
Farmworker	Acetaminophen	0.09 (4.1e-03; 0.34)	promoting	1.02 [1.00; 1.03]	positive	0.62	0.05	0.09	conflicting results	[1,2]
Farmworker	Age	0.45 (0.04; 0.97)	promoting	9.47 [3.83; 23.4]	positive	0.62	4.7e-09	1.0e-08	risk factor	[4,5]
Farmworker	Agricultural work companies (e.g., pesticide applications, harvest reaping)	0.02 (0.01; 0.03)	promoting	1.01 [0.98; 1.05]	uncertain	0.62	0.08	0.12		
Farmworker	Antidepressant	0.08 (0.03; 0.60)	promoting	1.05 [1.04; 1.06]	positive	0.64	1.1e-17	4.0e-17	risk factor	[6,7]

Farmworker	Antiherpetic drugs	2.4e-04 (1.5e-04; 3.0e-04)	mitigating	1.03 [0.93; 1.14]	uncertain	0.62	0.12	0.16	protective factor	[8]
Farmworker	Anxiolytics	0.11 (0.03; 0.30)	promoting	1.04 [1.03; 1.05]	positive	0.64	9.1e-20	4.0e-19	risk factor	[6,7]
Farmworker	Aspirin	9.1e-03 (2.9e-04; 0.04)	promoting	1.01 [1.00; 1.03]	uncertain	0.62	0.06	0.10	risk factor	[9,12]
Farmworker	Being a male	6.7e-03 (1.6e-03; 0.04)	promoting	1.09 [0.95; 1.25]	uncertain	0.62	0.09	0.12	risk factor	[14]
Farmworker	Being a seasonal worker	0.35 (0.07; 0.80)	mitigating	0.21 [0.18; 0.25]	negative	0.73	2.8e-94	4.1e-93		
Farmworker	Bipolar disorder	5.3e-03 (2.7e-03; 7.3e-03)	promoting	24.6 [15.4; 39.3]	positive	0.63	1.4e-25	8.8e-25	risk factor	[12,15]
Farmworker	Calcium channel blocker	9.7e-03 (4.2e-03; 0.01)	mitigating	1.01 [1.00; 1.03]	uncertain	0.62	0.07	0.11	protective factor	[9,12,15-18]
Farmworker	Cancer	4.5e-03 (9.9e-04; 0.04)	promoting	1.64 [1.27; 2.11]	positive	0.62	4.5e-11	1.2e-10	protective factor	[15]
Farmworker	Depression	5.5e-03 (2.9e-03; 5.6e-03)	promoting	7.38 [4.99; 10.9]	positive	0.63	8.3e-21	4.1e-20	risk factor	[9,10,12,20,21]
Farmworker	Epilepsy	0.01(5.3e-04; 0.02)	promoting	82.1 [66.6; 101]	positive	0.67	1.4e-154	3.1e-153	risk factor	[22,29,30]
Farmworker	Fluoroquinolones	8.2e-04 (2.0e-04; 3.9e-03)	promoting	1.08 [0.99; 1.18]	uncertain	0.62	0.06	0.10		
Farmworker	Gardener, park ranger	0.04 (0.02; 0.05)	mitigating	0.97 [0.96; 1.00]	negative	0.62	0.05	0.09		
Farmworker	Gardening and landscaping companies	0.04 (0.02; 0.04)	mitigating	0.98 [0.94; 1.13]	uncertain	0.62	0.45	0.45		
Farmworker	HMG CoA reductase inhibitors (statin)	0.14 (0.05; 0.77)	mitigating	0.98 [0.97; 0.99]	negative	0.62	4.7e-03	9.4e-03	protective factor	[9,12,15,16,23]
Farmworker	Hypercholesterolemia	0.20 (0.17; 0.29)	promoting	218 [187; 256]	positive	0.70	2.8e-232	1.2e-230	conflicting results	[24,31-34]
Farmworker	Hypertension	6.4e-03 (2.6e-03; 9.3e-03)	promoting	3.41 [2.46; 4.71]	positive	0.63	1.4e-16	4.4e-16	risk factor	[10,16,25]
Farmworker	Ibuprofen	0.04 (0.01; 0.17)	mitigating	0.89 [0.81; 0.98]	negative	0.62	7.1e-10	1.7e-09	protective factor	[9,12,16]
Farmworker	Ischemic heart disease	3.9e-03 (4.9e-04; 4.9e-03)	promoting	1.62 [1.16; 2.25]	positive	0.62	7.5e-10	1.7e-09	risk factor	[15]
Farmworker	Large animal farming (e.g., cattle, ovine, pig)	5.3e-03 (4.3e-03; 6.5e-03)	promoting	1.01 [0.98; 1.03]	uncertain	0.62	0.09	0.12		
Farmworker	Long-term psychiatric conditions	0.01 (6.4e-03; 0.01)	promoting	17.8 [12.3; 25.8]	positive	0.63	3.5e-32	2.6e-31		
Farmworker	Macrolides and lincosamides antibiotics	6.7e-03 (1.1e-03; 0.02)	promoting	1.06 [0.95; 1.18]	uncertain	0.62	0.22	0.24	protective factor	[28]
Farmworker	Non-aspirin NSAIDs	0.05 (4.2e-03; 0.13)	mitigating	1.00 [0.98; 1.03]	uncertain	0.62	0.14	0.18	protective factor	[9,23,26]
Farmworker	Number of years of experience	0.87 (0.03; 2.57)	mitigating	0.42 [0.19; 0.71]	negative	0.62	8.0e-05	1.21e-04		
Farmworker	Other beta-lactam antibacterials	1.9e-03 (9.8e-04; 0.01)	promoting	1.06 [0.95; 1.17]	uncertain	0.62	0.15	0.18		
Farmworker	Overall antibiotics	0.14 (0.06; 0.36)	mitigating	1.01 [0.98; 1.04]	uncertain	0.62	0.10	0.13		
Farmworker	Penicillin antibiotics	1.3e-03 (3.0e-04; 0.01)	promoting	0.96 [0.86; 1.06]	uncertain	0.62	0.17	0.20		
Farmworker	Percentage of part-time job	0.02 (3.0e-03; 0.17)	mitigating	0.54 [0.18; 0.85]	negative	0.62	2.9e-13	8.0e-13		
Farmworker	Shellfish farming (e.g., oyster farming, scallop aquaculture)	0.01 (0.01; 0.02)	mitigating	0.99 [0.91; 1.06]	uncertain	0.62	0.34	0.35		
Farmworker	Small animal farming (e.g., poultry, rabbit, fish, bee)	0.02 (9.2e-03; 0.02)	mitigating	0.97 [0.93; 1.02]	uncertain	0.62	0.25	0.26		
Farmworker	Smoking	0.24 (0.05; 1.86)	mitigating	0.04 [0.01; 0.11]	negative	0.66	2.3e-42	2.0e-41	protective factor	[9,10,12,15-17,27,31]
Farmworker	Stationary sawmill (e.g., edging, trimming, decking, debarking)	1.1e-03 (9.2e-05; 2.6e-03)	mitigating	0.98 [0.94; 1.03]	uncertain	0.62	0.15	0.18		
Farmworker	Sylviculture/forestry (e.g., thinning, pruning)	2.8e-03 (2.36e-03; 2.9e-03)	mitigating	0.99 [0.91; 1.07]	uncertain	0.62	0.21	0.23		
Farmworker	Training, dressage, stud farm	6.2e-03 (4.5e-03; 7.9e-03)	mitigating	0.97 [0.90; 1.06]	uncertain	0.62	0.19	0.21	conflicting results	[35-37]
Farmworker	Type 2 diabetes	0.03 (6.4e-03; 0.20)	promoting	2.16 [1.74; 2.68]	positive	0.63	7.8e-17	2.6e-16	risk factor	[10,12,15,16,25]
Farmworker	Unspecified farming (e.g., mixed farming, cereal farming, potato farming, pasture)	0.09 (0.03; 0.38)	promoting	1.07 [1.03; 1.15]	positive	0.64	1.3e-15	3.8e-15		
Farmworker	Unspecified specialized crop farming (e.g., soy farming, fruit arboriculture, truck farming)	0.11 (0.02; 0.48)	promoting	1.23 [1.12; 1.35]	positive	0.65	5.5e-24	3.0e-23		
Farmworker	Vinification	0.01 (0.01; 0.02)	promoting	1.02 [0.99; 1.12]	uncertain	0.62	0.06	0.10		
Farmworker	Viticulture	0.39 (0.06; 1.99)	promoting	1.50 [1.20; 1.73]	positive	0.74	1.6e-45	1.8e-44	conflicting results	[35,36,40]
Farmworker	Wood production (e.g., lopping)	3.7e-03 (2.4e-03; 3.8e-03)	mitigating	0.98 [0.90; 1.05]	uncertain	0.62	0.12	0.15		
Farmworker	Working in a company with fewer than 20 workers	0.03 (5.4e-03; 0.16)	mitigating	0.55 [0.46; 0.65]	negative	0.62	7.5e-18	3.0e-17		

95%CI: 95% confidence interval, HR: hazard ratio.

Table S10: Mean absolute SHAP value of each model's feature, directionality of each feature's effect on PD risk, univariate association with Cox model, and comparison with the literature for the sensitivity analysis 1

Population	Feature/Predictor	mean |SHAP| (95% CI)	SHAP direction	HR [95% CI]	Cox direction	Cox (C-index)	Cox p-value	Adjusted Cox p-value	Literature	Reference
Farm manager	Acetaminophen	0.18 (0.02; 0.38)	promoting	1.07 [1.06; 1.08]	positive	0.62	2.5e-04	4.6e-04	conflicting results	[1,2]
Farm manager	Age	0.22 (0.01; 0.52)	promoting	7.12 [4.75; 10.7]	positive	0.59	1.2e-24	3.1e-24	risk factor	[4,5]
Farm manager	Agricultural work companies (e.g., pesticide applications, harvest reaping)	6.0e-03 (3.5e-03; 6.7e-03)	promoting	1.02 [0.96; 1.09]	uncertain	0.60	0.12	0.13	uncertain	[35]
Farm manager	Antidepressant	0.05 (0.02; 0.29)	promoting	1.02 [1.01; 1.03]	positive	0.60	1.9e-03	3.0e-03	risk factor	[6,7]
Farm manager	Anxiolytics	0.04 (5.5e-03; 0.12)	promoting	1.00 [0.99; 1.01]	uncertain	0.59	0.09	0.10	risk factor	[6,7]
Farm manager	Aspirin	2.0e-03 (1.3e-04; 9.7e-03)	promoting	1.04 [1.03; 1.05]	positive	0.60	1.2e-05	2.4e-05	risk factor	[9,12]
Farm manager	Being a male	4.0e-03 (5.7e-04; 0.05)	promoting	1.17 [1.09; 1.25]	positive	0.59	1.2e-08	2.9e-08	risk factor	[14]
Farm manager	Bipolar disorder	2.9e-03 (1.5e-03; 3.3e-03)	promoting	12.1 [8.82; 16.5]	positive	0.60	1.04e-50	7.6e-50	risk factor	[12,15]
Farm manager	Calcium channel blocker	0.01 (3.7e-03; 0.12)	mitigating	0.95 [0.94; 0.97]	negative	0.60	6.8e-04	1.2e-03	protective factor	[9,12,15-18]
Farm manager	Cow farming	0.17 (0.06; 1.04)	promoting	1.06 [1.02; 1.11]	positive	0.60	4.2e-04	7.4e-04	risk factor	[35]
Farm manager	Crop farming (e.g., wheat, corn, and industrial grower)	0.50 (0.08; 3.32)	promoting	1.31 [1.15; 1.46]	positive	0.66	4.1e-08	9.5e-08	risk factor	[35,36,38]
Farm manager	Dairy farming	0.15 (0.05; 0.85)	promoting	1.04 [1.01; 1.09]	positive	0.59	6.8e-03	9.4e-03	conflicting results	[35,39]
Farm manager	Depression	7.4e-03 (3.2e-03; 8.3e-03)	promoting	3.87 [2.79; 5.36]	positive	0.60	2.2e-33	8.1e-33	risk factor	[9,10,12,20,21]
Farm manager	Epilepsy	0.04 (0.02; 0.06)	promoting	39.2 [33.3; 46.2]	positive	0.63	6.4e-203	1.4e-201	risk factor	[22,29,30]
Farm manager	Fluoroquinolones	2.6e-04 (3.8e-06; 2.9e-03)	promoting	0.74 [0.67; 0.83]	negative	0.60	1.4e-32	4.7e-32		
Farm manager	Fruit arboriculture	0.03 (0.02; 0.04)	promoting	1.03 [1.01; 1.07]	positive	0.60	2.6e-03	3.9e-03	risk factor	[35,36]
Farm manager	Gardening, landscaping and reforestation companies	2.2e-03 (9.8e-04; 5.4e-03)	mitigating	1.00 [0.92; 1.09]	uncertain	0.59	0.24	0.25	protective factor	[35]
Farm manager	HMG CoA reductase inhibitors (statin)	0.26 (0.06; 0.76)	mitigating	0.97 [0.95; 0.98]	negative	0.60	5.7e-03	8.4e-03	protective factor	[9,12,15,16,23]
Farm manager	Hypercholesterolemia	0.57 (0.31; 0.88)	promoting	85.0 [75.3; 96.0]	positive	0.67	3.7e-265	1.6e-263	protective factor	[24,31-34]
Farm manager	Hypertension	9.8e-03 (3.1e-04; 9.8e-03)	promoting	2.03 [1.66; 2.48]	positive	0.60	3.4e-32	1.0e-31	risk factor	[10,16,25]
Farm manager	Ibuprofen	0.02 (4.1e-03; 0.07)	mitigating	0.79 [0.73; 0.86]	negative	0.61	2.8e-34	1.2e-33	protective factor	[9,12,16]
Farm manager	Ischemic heart disease	3.0e-04 (6.2e-05; 2.4e-03)	promoting	1.15 [1.10; 1.30]	positive	0.59	6.8e-07	1.5e-06	risk factor	[15]
Farm manager	Long-term psychiatric conditions	6.4e-04 (3.0e-04; 7.9e-04)	promoting	6.13 [3.98; 9.43]	positive	0.60	1.6e-32	5.0e-32		
Farm manager	Macrolides and lincosamides antibiotics	8e-04 (1.1e-04; 3.6e-03)	mitigating	0.60 [0.52; 0.69]	negative	0.61	4.4e-40	2.2e-39	protective factor	[28]
Farm manager	Median farm surface (hectares)	0.12 (4.6e-03; 0.47)	promoting	1.13 [1.01; 1.56]	positive	0.60	0.04	4.9e-02		
Farm manager	Mixed cattle farming	0.04 (0.02; 0.10)	promoting	1.06 [1.03; 1.09]	positive	0.59	9.1e-03	0.01	risk factor	[35-37]
Farm manager	Non-aspirin NSAIDs	0.06 (0.02; 0.15)	mitigating	0.94 [0.93; 0.96]	negative	0.60	7.4e-04	1.2e-03	protective factor	[9,23,26]
Farm manager	Number of years of experience	0.19 (0.01; 0.98)	mitigating	0.04 [0.03; 0.07]	negative	0.64	6.1e-76	5.4e-75		
Farm manager	Other beta-lactam antibacterials	2.0e-04 (7.9e-05; 1.2e-03)	mitigating	0.74 [0.67; 0.82]	negative	0.60	7.9e-34	3.2e-33		
Farm manager	Overall antibiotics	0.20 (0.08; 0.42)	mitigating	0.84 [0.81; 0.87]	negative	0.63	1.3e-26	3.6e-26		
Farm manager	Ovine and caprine farming	0.05 (0.02; 0.06)	mitigating	0.98 [0.96; 1.01]	uncertain	0.59	0.06	0.07	uncertain	[35,37]
Farm manager	Penicillin antibiotics	2.5e-04 (5.0e-05; 9.1e-04)	mitigating	0.67 [0.61; 0.75]	negative	0.61	8.2e-41	4.5e-40		
Farm manager	Pig farming	5.2e-03 (3.4e-03; 5.5e-03)	promoting	1.02 [1.00; 1.05]	uncertain	0.59	0.05	0.06	conflicting results	[35-37]
Farm manager	Poultry and rabbit farming	7.3e-03 (4.7e-03; 9.6e-03)	mitigating	0.98 [0.95; 1.03]	uncertain	0.59	0.29	0.29	conflicting results	[35-37]
Farm manager	Smoking	0.18 (0.04; 1.34)	mitigating	0.07 [0.05; 0.12]	negative	0.63	1.7e-83	1.9e-82	protective factor	[9,10,12,15-17,27,31]
Farm manager	Stud farming	0.02 (0.01; 0.03)	mitigating	0.97 [0.94; 0.99]	negative	0.60	0.04	0.05	conflicting results	[35-37]
Farm manager	Sulfonamides	4.9e-03 (1.4e-03; 0.04)	mitigating	0.95 [0.93; 0.97]	negative	0.60	6.3e-03	8.9e-03	protective factor	[3]
Farm manager	Training, dressage, riding clubs	2.4e-03 (1.9e-03; 3.2e-03)	mitigating	0.96 [0.91; 1.04]	uncertain	0.59	0.25	0.26	protective factor	[35]
Farm manager	Truck farming, floriculture/flower-growing	0.05 (0.02; 0.14)	promoting	1.02 [0.97; 1.12]	uncertain	0.59	0.11	0.12	risk factor	[35]
Farm manager	Type 2 diabetes	6.9e-03 (6.3e-04; 0.04)	promoting	1.26 [1.07; 1.50]	positive	0.59	3.3e-05	6.3e-05	risk factor	[10,12,15,16,25]
Farm manager	Unspecified and mixed farming (e.g., polyculture, mixed farming, diversified farming)	0.17 (0.06; 1.18)	promoting	1.03 [1.01; 1.06]	positive	0.60	9.7e-03	0.01	risk factor	[35]
Farm manager	Viticulture	0.20 (0.07; 1.38)	promoting	1.12 [1.07; 1.28]	positive	0.60	8.8e-06	1.8e-05	conflicting results	[35,36,40]
Farm manager	Work diversification	0.44 (0.04; 1.21)	mitigating	0.18 [0.15; 0.21]	negative	0.70	1.1e-173	1.6e-172		
Farm manager	Working as a solidarity contributor	0.03 (3.7e-03; 0.11)	mitigating	0.57 [0.51; 0.64]	negative	0.62	3.0e-45	1.9e-44		
Farmworker	Acetaminophen	0.05 (6.8e-03; 0.19)	promoting	1.01 [0.99; 1.02]	uncertain	0.60	0.08	0.12	conflicting results	[1,2]
Farmworker	Age	0.18 (0.02; 0.37)	promoting	10.1 [4.76; 21.6]	positive	0.61	1.1e-06	2.8e-06	risk factor	[4,5]
Farmworker	Agricultural work companies (e.g., pesticide applications, harvest reaping)	1.7e-03 (1.3e-03; 2.4e-03)	promoting	1.02 [0.96; 1.22]	uncertain	0.61	0.26	0.35		

Farmworker	Antidepressant	0.09 (0.04; 0.60)	promoting	1.05 [1.04; 1.06]	positive	0.63	1.7e-11	7.5e-11	risk factor	[6,7]
Farmworker	Anxiolytics	0.07 (0.03; 0.23)	promoting	1.04 [1.03; 1.05]	positive	0.63	6.6e-11	2.6e-10	risk factor	[6,7]
Farmworker	Aspirin	7.5e-03 (1.2e-03; 0.02)	promoting	1.02 [1.01; 1.04]	positive	0.60	0.03	0.05	risk factor	[9,12]
Farmworker	Being a seasonal worker	0.36 (0.07; 0.71)	mitigating	0.16 [0.13; 0.20]	negative	0.75	8.4e-61	8.7e-60		
Farmworker	Bipolar disorder	3.5e-04 (1.2e-04; 5.1e-04)	promoting	16.5 [9.10; 30.1]	positive	0.61	3.4e-20	1.8e-19	risk factor	[12,15]
Farmworker	Depression	1.7e-03 (7.8e-04; 2.0e-03)	promoting	9.38 [6.23; 14.1]	positive	0.62	9.5e-27	5.9e-26	risk factor	[9,10,12,20,21]
Farmworker	Epilepsy	0.04 (0.02; 0.04)	promoting	101 [80.8; 126]	positive	0.66	8.5e-152	1.3e-150	risk factor	[22,29,30]
Farmworker	Fluoroquinolones	3.1e-04 (8.2e-05; 5.0e-04)	promoting	1.13 [1.04; 1.22]	positive	0.61	2.9e-03	5.6e-03		
Farmworker	Gardener, park ranger	3.5e-03 (2.3e-03; 3.9e-03)	mitigating	1.01 [0.87; 1.31]	uncertain	0.61	0.46	0.53		
Farmworker	Gardening and landscaping companies	2.9e-03 (2.5e-03; 3.4e-03)	mitigating	0.99 [0.88; 1.19]	uncertain	0.61	0.32	0.41		
Farmworker	HMG CoA reductase inhibitors (statin)	0.09 (0.03; 0.38)	mitigating	0.98 [0.97; 1.00]	negative	0.60	0.05	0.08	protective factor	[9,12,15,16,23]
Farmworker	Hypercholesterolemia	0.18 (0.16; 0.20)	promoting	221 [185; 265]	positive	0.70	1.8e-264	5.6e-263	conflicting results	[24,31-34]
Farmworker	Hypertension	2.7e-04 (2.2e-04; 3.0e-04)	promoting	2.71 [1.80; 4.07]	positive	0.61	1.7e-06	4.1e-06	risk factor	[10,16,25]
Farmworker	Ibuprofen	0.02 (8.7e-03; 0.09)	mitigating	0.96 [0.89; 1.05]	uncertain	0.61	0.38	0.45	protective factor	[9,12,16]
Farmworker	Long-term psychiatric conditions	7.2e-03 (3.5e-03; 8.5e-03)	promoting	20.0 [13.5; 29.7]	positive	0.62	3.9e-50	3.0e-49		
Farmworker	Non-aspirin NSAIDs	0.04 (3.8e-03; 0.10)	mitigating	1.00 [0.98; 1.02]	uncertain	0.61	0.10	0.14	protective factor	[9,23,26]
Farmworker	Number of years of experience	0.43 (0.02; 2.14)	mitigating	0.39 [0.15; 0.62]	negative	0.59	0.01	0.02		
Farmworker	Other beta-lactam antibacterials	2.8e-04 (1.5e-04; 1.7e-03)	promoting	1.02 [0.90; 1.16]	uncertain	0.61	0.79	0.82		
Farmworker	Overall antibiotics	0.11 (0.04; 0.25)	mitigating	1.01 [0.98; 1.04]	uncertain	0.60	0.68	0.73		
Farmworker	Penicillin antibiotics	9.4e-04 (9.7e-05; 4.7e-03)	promoting	0.95 [0.83; 1.07]	uncertain	0.61	0.37	0.45		
Farmworker	Smoking	0.16 (0.05; 1.15)	mitigating	0.05 [0.02; 0.14]	negative	0.64	5.6e-09	1.9e-08	protective factor	[9,10,12,15-17,27,31]
Farmworker	Sulfonamides	7.2e-04 (1.3e-04; 7.7e-03)	mitigating	0.91 [0.66; 1.27]	uncertain	0.61	0.60	0.66	protective factor	[3]
Farmworker	Type 2 diabetes	2.4e-03 (1.6e-04; 6.1e-03)	promoting	1.96 [1.51; 2.53]	positive	0.61	3.9e-07	1.1e-06	risk factor	[10,12,15,16,25]
Farmworker	Unspecified farming (e.g., mixed farming, cereal farming, potato farming, pasture)	0.04 (0.02; 0.15)	promoting	1.09 [1.04; 1.16]	positive	0.61	5.5e-04	1.1e-03		
Farmworker	Unspecified specialized crop farming (e.g., soy farming, fruit arboriculture, truck farming)	0.04 (0.02; 0.09)	promoting	1.04 [1.01; 1.12]	positive	0.61	0.04	0.07		
Farmworker	Viticulture	0.28 (0.04; 1.32)	promoting	1.32 [1.11; 1.45]	positive	0.61	1.2e-04	2.7e-04	conflicting results	[35,36,40]
Farmworker	Wood production (e.g., lopping)	3.1e-04 (2.6e-04; 4.2e-04)	mitigating	1.02 [0.72; 1.33]	uncertain	0.61	0.85	0.85	uncertain	[38]
Farmworker	Working in a company with fewer than 20 workers	2.9e-02 (9.1e-03; 0.13)	mitigating	0.55 [0.45; 0.68]	negative	0.62	9.8e-09	3.0e-08		

95%CI: 95% confidence interval, HR: hazard ratio.

Table S11: Mean absolute SHAP value of each model's feature, directionality of each feature's effect on PD risk, univariate association with Cox model, and comparison with the literature for the sensitivity analysis 2

Population	Feature/Predictor	mean |SHAP| (95% CI)	SHAP direction	HR [95% CI]	Cox direction	Cox (C-index)	Cox p-value	Adjusted Cox p-value	Literature	Reference
Farm manager	Acetaminophen	0.17 (0.02; 0.38)	promoting	1.06 [1.05; 1.08]	positive	0.62	1.4e-17	7.2e-17	conflicting results	[1,2]
Farm manager	Age	0.15 (5.5e-03; 0.39)	promoting	12.4 [7.41; 20.8]	positive	0.58	1.1e-21	7.5e-21	risk factor	[4,5]
Farm manager	Agricultural work companies (e.g., pesticide applications, harvest reaping)	3.8e-03 (2.4e-03; 4.3e-03)	promoting	1.02 [0.91; 1.31]	uncertain	0.58	0.68	0.70	uncertain	[35]
Farm manager	Antidepressant	0.06 (0.01; 0.29)	promoting	1.02 [1.01; 1.03]	positive	0.59	5.6e-04	8.8e-04	risk factor	[6,7]
Farm manager	Anxiolytics	0.06 (0.01; 0.20)	promoting	1.00 [0.99; 1.01]	uncertain	0.58	0.97	0.97	risk factor	[6,7]
Farm manager	Aspirin	0.01 (3.24e-03; 0.10)	promoting	0.96 [0.94; 0.98]	negative	0.59	4.1e-07	9.3e-07	risk factor	[9,12]
Farm manager	Being a male	5.3e-03 (5.5e-04; 0.02)	promoting	1.16 [1.05; 1.25]	positive	0.58	4.0e-03	5.9e-03	risk factor	[14]
Farm manager	Bipolar disorder	4.9e-03 (2.7e-03; 4.6e-03)	promoting	8.67 [5.51; 13.7]	positive	0.59	1.1e-20	6.4e-20	risk factor	[12,15]
Farm manager	Calcium channel blocker	0.01 (2.5e-03; 0.11)	mitigating	0.95 [0.93; 0.97]	negative	0.59	1.4e-07	3.6e-07	protective factor	[9,12,15-18]
Farm manager	Cow farming	0.17 (0.05; 1.31)	promoting	1.05 [1.02;.1.09]	positive	0.59	4.3e-05	7.7e-05	risk factor	[35]
Farm manager	Crop farming (e.g., wheat, corn, and industrial grower)	0.47 (0.04; 3.18)	promoting	1.13 [1.06; 1.20]	positive	0.62	3.6e-22	3.0e-21	risk factor	[35,36,38]
Farm manager	Dairy farming	0.09 (0.03; 0.53)	promoting	1.10 [1.06; 1.18]	positive	0.58	6.4e-09	1.9e-08	conflicting results	[35,39]
Farm manager	Depression	1.1e-03 (4.3e-04; 1.5e-03)	promoting	4.12 [2.81; 6.05]	positive	0.59	4.0e-13	1.3e-12	risk factor	[9,10,12,20,21]
Farm manager	Epilepsy	0.05 (0.02; 0.09)	promoting	38.1 [31.0; 46.9]	positive	0.62	1.0e-259	4.1e-258	risk factor	[22,29,30]
Farm manager	Fruit arboriculture	0.02 (0.01; 0.03)	promoting	1.08 [1.00; 1.20]	uncertain	0.58	0.07	0.09	risk factor	[35,36]
Farm manager	Gardening, landscaping and reforestation companies	4.1e-03 (1.7e-03; 0.01)	mitigating	1.06 [0.74; 1.67]	uncertain	0.58	0.53	0.57	protective factor	[35]
Farm manager	HMG CoA reductase inhibitors (statin)	0.26 (0.12; 0.70)	mitigating	0.96 [0.94; 0.97]	negative	0.59	1.6e-07	3.9e-07	protective factor	[9,12,15,16,23]
Farm manager	Hypercholesterolemia	0.07 (1.3e-03; 0.29)	promoting	87.6 [75.2; 102]	positive	0.65	9.3e-218	1.9e-216	conflicting results	[24,31-34]
Farm manager	Hypertension	3.0e-03 (7.2e-04; 0.02)	promoting	1.62 [1.23; 2.13]	positive	0.58	6.0e-04	9.1e-04	risk factor	[10,16,25]
Farm manager	Ibuprofen	0.02 (2.1e-03; 0.07)	mitigating	0.77 [0.69; 0.85]	negative	0.60	1.0e-06	2.2e-06	protective factor	[9,12,16]
Farm manager	Long-term psychiatric conditions	8.5e-04 (3.4e-04; 8.7e-04)	promoting	6.26 [3.93; 9.98]	positive	0.59	1.3e-14	4.4e-14		
Farm manager	Macrolides and lincosamides antibiotics	1.0e-03 (4.4e-04; 4.1e-03)	mitigating	0.76 [0.67; 0.88]	negative	0.59	1.1e-04	1.8e-04	protective factor	[28]
Farm manager	Median farm surface (hectares)	0.15 (7.1e-03; 0.47)	promoting	1.23 [1.06; 1.64]	positive	0.59	3.6e-05	6.7e-05		
Farm manager	Mixed cattle farming	0.03 (0.02; 0.03)	promoting	1.20 [0.88; 1.99]	uncertain	0.58	0.49	0.56	risk factor	[35-37]
Farm manager	Non-aspirin NSAIDs	0.05 (5.2e-03; 0.11)	mitigating	0.94 [0.91; 0.96]	negative	0.60	9.1e-08	2.5e-07	protective factor	[9,23,26]
Farm manager	Number of years of experience	0.19 (0.02; 0.42)	mitigating	0.06 [0.02; 0.11]	negative	0.62	8.3e-26	8.5e-25		
Farm manager	Overall antibiotics	0.20 (0.06; 0.42)	mitigating	0.87 [0.84; 0.90]	negative	0.62	1.5e-15	5.6e-15		
Farm manager	Ovine and caprine farming	0.05 (0.02; 0.18)	mitigating	0.97 [0.93; 0.98]	negative	0.58	0.04	0.05	uncertain	[35,37]
Farm manager	Penicillin antibiotics	2.7e-04 (1.5e-04; 1.8e-03)	mitigating	0.75 [0.67; 0.85]	negative	0.60	1.1e-06	2.3e-06		
Farm manager	Pig farming	6.3e-03 (4.6e-03; 7.2e-03)	promoting	1.06 [0.89; 1.34]	uncertain	0.58	0.20	0.24	conflicting results	[35,37]
Farm manager	Poultry and rabbit farming	6.2e-03 (4.4e-03; 8.1e-03)	mitigating	0.97 [0.78; 1.63]	uncertain	0.58	0.67	0.70	conflicting results	[35,37]
Farm manager	Smoking	0.20 (0.04; 1.53)	mitigating	0.09 [0.05; 0.16]	negative	0.62	8.5e-16	3.7e-15	protective factor	[9,10,12,15-17,27,31]
Farm manager	Stud farming	0.02 (0.01; 0.02)	mitigating	0.95 [0.91; 0.99]	negative	0.59	0.05	0.07	conflicting results	[35,37]
Farm manager	Sulfonamides	9.1e-03 (2.4e-03; 0.11)	mitigating	0.94 [0.91; 0.96]	negative	0.59	0.02	0.03	protective factor	[3]
Farm manager	Training, dressage, riding clubs	3.1e-03 (2.5e-03; 4.3e-03)	mitigating	0.96 [0.76; 1.39]	uncertain	0.58	0.53	0.57	protective factor	[35]
Farm manager	Truck farming, floriculture/flower-growing	0.02 (0.02; 0.03)	promoting	1.13 [0.86; 1.69]	uncertain	0.58	0.41	0.48	risk factor	[35]
Farm manager	Type 2 diabetes	4.7e-04 (2.5e-04; 1.3e-03)	promoting	1.23 [0.99; 1.52]	uncertain	0.58	0.06	0.08	risk factor	[10,12,15,16,25]
Farm manager	Unspecified and mixed farming (e.g., polyculture, mixed farming, diversified farming)	0.17 (0.06; 0.97)	promoting	1.08 [1.04; 1.13]	positive	0.58	3.5e-06	6.8e-06	risk factor	[35]
Farm manager	Viticulture	0.18 (4.7e-02; 1.48)	promoting	1.11 [1.04; 1.17]	positive	0.59	7.3e-05	1.3e-04	conflicting results	[35,36,40]
Farm manager	Work diversification	0.47 (4.2e-02; 1.38)	mitigating	0.20 [0.17; 0.24]	negative	0.68	1.3e-59	1.8e-58		
Farm manager	Working as a solidarity contributor	0.05 (1.7e-02; 0.24)	mitigating	0.57 [0.49; 0.65]	negative	0.63	8.9e-16	3.7e-15		
Farmworker	Acetaminophen	0.11 (1.5e-03; 0.40)	promoting	1.01 [0.98; 1.03]	uncertain	0.52	0.62	0.83	conflicting results	[1,2]
Farmworker	Age	0.20 (6.7e-03; 0.64)	promoting	16.6 [7.88; 35.2]	positive	0.54	1.7e-13	2.5e-13	risk factor	[4,5]
Farmworker	Agricultural work companies (e.g., pesticide applications, harvest reaping)	0.02 (0.01; 0.03)	promoting	0.99 [0.25; 3.92]	uncertain	0.53	0.99	0.99		
Farmworker	Antidepressant	0.11 (0.05; 0.50)	promoting	1.05 [1.02; 1.07]	positive	0.57	6.0e-05	4.2e-04	risk factor	[6,7]
Farmworker	Antiherpetic drugs	0.02 (3.8e-03; 0.13)	mitigating	0.85 [0.58; 1.23]	uncertain	0.51	0.39	0.72	protective factor	[8]
Farmworker	Anxiolytics	0.23 (0.08; 0.77)	promoting	1.04 [1.02; 1.05]	positive	0.60	5.9e-06	6.9e-05	risk factor	[6,7]

Farmworker	Aspirin	0.04 (5.1e-04; 0.67)	promoting	1.00 [0.97; 1.03]	uncertain	0.51	0.84	0.92	risk factor	[9,12]
Farmworker	Being a male	0.02 (5.0e-03; 0.07)	promoting	1.05 [0.78; 1.28]	uncertain	0.51	0.68	0.83	risk factor	[14]
Farmworker	Being a seasonal worker	0.47 (0.10; 0.95)	mitigating	0.24 [0.17; 0.33]	negative	0.68	3.2e-18	1.1e-16		
Farmworker	Calcium channel blocker	0.03 (2.4e-03; 0.23)	mitigating	1.01 [0.98; 1.05]	uncertain	0.51	0.56	0.83	protective factor	[9,12,15-18]
Farmworker	Fluoroquinolones	0.02 (2.6e-03; 0.08)	promoting	0.93 [0.74; 1.18]	uncertain	0.52	0.56	0.83		
Farmworker	Gardener, park ranger	0.03 (0.02; 0.03)	mitigating	0.98 [0.96; 1.01]	uncertain	0.51	0.26	0.51		
Farmworker	Gardening and landscaping companies	0.03 (1.9e-03; 0.34)	mitigating	0.99 [0.97; 1.00]	uncertain	0.51	0.43	0.75		
Farmworker	HMG CoA reductase inhibitors (statin)	0.05 (9.0e-04; 0.41)	mitigating	1.00 [0.97; 1.03]	uncertain	0.51	0.69	0.83	protective factor	[9,12,15,16,23]
Farmworker	Hypertension	3.1e-03 (1.9e-03; 4.3e-03)	promoting	2.74 [1.40; 5.33]	positive	0.53	3.1e-03	0.01	risk factor	[10,16,25]
Farmworker	Ibuprofen	0.05 (9.1e-03; 0.21)	mitigating	0.98 [0.88; 1.11]	uncertain	0.53	0.79	0.89	protective factor	[9,12,16]
Farmworker	Ischemic heart disease	0.01 (5.4e-03; 0.14)	promoting	1.54 [0.81; 2.91]	uncertain	0.52	0.18	0.44	risk factor	[15]
Farmworker	Macrolides and lincosamides antibiotics	8.6e-03 (2.0e-03; 0.05)	mitigating	0.95 [0.75; 1.20]	uncertain	0.53	0.65	0.83	protective factor	[28]
Farmworker	Non-aspirin NSAIDs	0.06 (2.6e-03; 0.19)	mitigating	0.99 [0.95; 1.03]	uncertain	0.53	0.72	0.84	protective factor	[9,23,26]
Farmworker	Number of years of experience	0.32 (6.7e-03; 1.74)	mitigating	0.29 [0.15; 0.65]	negative	0.52	6.1e-04	1.8e-03		
Farmworker	Other antibacterials	4.9e-03 (1.1e-03; 0.06)	mitigating	0.99 [0.78; 1.24]	uncertain	0.52	0.91	0.94		
Farmworker	Other beta-lactam antibacterials	3.4e-03 (1.0e-03; 0.02)	mitigating	1.01 [0.83; 1.24]	uncertain	0.51	0.90	0.94		
Farmworker	Overall antibiotics	0.16 (0.02; 0.40)	mitigating	0.96 [0.90; 1.02]	uncertain	0.55	0.21	0.46		
Farmworker	Penicillin antibiotics	0.04 (0.01; 0.21)	promoting	0.88 [0.71; 1.09]	uncertain	0.53	0.24	0.49		
Farmworker	Percentage of part-time job	0.04 (6.4e-05; 0.45)	mitigating	1.71 [0.82; 3.58]	uncertain	0.52	0.16	0.43		
Farmworker	Small animal farming (e.g., poultry, rabbit, fish, bee)	7.8e-03 (5.4e-03; 9.7e-03)	mitigating	0.98 [0.96; 1.05]	uncertain	0.51	0.48	0.8		
Farmworker	Smoking	0.24 (0.04; 1.92)	mitigating	0.32 [0.15; 0.57]	negative	0.56	3.5e-08	6.1e-07	protective factor	[9,10,12,15-17,27,31]
Farmworker	Sulfonamides	6.3e-03 (3.8e-03; 8.5e-03)	mitigating	1.03 [0.99; 1.07]	uncertain	0.52	0.19	0.44	protective factor	[3]
Farmworker	Type 2 diabetes	6.0e-03 (8.5e-04; 0.05)	promoting	1.53 [0.98; 2.40]	uncertain	0.52	0.06	0.18	risk factor	[10,12,15,16,25]
Farmworker	Unspecified farming (e.g., mixed farming, cereal farming, potato farming, pasture)	0.10 (0.03; 1.19)	promoting	1.12 [0.95; 1.53]	uncertain	0.52	0.69	0.83		
Farmworker	Unspecified specialized crop farming (e.g., soy farming, fruit arboriculture, truck farming)	0.11 (1.8e-03; 1.24)	promoting	1.08 [1.02; 1.15]	positive	0.51	0.02	0.07		
Farmworker	Vinification	8.0e-03 (4.4e-03; 0.01)	promoting	0.99 [0.35; 2.81]	uncertain	0.52	0.68	0.83		
Farmworker	Viticulture	0.37 (0.05; 2.20)	promoting	1.10 [1.03; 1.23]	positive	0.67	1.2e-03	6.0e-03	conflicting results	[35,36,40]
Farmworker	Working in a company with between 150 and 299 workers	9.0e-03 (5.0e-03; 0.01)	promoting	2.48 [1.27; 4.84]	positive	0.52	7.7e-03	0.03		
Farmworker	Working in a company with fewer than 20 workers	8.0e-03 (2.0e-03; 0.05)	mitigating	0.51 [0.37; 0.70]	negative	0.56	3.1e-05	2.7e-04		

95%CI: 95% confidence interval, HR: hazard ratio.

Table S12: Mean absolute SHAP value of each model's feature, directionality of each feature's effect on PD risk, univariate association with Cox model, and comparison with the literature for the sensitivity analysis 3

Population	Feature/Predictor	mean |SHAP| (95% CI)	SHAP direction	HR [95% CI]	Cox direction	Cox (C-index)	Cox p-value	Adjusted Cox p-value	Literature	Reference
Farm manager	Acetaminophen	0.20 (0.03; 0.42)	promoting	1.07 [1.06; 1.08]	positive	0.67	3.4e-32	2.3e-31	conflicting results	[1,2]
Farm manager	Age	0.25 (0.02; 0.56)	promoting	14.3 [10.1; 20.4]	positive	0.65	3.3e-59	2.6e-58	risk factor	[4,5]
Farm manager	Agricultural work companies (e.g., pesticide applications, harvest reaping)	8.0e-03 (3.8e-03; 8.4e-03)	promoting	1.01 [0.97; 1.06]	uncertain	0.65	0.36	0.39	uncertain	[35]
Farm manager	Antidepressant	0.07 (0.02; 0.40)	promoting	1.01 [1.00; 1.02]	uncertain	0.65	0.20	0.22	risk factor	[6,7]
Farm manager	Anxiolytics	0.03 (5.1e-03; 0.10)	promoting	1.00 [0.99; 1.00]	uncertain	0.65	0.43	0.44	risk factor	[6,7]
Farm manager	Aspirin	0.01 (0.02; 0.13)	promoting	1.03 [1.02; 1.04]	positive	0.65	7.0e-10	1.5e-09	risk factor	[9,12]
Farm manager	Bipolar disorder	4.5e-03 (2.7e-03; 5.7e-03)	promoting	7.01 [4.79; 10.2]	positive	0.65	9.5e-24	4.2e-23	risk factor	[12,15]
Farm manager	Calcium channel blocker	0.01 (1.3e-03; 0.13)	mitigating	0.95 [0.94; 0.97]	negative	0.65	7.2e-11	1.6e-10	protective factor	[9,12,15-18]
Farm manager	Cow farming	0.17 (0.06; 0.95)	promoting	1.28 [1.13; 1.37]	positive	0.65	7.0e-05	1.0e-04	risk factor	[35]
Farm manager	Crop farming (e.g., wheat, corn, and industrial grower)	0.56 (0.13; 3.44)	promoting	1.64 [1.41; 1.90]	positive	0.70	2.7e-61	2.7e-60	risk factor	[35,36,38]
Farm manager	Dairy farming	0.15 (0.04; 0.68)	promoting	1.31 [1.23; 1.52]	positive	0.64	3.9e-12	9.8e-12	conflicting results	[35,39]
Farm manager	Depression	5.8e-03 (2.1e-03; 6.5e-03)	promoting	4.66 [3.58; 6.08]	positive	0.66	5.8e-30	2.9e-29	risk factor	[9,10,12,20,21]
Farm manager	Epilepsy	0.03 (0.01; 0.05)	promoting	35.7 [30.6; 41.7]	positive	0.68	9.1e-198	1.8e-196	risk factor	[22,29,30]
Farm manager	Fruit arboriculture	0.03 (0.02; 0.04)	promoting	1.03 [1.01; 1.13]	positive	0.65	0.04	0.05	risk factor	[35,36]
Farm manager	Gardening, landscaping and reforestation companies	1.9e-03 (9.4e-04; 2.9e-03)	mitigating	0.98 [0.81; 1.69]	uncertain	0.65	0.43	0.44	protective factor	[35]
Farm manager	HMG CoA reductase inhibitors (statin)	0.27 (0.13; 0.67)	mitigating	0.96 [0.95; 0.97]	negative	0.65	6.9e-13	1.9e-12	protective factor	[9,12,15,16,23]
Farm manager	Hypercholesterolemia	0.53 (0.21; 0.84)	promoting	79.8 [71.3; 89.3]	positive	0.71	8.8e-242	3.5e-240	conflicting results	[24,31-34]
Farm manager	Hypertension	0.01 (2.6e-03; 0.08)	promoting	1.71 [1.40; 2.09]	positive	0.65	1.3e-07	2.3e-07	risk factor	[10,16,25]
Farm manager	Ibuprofen	0.03 (6.4e-03; 0.10)	mitigating	0.76 [0.70; 0.82]	negative	0.66	2.4e-11	5.7e-11	protective factor	[9,12,16]
Farm manager	Long-term psychiatric conditions	7.6e-04 (2.8e-04; 1.5e-03)	promoting	7.10 [4.74; 10.6]	positive	0.66	1.4e-21	5.1e-21		
Farm manager	Macrolides and lincosamides antibiotics	8.6e-04 (6.2e-06; 4.6e-03)	mitigating	0.72 [0.65; 0.81]	negative	0.65	9.4e-09	1.9e-08	protective factor	[28]
Farm manager	Median farm surface (hectares)	0.09 (3.4e-03; 0.47)	promoting	1.21 [1.11; 1.96]	positive	0.65	5.1e-08	9.3e-08		
Farm manager	Mixed cattle farming	0.03 (0.02; 0.03)	promoting	1.51 [1.05; 2.09]	positive	0.65	7.2e-13	1.9e-12	risk factor	[35-37]
Farm manager	Non-aspirin NSAIDs	0.05 (4.1e-03; 0.14)	mitigating	0.93 [0.92; 0.95]	negative	0.65	1.7e-13	5.2e-13	protective factor	[9,23,26]
Farm manager	Number of years of experience	0.18 (6.6e-03; 0.57)	mitigating	0.98 [0.96; 0.99]	negative	0.66	0.05	0.06		
Farm manager	Other antibacterials	6.9e-04 (2.0e-04; 3.1e-03)	mitigating	0.79 [0.70; 0.88]	negative	0.65	5.9e-05	9.1e-05		
Farm manager	Overall antibiotics	0.18 (0.07; 0.38)	mitigating	0.85 [0.82; 0.87]	negative	0.66	5.4e-31	3.1e-30		
Farm manager	Ovine and caprine farming	0.05 (0.03; 0.06)	mitigating	0.97 [0.95; 0.99]	negative	0.65	2.3e-07	3.8e-07	uncertain	[35,37]
Farm manager	Penicillin antibiotics	1.5e-04 (2.4e-05; 1.2e-03)	mitigating	0.64 [0.58; 0.71]	negative	0.66	2.5e-18	8.3e-18		
Farm manager	Pig farming	4.9e-03 (3.6e-03; 5.7e-03)	promoting	1.02 [0.98; 1.12]	uncertain	0.65	0.16	0.18	conflicting results	[35-37]
Farm manager	Poultry and rabbit farming	7.8e-03 (4.8e-03; 9.4e-03)	mitigating	0.99 [0.89; 1.10]	uncertain	0.65	0.56	0.56	conflicting results	[35-37]
Farm manager	Stud farming	0.02 (0.01; 0.03)	mitigating	0.95 [0.91; 0.99]	negative	0.65	3.8e-08	7.2e-08	conflicting results	[35-37]
Farm manager	Sulfonamides	2.8e-03 (4.9e-04; 0.03)	mitigating	0.96 [0.94; 0.97]	negative	0.65	3.1e-07	5.0e-07	protective factor	[3]
Farm manager	Training, dressage, riding clubs	3.3e-03 (2.6e-03; 4.3e-03)	mitigating	0.98 [0.96; 0.99]	negative	0.65	0.05	0.06	protective factor	[35]
Farm manager	Truck farming, floriculture/flower-growing	0.04 (0.02; 0.05)	promoting	1.02 [1.01; 1.05]	positive	0.65	2.1e-03	3.0e-03	risk factor	[35]
Farm manager	Type 2 diabetes	1.6e-03 (6.2e-06; 0.01)	promoting	1.22 [1.04; 1.43]	positive	0.65	0.01	0.01	risk factor	[10,12,15,16,25]
Farm manager	Unspecified and mixed farming (e.g., polyculture, mixed farming, diversified farming)	0.17 (0.06; 1.12)	promoting	1.04 [1.01; 1.19]	positive	0.65	0.02	0.03	risk factor	[35]
Farm manager	Viticulture	0.21 (0.08; 0.81)	promoting	1.11 [1.04; 1.35]	positive	0.66	2.7e-3	3.7e-03	conflicting results	[35,36,40]
Farm manager	Work diversification	0.47 (0.06; 1.22)	mitigating	0.18 [0.16; 0.21]	negative	0.73	1.5e-112	2.0e-111		
Farm manager	Working as a solidarity contributor	0.02 (3.3e-03; 0.11)	mitigating	0.60 [0.54; 0.66]	negative	0.67	2.9e-23	1.2e-22		
Farmworker	Acetaminophen	0.09 (5.1e-03; 0.37)	promoting	1.01 [1.00; 1.02]	uncertain	0.63	0.24	0.39	conflicting results	[1,2]
Farmworker	Age	0.43 (0.03; 0.93)	promoting	15.4 [7.28; 32.5]	positive	0.63	1.3e-14	6.0e-14	risk factor	[4,5]
Farmworker	Agricultural work companies (e.g., pesticide applications, harvest reaping)	0.02 (0.01; 0.03)	promoting	1.02 [0.98; 1.07]	uncertain	0.63	0.36	0.50		
Farmworker	Antidepressant	0.05 (6.8e-03; 0.38)	promoting	1.06 [1.05; 1.07]	positive	0.66	5.5e-28	3.6e-27	risk factor	[6,7]
Farmworker	Antiherpetic drugs	2.0e-04 (1.1e-04; 2.2e-04)	mitigating	1.04 [0.95; 1.14]	uncertain	0.63	0.41	0.56	protective factor	[8]
Farmworker	Anxiolytics	0.13 (0.05; 0.41)	promoting	1.04 [1.03; 1.05]	positive	0.65	2.0e-23	1.0e-22	risk factor	[6,7]
Farmworker	Aspirin	9.3e-03 (3.7e-04; 0.02)	promoting	1.00 [0.99; 1.02]	uncertain	0.63	0.82	0.86	risk factor	[9,12]

Farmworker	Being a male	0.02 (2.4e-03; 0.05)	promoting	1.01 [0.88; 1.16]	uncertain	0.63	0.91	0.92	risk factor	[14]
Farmworker	Being a seasonal worker	0.37 (0.08; 0.80)	mitigating	0.23 [0.19; 0.27]	negative	0.72	9.6e-70	1.5e-68		
Farmworker	Bipolar disorder	7.6e-03 (4.7e-03; 8.1e-03)	promoting	15.9 [9.35; 26.9]	positive	0.64	1.2e-24	6.9e-24	risk factor	[12,15]
Farmworker	Calcium channel blocker	9.9e-03 (2.8e-03; 0.09)	mitigating	1.01 [1.00; 1.03]	uncertain	0.63	0.12	0.22	protective factor	[9,12,15-18]
Farmworker	Cancer	2.4e-03 (5.3e-04; 0.03)	promoting	1.66 [1.29; 2.14]	positive	0.63	9.5e-05	2.7e-04	protective factor	[15]
Farmworker	Depression	4.5e-03 (1.5e-03; 4.8e-03)	promoting	8.68 [5.99; 12.6]	positive	0.64	4.3e-30	3.3e-29	risk factor	[9,10,12,20,21]
Farmworker	Epilepsy	0.01 (4.1e-04; 0.02)	promoting	78.9 [64.0; 97.3]	positive	0.67	7.4e-165	1.7e-163	risk factor	[22,29,30]
Farmworker	Fluoroquinolones	6.3e-03 (1.2e-03; 0.02)	promoting	1.09 [1.00; 1.18]	uncertain	0.63	0.05	0.10		
Farmworker	Gamekeeper, fishery officer	1.0e-03 (6.6e-04; 8.1e-04)	mitigating	1.05 [0.78; 1.46]	uncertain	0.63	0.65	0.73		
Farmworker	Gardener, park ranger	0.04 (0.03; 0.04)	mitigating	0.98 [0.96; 0.99]	negative	0.63	0.04	0.09		
Farmworker	Gardening and landscaping companies	0.04 (0.02; 0.04)	mitigating	0.99 [0.95; 1.06]	uncertain	0.63	0.08	0.15		
Farmworker	HMG CoA reductase inhibitors (statin)	0.14 (0.07; 0.72)	mitigating	0.98 [0.97; 0.99]	negative	0.63	0.01	2.3e-02	protective factor	[9,12,15,16,23]
Farmworker	Hypercholesterolemia	0.25 (0.21; 0.33)	promoting	210 [180; 246]	positive	0.70	2.3e-254	1.1e-252	conflicting results	[24,31-34]
Farmworker	Hypertension	6.8e-03 (1.1e-03; 7.2e-03)	promoting	2.83 [2.00; 4.00]	positive	0.63	3.4e-47	3.9e-46	risk factor	[10,16,25]
Farmworker	Ibuprofen	0.04 (0.02; 0.19)	mitigating	0.90 [0.82; 0.99]	negative	0.63	8.7e-04	2.4e-03	protective factor	[9,12,16]
Farmworker	Ischemic heart disease	4.1e-04 (2.9e-04; 4.2e-04)	promoting	2.04 [1.50; 2.78]	positive	0.63	1.6e-14	6.7e-14	risk factor	[15]
Farmworker	Large animal farming (e.g., cattle, ovine, pig)	5.8e-03 (4.0e-03; 7.2e-03)	promoting	1.03 [0.97; 1.08]	uncertain	0.63	0.22	0.38		
Farmworker	Long-term psychiatric conditions	9.3e-03 (5.0e-03; 9.6e-03)	promoting	13.2 [8.58; 20.4]	positive	0.64	1.8e-31	1.7e-30		
Farmworker	Macrolides and lincosamides antibiotics	1.5e-03 (1.9e-04; 3.6e-03)	promoting	0.97 [0.85; 1.11]	uncertain	0.63	0.64	0.73		
Farmworker	Non-aspirin NSAIDs	0.06 (9.6e-03; 0.20)	mitigating	1.01 [0.99; 1.03]	uncertain	0.63	0.50	0.62	protective factor	[9,23,26]
Farmworker	Number of years of experience	0.83 (0.02; 2.61)	mitigating	0.38 [0.17; 0.72]	negative	0.63	4.5e-04	5.9e-04		
Farmworker	Other beta-lactam antibacterials	1.0e-03 (6.4e-04; 5.9e-03)	promoting	1.02 [0.90; 1.14]	uncertain	0.63	0.69	0.76		
Farmworker	Overall antibiotics	0.15 (0.05; 0.45)	mitigating	1.00 [0.97; 1.03]	uncertain	0.63	0.92	0.92		
Farmworker	Penicillin antibiotics	1.9e-03 (5.4e-05; 9.5e-03)	promoting	0.95 [0.85; 1.06]	uncertain	0.63	0.33	0.47		
Farmworker	Percentage of part-time job	0.02 (3.6e-04; 0.23)	mitigating	0.79 [0.52; 0.90]	negative	0.62	3.1e-05	1.0e-04		
Farmworker	Shellfish farming (e.g., oyster farming, scallop aquaculture)	0.01 (9.7e-03; 0.02)	mitigating	0.99 [0.98; 1.00]	uncertain	0.63	0.32	0.47		
Farmworker	Small animal farming (e.g., poultry, rabbit, fish, bee)	0.01 (7.9e-03; 0.01)	mitigating	0.99 [0.99; 1.00]	uncertain	0.63	0.72	0.77		
Farmworker	Stationary sawmill (e.g., edging, trimming, decking, debarking)	1.4e-03 (6.5e-04; 4.7e-03)	mitigating	0.99 [0.98; 1.00]	uncertain	0.63	0.28	0.44		
Farmworker	Sulfonamides	2.7e-04 (8.8e-05; 2.5e-03)	mitigating	1.01 [0.98; 1.04]	uncertain	0.63	0.14	0.25	protective factor	[3]
Farmworker	Sulfonamides and trimethoprim antibiotics	9.5e-05 (6.8e-05; 9.5e-05)	mitigating	1.03 [0.85; 1.25]	uncertain	0.63	0.56	0.66		
Farmworker	Sylviculture/forestry (e.g., thinning, pruning)	1.3e-03 (8.7e-04; 1.3e-03)	mitigating	0.99 [0.88; 1.06]	uncertain	0.63	0.47	0.60		
Farmworker	Training, dressage, stud farm	7.3e-03 (5.9e-03; 8.3e-03)	mitigating	0.98 [0.80; 1.05]	uncertain	0.63	0.33	0.47	conflicting results	[35-37]
Farmworker	Type 2 diabetes	0.03 (4.5e-03; 0.13)	promoting	2.09 [1.67; 2.61]	positive	0.64	1.1e-10	3.9e-10	risk factor	[10,12,15,16,25]
Farmworker	Unspecified farming (e.g., mixed farming, cereal farming, potato farming, pasture)	0.10 (0.03; 0.36)	promoting	1.07 [1.03; 1.12]	positive	0.65	2.9e-03	7.4e-03		
Farmworker	Unspecified specialized crop farming (e.g., soy farming, fruit arboriculture, truck farming)	0.12 (0.02; 0.65)	promoting	1.15 [1.03; 1.26]	positive	0.66	0.01	2.3e-02		
Farmworker	Vinification	0.01 (0.01; 0.02)	promoting	1.01 [1.01; 1.04]	positive	0.63	0.05	0.10		
Farmworker	Viticulture	0.44 (0.03; 2.08)	promoting	1.22 [1.11; 1.44]	positive	0.74	4.6e-05	1.4e-04	conflicting results	[35,36,40]
Farmworker	Wood production (e.g., lopping)	9.1e-03 (6.7e-03; 9.9e-03)	mitigating	0.99 [0.97; 1.00]	uncertain	0.63	0.51	0.62	uncertain	[38]
Farmworker	Working in a company with fewer than 20 workers	0.04 (0.01; 0.19)	mitigating	0.56 [0.47; 0.66]	negative	0.63	2.4e-11	9.2e-11		

95%CI: 95% confidence interval, HR: hazard ratio.

Table S13: Mean absolute SHAP value of each model's feature, directionality of each feature's effect on PD risk, univariate association with Cox model, and comparison with the literature for the sensitivity analysis 4

Population	Feature/Predictor	mean |SHAP| (95% CI)	SHAP direction	HR [95% CI]	Cox direction	Cox (C-index)	Cox p-value	Adjusted Cox p-value	Literature	Reference
Farm manager	Acetaminophen	0.20 (0.03; 0.44)	promoting	1.06 [1.05; 1.07]	positive	0.58	6.2e-29	3.0e-28	conflicting results	[1,2]
Farm manager	Agricultural work companies (e.g., pesticide applications, harvest reaping)	6.4e-03 (4.9e-03; 8.8e-03)	promoting	1.01 [0.93; 1.07]	uncertain	0.50	0.32	0.37	uncertain	[35]
Farm manager	Antidepressant	0.06 (0.03; 0.40)	promoting	1.01 [1.00; 1.02]	positive	0.53	0.04	0.05	risk factor	[6,7]
Farm manager	Anxiolytics	0.03 (7.7e-03; 0.10)	promoting	1.00 [1.00; 1.01]	uncertain	0.52	0.41	0.43	risk factor	[6,7]
Farm manager	Aspirin	7.8e-03 (1.7e-03; 0.09)	promoting	1.03 [1.02; 1.04]	positive	0.51	6.3e-09	1.4e-08	risk factor	[9,12]
Farm manager	Bipolar disorder	3.8e-03 (2.6e-03; 4.7e-03)	promoting	10.8 [7.99; 14.5]	positive	0.51	6.9e-55	5.4e-54	risk factor	[12,15]
Farm manager	Calcium channel blocker	0.02 (2.4e-03; 0.17)	mitigating	0.96 [0.94; 0.97]	negative	0.51	6.8e-09	1.4e-08	protective factor	[9,12,15-18]
Farm manager	Cancer	1.1e-03 (1.0e-04; 5.3e-03)	mitigating	0.97 [0.82; 1.15]	uncertain	0.50	0.72	0.72	protective factor	[15]
Farm manager	Cow farming	0.16 (0.06; 1.00)	promoting	1.16 [1.06; 1.32]	positive	0.52	8.8e-03	0.01	risk factor	[35]
Farm manager	Crop farming (e.g., wheat, corn, and industrial grower)	0.54 (0.15; 3.41)	promoting	1.60 [1.35; 1.86]	positive	0.56	1.7e-47	1.1e-46	risk factor	[35,36,38]
Farm manager	Dairy farming	0.15 (0.05; 0.80)	promoting	1.23 [1.12; 1.43]	positive	0.50	4.5e-13	1.3e-12	conflicting results	[35,39]
Farm manager	Depression	4.8e-03 (1.2e-03; 5.4e-03)	promoting	4.30 [3.23; 5.72]	positive	0.51	1.7e-23	6.6e-23	risk factor	[9,10,12,20,21]
Farm manager	Epilepsy	0.03 (0.01; 0.04)	promoting	35.0 [29.9; 41.0]	positive	0.54	4.1e-198	8.0e-197	risk factor	[22,29,30]
Farm manager	Fluoroquinolones	3.6e-04 (1.7e-05; 4.5e-03)	mitigating	0.75 [0.68; 0.83]	negative	0.52	9.2e-09	1.8e-08		
Farm manager	Fruit arboriculture	0.03 (0.02; 0.04)	promoting	1.05 [1.02; 1.11]	positive	0.50	1.2e-03	1.8e-03	risk factor	[35,36]
Farm manager	Gardening, landscaping and reforestation companies	6.5e-03 (3.5e-03; 0.02)	mitigating	0.98 [0.96; 0.99]	negative	0.50	0.03	0.04	protective factor	[35]
Farm manager	HMG CoA reductase inhibitors (statin)	0.25 (0.10; 0.73)	mitigating	0.96 [0.95; 0.97]	negative	0.51	5.5e-11	1.34e-10	protective factor	[9,12,15,16,23]
Farm manager	Hypercholesterolemia	0.50 (0.22; 0.83)	promoting	87.6 [78.5; 97.8]	positive	0.59	2.2e-208	8.6e-207	conflicting results	[24,31-34]
Farm manager	Hypertension	0.02 (5.3e-03; 0.18)	promoting	1.79 [1.47; 2.20]	positive	0.51	1.3e-08	2.4e-08	risk factor	[10,16,25]
Farm manager	Hypothyroidism	4.9e-04 (8.2e-05; 3.4e-03)	promoting	0.95 [0.78; 1.16]	uncertain	0.50	0.62	0.64	risk factor	[25,33]
Farm manager	Ibuprofen	0.03 (7.1e-03; 0.12)	mitigating	0.69 [0.63; 0.75]	negative	0.55	7.9e-17	2.8e-16	protective factor	[9,12,16]
Farm manager	Long-term psychiatric conditions	2.9e-03 (1.5e-03; 3.1e-03)	promoting	5.82 [3.89; 8.71]	positive	0.50	9.5e-18	3.1e-17		
Farm manager	Median farm surface (hectares)	0.07 (9.6e-04; 0.67)	promoting	1.13 [1.05; 1.58]	positive	0.48	2.9e-07	5.1e-07		
Farm manager	Mixed cattle farming	0.03 (0.02; 0.04)	promoting	1.25 [1.09; 1.78]	positive	0.50	3.0e-10	6.9e-10	risk factor	[35-37]
Farm manager	Non-aspirin NSAIDs	0.05 (1.0e-03; 0.12)	mitigating	0.92 [0.90; 0.93]	negative	0.57	5.9e-19	2.1e-18	protective factor	[9,23,26]
Farm manager	Number of years of experience	0.20 (0.02; 0.55)	mitigating	0.79 [0.64; 0.90]	negative	0.63	1.5e-56	1.5e-55		
Farm manager	Overall antibiotics	0.17 (0.04; 0.38)	mitigating	0.84 [0.81; 0.86]	negative	0.59	6.6e-35	3.7e-34		
Farm manager	Ovine and caprine farming	0.05 (0.02; 0.06)	mitigating	0.95 [0.91; 0.97]	negative	0.50	1.6e-05	2.7e-05	uncertain	[35,37]
Farm manager	Pig farming	6.8e-03 (5.1e-03; 7.5e-03)	promoting	1.03 [0.92; 1.42]	uncertain	0.50	0.27	0.32	conflicting results	[35-37]
Farm manager	Poultry and rabbit farming	0.01 (6.2e-03; 0.01)	mitigating	0.95 [0.83; 1.03]	uncertain	0.50	0.09	0.11	conflicting results	[35-37]
Farm manager	Smoking	0.17 (0.04; 1.30)	mitigating	0.08 [0.05; 0.12]	negative	0.55	2.1e-27	9.1e-27	protective factor	[9,10,12,15-17,27,31]
Farm manager	Stud farming	0.02 (0.01; 0.03)	mitigating	0.95 [0.92; 0.97]	negative	0.51	5.7e-05	8.9e-05		
Farm manager	Sulfonamides	6.4e-04 (1.3e-04; 2.2e-03)	mitigating	0.97 [0.95; 0.98]	negative	0.51	5.0e-05	8.1e-05	protective factor	[3]
Farm manager	Training, dressage, riding clubs	4.2e-03 (3.0e-03; 5.3e-03)	mitigating	0.98 [0.93; 0.99]	negative	0.50	0.03	0.04	protective factor	[35]
Farm manager	Truck farming, floriculture/flower-growing	0.04 (0.02; 0.05)	promoting	1.03 [0.94; 1.24]	uncertain	0.50	0.39	0.42	risk factor	[35]
Farm manager	Unspecified and mixed farming (e.g., polyculture, mixed farming, diversified farming)	0.18 (0.04; 1.04)	promoting	1.07 [0.93; 1.52]	uncertain	0.52	0.37	0.41	risk factor	[35]
Farm manager	Viticulture	0.20 (0.07; 0.96)	promoting	1.18 [1.08; 1.34]	positive	0.51	9.4e-13	2.4e-12	conflicting results	[35,36,40]
Farm manager	Work diversification	0.47 (0.06; 1.21)	mitigating	0.20 [0.17; 0.23]	negative	0.62	2.3e-107	3.0e-106		
Farm manager	Working as a solidarity contributor	0.04 (2.1e-03; 0.15)	mitigating	0.95 [0.87; 1.04]	uncertain	0.50	0.23	0.28		
Farmworker	Acetaminophen	0.09 (4.1e-03; 0.34)	promoting	1.01 [1.00; 1.03]	positive	0.55	0.05	0.09	conflicting results	[1,2]
Farmworker	Agricultural work companies (e.g., pesticide applications, harvest reaping)	0.02 (0.01; 0.03)	promoting	1.02 [1.00; 1.03]	positive	0.51	0.05	0.09		
Farmworker	Antidepressant	0.08 (0.03; 0.60)	promoting	1.05 [1.03; 1.06]	positive	0.57	6.0e-14	2.9e-13	risk factor	[6,7]
Farmworker	Anxiolytics	0.11 (0.03; 0.30)	promoting	1.04 [1.03; 1.04]	positive	0.59	1.6e-16	8.7e-16	risk factor	[6,7]
Farmworker	Aspirin	9.1e-03 (2.9e-04; 0.04)	promoting	1.01 [1.00; 1.03]	uncertain	0.54	0.06	0.09	risk factor	[9,12]
Farmworker	Being a seasonal worker	0.35 (0.07; 0.80)	mitigating	0.28 [0.23; 0.33]	negative	0.63	2.1e-53	3.1e-52		
Farmworker	Bipolar disorder	5.3e-03 (2.7e-03; 7.3e-03)	promoting	23.4 [14.7; 37.3]	positive	0.51	5.3e-40	4.7e-39	risk factor	[12,15]
Farmworker	Calcium channel blocker	9.7e-03 (4.2e-03; 0.01)	mitigating	1.01 [1.00; 1.03]	uncertain	0.52	0.06	0.09	protective factor	[9,12,15-18]

Farmworker	Cancer	4.5e-03 (9.9e-04; 0.04)	promoting	1.60 [1.24; 2.06]	positive	0.52	2.6e-04	6.7e-04	protective factor	[15]
Farmworker	Depression	5.5e-03 (2.9e-03; 5.6e-03)	promoting	6.70 [4.53; 9.89]	positive	0.51	1.2e-21	7.5e-21	risk factor	[9,10,12,20,21]
Farmworker	Epilepsy	0.01(5.3e-04; 0.02)	promoting	73.8 [60.0; 90.8]	positive	0.55	8.2e-101	1.8e-99	risk factor	[22,29,30]
Farmworker	Fluoroquinolones	8.2e-04 (2.0e-04; 3.9e-03)	promoting	1.07 [0.98; 1.18]	uncertain	0.52	0.14	0.20		
Farmworker	Gardener, park ranger	0.04 (0.02; 0.05)	mitigating	0.97 [0.93; 0.99]	negative	0.50	0.04	0.08		
Farmworker	Gardening and landscaping companies	0.04 (0.02; 0.04)	mitigating	1.01 [0.93; 1.15]	uncertain	0.50	0.09	0.13		
Farmworker	HMG CoA reductase inhibitors (statin)	0.14 (0.05; 0.77)	mitigating	0.98 [0.97; 0.99]	negative	0.54	4.7e-03	9.4e-03	protective factor	[9,12,15,16,23]
Farmworker	Hypercholesterolemia	0.20 (0.17; 0.29)	promoting	217 [186; 254]	positive	0.61	2.2e-187	9.7e-186	conflicting results	[24,31-34]
Farmworker	Hypertension	6.4e-03 (2.6e-03; 9.3e-03)	promoting	3.40 [2.45; 4.70]	positive	0.52	1.6e-13	7.0e-13	risk factor	[10,16,25]
Farmworker	Ibuprofen	0.04 (0.01; 0.17)	mitigating	0.87 [0.79; 0.96]	negative	0.51	3.8e-03	8.4e-03	protective factor	[9,12,16]
Farmworker	Ischemic heart disease	3.9e-03 (4.9e-04; 4.9e-03)	promoting	1.62 [1.17; 2.25]	positive	0.51	4.1e-03	8.6e-03	risk factor	[15]
Farmworker	Large animal farming (e.g., cattle, ovine, pig)	5.3e-03 (4.3e-03; 6.5e-03)	promoting	1.05 [0.95; 1.22]	uncertain	0.50	0.20	0.27		
Farmworker	Long-term psychiatric conditions	0.01 (6.4e-03; 0.01)	promoting	15.9 [11.0; 23.0]	positive	0.51	1.6e-48	1.8e-47		
Farmworker	Macrolides and lincosamides antibiotics	6.7e-03 (1.1e-03; 0.02)	promoting	1.04 [0.93; 1.16]	uncertain	0.51	0.53	0.61	protective factor	[28]
Farmworker	Non-aspirin NSAIDs	0.05 (4.2e-03; 0.13)	mitigating	1.00 [0.98; 1.02]	uncertain	0.47	0.74	0.79	protective factor	[9,23,26]
Farmworker	Number of years of experience	0.87 (0.03; 2.57)	mitigating	0.48 [0.33; 0.77]	negative	0.56	2.9e-04	3.7e-04		
Farmworker	Other antibacterials	1.9e-03 (9.8e-04; 0.01)	promoting	0.96 [0.83; 1.12]	uncertain	0.50	0.61	0.69		
Farmworker	Overall antibiotics	0.14 (0.06; 0.36)	mitigating	1.00 [0.97; 1.03]	uncertain	0.47	0.98	0.98		
Farmworker	Penicillin antibiotics	1.3e-03 (3.0e-04; 0.01)	promoting	0.93 [0.84; 1.04]	uncertain	0.49	0.20	0.27		
Farmworker	Percentage of part-time job	0.02 (3.0e-03; 0.17)	mitigating	0.86 [0.55; 0.92]	negative	0.51	2.2e-05	6.1e-05		
Farmworker	Shellfish farming (e.g., oyster farming, scallop aquaculture)	0.01 (0.01; 0.02)	mitigating	0.97 [0.92; 1.01]	uncertain	0.50	0.06	0.09		
Farmworker	Small animal farming (e.g., poultry, rabbit, fish, bee)	0.02 (9.2e-03; 0.02)	mitigating	1.00 [0.94; 1.07]	uncertain	0.50	0.83	0.85		
Farmworker	Smoking	0.24 (0.05; 1.86)	mitigating	0.04 [0.01; 0.11]	negative	0.55	1.2e-10	4.1e-10	protective factor	[9,10,12,15-17,27,31]
Farmworker	Stationary sawmill (e.g., edging, trimming, decking, debarking)	1.1e-03 (9.2e-05; 2.6e-03)	mitigating	1.00 [0.90; 1.11]	uncertain	0.50	0.35	0.43		
Farmworker	Sulfonamides		mitigating	0.96 [0.94; 0.99]	negative	0.52	1.5e-03	3.5e-03	protective factor	[3]
Farmworker	Sulfonamides and trimethoprim antibiotics		mitigating	1.03 [0.86; 1.22]	uncertain	0.51	0.77	0.81		
Farmworker	Sylviculture/forestry (e.g., thinning, pruning)	2.8e-03 (2.36e-03; 2.9e-03)	mitigating	1.02 [0.92; 1.13]	uncertain	0.50	0.68	0.75		
Farmworker	Training, dressage, stud farm	6.2e-03 (4.5e-03; 7.9e-03)	mitigating	0.99 [0.88; 1.08]	uncertain	0.50	0.49	0.58	conflicting results	[35-37]
Farmworker	Type 2 diabetes	0.03 (6.4e-03; 0.20)	promoting	2.11 [1.70; 2.62]	positive	0.53	1.18e-11	4.3e-11	risk factor	[10,12,15,16,25]
Farmworker	Unspecified farming (e.g., mixed farming, cereal farming, potato farming, pasture)	0.09 (0.03; 0.38)	promoting	1.12 [1.05; 1.20]	positive	0.52	9.0e-07	2.6e-06		
Farmworker	Unspecified specialized crop farming (e.g., soy farming, fruit arboriculture, truck farming)	0.11 (0.02; 0.48)	promoting	1.27 [1.10; 1.41]	positive	0.54	4.5e-11	1.7e-10		
Farmworker	Vinification	0.01 (0.01; 0.02)	promoting	1.01 [0.99; 1.14]	uncertain	0.50	0.06	0.09		
Farmworker	Viticulture	0.39 (0.06; 1.99)	promoting	1.63 [1.27; 1.90]	positive	0.64	5.7e-38	4.2e-37	conflicting results	[35,36,40]
Farmworker	Wood production (e.g., lopping)	3.7e-03 (2.4e-03; 3.8e-03)	mitigating	1.00 [0.90; 1.06]	uncertain	0.50	0.29	0.37	uncertain	[38]
Farmworker	Working in a company with between 50 and 149 workers		promoting	1.64 [1.23; 2.20]	positive	0.51	8.2e-04	2.0e-03		
Farmworker	Working in a company with fewer than 20 workers	0.03 (5.4e-03; 0.16)	mitigating	0.60 [0.51; 0.71]	negative	0.52	1.4e-09	4.4e-09		

95%CI: 95% confidence interval, HR: hazard ratio.

Table S14: Mean absolute SHAP value of each model's feature, directionality of each feature's effect on PD risk, univariate association with Cox model, and comparison with the literature for the sensitivity analysis 5

Population	Feature/Predictor	mean |SHAP| (95% CI)	SHAP direction	HR [95% CI]	Cox direction	Cox (C-index)	Cox p-value	Adjusted Cox p-value	Literature	Reference
Farm manager	Acetaminophen	0.24 (0.06; 0.50)	promoting	1.06 [1.05; 1.07]	positive	0.58	2.5e-26	1.5e-25	conflicting results	[1,2]
Farm manager	Agricultural work companies (e.g., pesticide applications, harvest reaping)	9.7e-03 (6.3e-03; 0.01)	promoting	1.03 [1.00; 1.07]	positive	0.50	0.05	0.06	uncertain	[35]
Farm manager	Antidepressant	0.07 (0.03; 0.46)	promoting	1.02 [1.01; 1.02]	positive	0.54	3.6e-04	5.9e-04	risk factor	[6,7]
Farm manager	Anxiolytics	0.04 (4.8e-03; 0.10)	promoting	1.01 [1.00; 1.01]	uncertain	0.53	0.09	0.11	risk factor	[6,7]
Farm manager	Aspirin	9.4e-03 (3.1e-04; 0.10)	promoting	1.03 [1.02; 1.04]	positive	0.51	5.5e-08	1.1e-07	risk factor	[9,12]
Farm manager	Bipolar disorder	4.0e-03 (1.6e-03; 4.6e-03)	promoting	11.0 [8.29; 14.6]	positive	0.51	7.2e-62	7.4e-61	risk factor	[12,15]
Farm manager	Calcium channel blocker	7.8e-03 (8.4e-04; 0.10)	mitigating	0.96 [0.95; 0.97]	negative	0.51	9.6e-09	2.1e-08	protective factor	[9,12,15-18]
Farm manager	Cancer	4.4e-04 (1.1e-04; 2.9e-03)	mitigating	1.17 [1.00; 1.36]	uncertain	0.51	0.05	0.06	protective factor	[15]
Farm manager	Cow farming	0.16 (0.06; 1.01)	promoting	1.30 [1.14; 1.40]	positive	0.52	1.9e-12	5.6e-12	risk factor	[35]
Farm manager	Crop farming (e.g., wheat, corn, and industrial grower)	0.54 (0.15; 3.50)	promoting	1.46 [1.22; 1.81]	positive	0.56	2.8e-45	2.3e-44	risk factor	[35,36,38]
Farm manager	Dairy farming	0.16 (0.06; 0.76)	promoting	1.15 [1.06; 1.33]	positive	0.50	4.1e-04	6.2e-04	conflicting results	[35,39]
Farm manager	Depression	5.1e-03 (2.2e-03; 6.0e-03)	promoting	4.26 [3.23; 5.61]	positive	0.51	5.7e-25	2.9e-24	risk factor	[9,10,12,20,21]
Farm manager	Epilepsy	0.03 (9.6e-03; 0.05)	promoting	37.3 [31.9; 43.6]	positive	0.54	4.2e-208	8.6e-207	risk factor	[22,29,30]
Farm manager	Fruit arboriculture	0.03 (0.02; 0.04)	promoting	1.08 [1.04; 1.23]	positive	0.51	2.2e-04	3.8e-04	risk factor	[35,36]
Farm manager	Gardening, landscaping and reforestation companies	6.1e-03 (2.9e-03; 6.5e-03)	mitigating	1.00 [0.90; 1.32]	uncertain	0.50	0.50	0.53	protective factor	[35]
Farm manager	HMG CoA reductase inhibitors (statin)	0.22 (0.07; 0.74)	mitigating	0.96 [0.95; 0.97]	negative	0.51	9.9e-12	2.5e-11	protective factor	[9,12,15,16,23]
Farm manager	Hypercholesterolemia	0.55 (0.16; 0.91)	promoting	85.9 [76.8; 96.0]	positive	0.59	7.1e-264	2.9e-262	conflicting results	[24,31-34]
Farm manager	Hypertension	0.02 (4.9e-03; 0.18)	promoting	1.97 [1.62; 2.39]	positive	0.51	7.6e-12	2.1e-11	risk factor	[10,16,25]
Farm manager	Hypothyroidism	3.4e-04 (4.9e-05; 2.6e-03)	promoting	1.02 [0.84; 1.24]	uncertain	0.50	0.81	0.83	risk factor	[25,33]
Farm manager	Ibuprofen	0.03 (8.9e-03; 0.16)	mitigating	0.73 [0.68; 0.80]	negative	0.55	4.1e-14	1.4e-13	protective factor	[9,12,16]
Farm manager	Long-term psychiatric conditions	1.5e-03 (9.4e-04; 1.7e-03)	promoting	6.75 [4.58; 9.94]	positive	0.50	3.9e-22	1.8e-21		
Farm manager	Macrolides and lincosamides antibiotics	1.7e-04 (3.3e-05; 4.8e-04)	mitigating	0.67 [0.59; 0.75]	negative	0.53	1.1e-11	2.7e-11	protective factor	[28]
Farm manager	Median farm surface (hectares)	0.07 (1.6e-03; 0.59)	promoting	1.10 [0.92; 2.12]	uncertain	0.49	0.38	0.41		
Farm manager	Mixed cattle farming	0.05 (0.02; 0.09)	promoting	1.11 [1.03; 1.68]	positive	0.50	3.3e-03	4.8e-03	risk factor	[35-37]
Farm manager	Non-aspirin NSAIDs	0.02 (2.8e-03; 0.06)	mitigating	0.93 [0.91; 0.94]	negative	0.57	2.5e-16	1.0e-15	protective factor	[9,23,26]
Farm manager	Number of years of experience	0.18 (9.7e-03; 0.50)	mitigating	0.96 [0.92; 0.98]	negative	0.63	0.04	0.05		
Farm manager	Other antibacterials	6.4e-04 (2.1e-04; 3.1e-03)	mitigating	0.89 [0.81; 0.97]	negative	0.51	0.01	0.01		
Farm manager	Other beta-lactam antibacterials	4.1e-04 (1.1e-04; 1.7e-03)	mitigating	0.73 [0.66; 0.80]	negative	0.53	1.7e-10	3.9e-10		
Farm manager	Overall antibiotics	0.16 (0.04; 0.40)	mitigating	0.83 [0.81; 0.86]	negative	0.59	3.0e-35	2.1e-34		
Farm manager	Ovine and caprine farming	0.05 (0.03; 0.06)	mitigating	0.91 [0.82; 0.96]	negative	0.51	1.6e-07	3.1e-07	uncertain	[35,37]
Farm manager	Pig farming	7.4e-03 (5.3e-03; 8.0e-03)	promoting	1.08 [0.82; 1.43]	uncertain	0.50	0.94	0.94	conflicting results	[35-37]
Farm manager	Poultry and rabbit farming	0.01 (7.2e-03; 0.02)	mitigating	0.95 [0.81; 1.11]	uncertain	0.50	0.28	0.31	conflicting results	[35-37]
Farm manager	Stud farming	0.02 (0.01; 0.03)	mitigating	0.94 [0.88; 0.98]	negative	0.51	9.6e-05	1.8e-04	conflicting results	[35-37]
Farm manager	Sulfonamides	3.5e-03 (1e-03; 0.04)	mitigating	0.97 [0.96; 0.99]	negative	0.50	1.8e-04	3.2e-04	protective factor	[3]
Farm manager	Training, dressage, riding clubs	4.2e-03 (3.2e-03; 5.9e-03)	mitigating	0.98 [0.94; 1.00]	negative	0.50	0.04	0.05	protective factor	[35]
Farm manager	Truck farming, floriculture/flower-growing	0.04 (0.02; 0.05)	promoting	1.05 [1.02; 1.13]	positive	0.50	3.8e-04	6.0e-04	risk factor	[35]
Farm manager	Type 2 diabetes	9.0e-03 (8.4e-04; 0.06)	promoting	1.12 [0.95; 1.31]	uncertain	0.51	0.19	0.22	risk factor	[10,12,15,16,25]
Farm manager	Unspecified and mixed farming (e.g., polyculture, mixed farming, diversified farming)	0.19 (0.05; 1.19)	promoting	1.14 [1.06; 1.45]	positive	0.52	2.4e-15	6.0e-15	risk factor	[35]
Farm manager	Viticulture	0.21 (0.07; 1.22)	promoting	1.31 [1.16; 1.53]	positive	0.51	1.6e-12	5.1e-12	conflicting results	[35,36,40]
Farm manager	Work diversification	0.47 (0.02; 1.24)	mitigating	0.19 [0.16; 0.21]	negative	0.63	2.3e-110	3.1e-109		
Farm manager	Working as a solidarity contributor	0.02 (1.1e-03; 0.10)	mitigating	0.95 [0.87; 1.03]	uncertain	0.49	0.22	0.25		
Farmworker	Acetaminophen	0.09 (3.6e-03; 0.37)	promoting	1.00 [0.99; 1.02]	uncertain	0.53	0.69	0.69	conflicting results	[1,2]
Farmworker	Agricultural work companies (e.g., pesticide applications, harvest reaping)	0.02 (0.01; 0.02)	promoting	1.05 [1.01; 1.12]	positive	0.51	0.01	0.02		
Farmworker	Antidepressant	0.07 (0.03; 0.44)	promoting	1.05 [1.04; 1.06]	positive	0.57	6.7e-21	3.6e-20	risk factor	[6,7]
Farmworker	Anxiolytics	0.09 (0.02; 0.37)	promoting	1.04 [1.03; 1.05]	positive	0.58	8.9e-26	5.5e-25	risk factor	[6,7]
Farmworker	Aspirin	0.01 (8.6e-04; 0.08)	promoting	1.00 [0.99; 1.02]	uncertain	0.53	0.57	0.63	risk factor	[9,12]
Farmworker	Being a seasonal worker	0.36 (0.07; 0.82)	mitigating	0.28 [0.24; 0.33]	negative	0.62	3.4e-53	4.9e-52		

Farmworker	Bipolar disorder	4.2e-03 (1.9e-03; 5.1e-03)	promoting	19.5 [12.6; 30.0]	positive	0.51	3.1e-41	3.3e-40	risk factor	[12,15]
Farmworker	Calcium channel blocker	8.3e-03 (1.9e-04; 0.09)	mitigating	1.01 [0.99; 1.03]	uncertain	0.52	0.24	0.30	protective factor	[9,12,15-18]
Farmworker	Cancer	0.01 (2.7e-03; 0.08)	promoting	1.83 [1.44; 2.33]	positive	0.52	8.0e-07	2.3e-06	protective factor	[15]
Farmworker	Cerebrovascular disease	3.2e-03 (1.2e-03; 3.3e-03)	promoting	3.06 [1.98; 4.72]	positive	0.51	4.1e-07	1.3e-06	risk factor	[10,19]
Farmworker	Depression	2.6e-03 (5.5e-05; 2.9e-03)	promoting	6.37 [4.31; 9.40]	positive	0.51	1.4e-20	6.7e-20	risk factor	[9,10,12,20,21]
Farmworker	Epilepsy	0.02 (1.9e-03; 0.03)	promoting	84.2 [68.4; 104]	positive	0.56	6.4e-142	1.4e-140	risk factor	[22,29,30]
Farmworker	Fluoroquinolones	3.1e-03 (1.2e-03; 0.02)	promoting	0.97 [0.86; 1.10]	uncertain	0.49	0.66	0.69		
Farmworker	Gardener, park ranger	0.02 (0.01; 0.03)	mitigating	1.00 [0.95; 1.08]	uncertain	0.50	0.49	0.57		
Farmworker	Gardening and landscaping companies	0.03 (0.02; 0.03)	mitigating	0.97 [0.91; 1.03]	uncertain	0.50	0.19	0.26		
Farmworker	HMG CoA reductase inhibitors (statin)	0.13 (0.04; 0.88)	mitigating	1.01 [0.99; 1.02]	uncertain	0.54	0.41	0.49	protective factor	[9,12,15,16,23]
Farmworker	Hypercholesterolemia	0.27 (0.21; 0.34)	promoting	220 [187; 258]	positive	0.60	2.1e-180	9.0e-179	conflicting results	[24,31-34]
Farmworker	Hypertension	0.01 (5.7e-03; 0.02)	promoting	2.70 [1.88; 3.88]	positive	0.52	9.0e-08	3.0e-07	risk factor	[10,16,25]
Farmworker	Ibuprofen	0.05 (0.02; 0.19)	mitigating	0.88 [0.80; 0.97]	negative	0.51	7.1e-03	0.01	protective factor	[9,12,16]
Farmworker	Ischemic heart disease	2.4e-03 (5.9e-04; 4.6e-03)	promoting	2.04 [1.50; 2.78]	positive	0.51	4.8e-06	1.3e-05	risk factor	[15]
Farmworker	Large animal farming (e.g., cattle, ovine, pig)	3.3e-03 (2.6e-03; 4.1e-03)	promoting	1.07 [0.99; 1.21]	uncertain	0.50	0.07	0.11		
Farmworker	Long-term psychiatric conditions	9.0e-03 (4.2e-03; 9.5e-03)	promoting	13.6 [9.12; 20.2]	positive	0.51	8.2e-38	5.9e-37		
Farmworker	Macrolides and lincosamides antibiotics	4.4e-03 (7.0e-04; 0.02)	promoting	0.84 [0.72; 0.99]	negative	0.51	0.04	0.07	protective factor	[28]
Farmworker	Non-aspirin NSAIDs	0.09 (0.03; 0.19)	mitigating	0.98 [0.96; 1.00]	uncertain	0.50	0.11	0.16	protective factor	[9,23,26]
Farmworker	Number of years of experience	0.97 (0.03; 2.86)	mitigating	0.40 [0.24; 0.73]	negative	0.57	5.7e-04	8.9e-04		
Farmworker	Other beta-lactam antibacterials	1.4e-03 (3.5e-05; 8.3e-03)	promoting	0.92 [0.80; 1.05]	uncertain	0.50	0.22	0.29		
Farmworker	Overall antibiotics	0.13 (0.06; 0.38)	mitigating	0.95 [0.92; 0.98]	negative	0.49	4.4e-03	9.5e-03		
Farmworker	Penicillin antibiotics	6.2e-04 (1.1e-04; 2.6e-03)	promoting	0.90 [0.80; 1.01]	uncertain	0.50	0.07	0.11		
Farmworker	Percentage of part-time job	0.02 (1.4e-03; 0.17)	mitigating	0.73 [0.67; 0.86]	negative	0.50	6.9e-04	1.6e-03		
Farmworker	Shellfish farming (e.g., oyster farming, scallop aquaculture)	0.01 (7.9e-03; 0.02)	mitigating	0.95 [0.91; 1.02]	uncertain	0.50	0.08	0.12		
Farmworker	Small animal farming (e.g., poultry, rabbit, fish, bee)	0.01 (7.2e-03; 0.01)	mitigating	0.96 [0.92; 1.04]	uncertain	0.50	0.08	0.12		
Farmworker	Stationary sawmill (e.g., edging, trimming, decking, debarking)	2.8e-03 (2.5e-03; 3.5e-03)	mitigating	1.01 [0.88; 1.15]	uncertain	0.51	0.29	0.36		
Farmworker	Sylviculture/forestry (e.g., thinning, pruning)	1.1e-03 (7.1e-04; 1.2e-03)	mitigating	1.00 [0.75; 1.35]	uncertain	0.50	0.67	0.69		
Farmworker	Training, dressage, stud farm	6.7e-03 (5.2e-03; 8.0e-03)	mitigating	1.00 [0.90; 1.08]	uncertain	0.50	0.17	0.24	conflicting results	[35-37]
Farmworker	Type 2 diabetes	0.04 (8.7e-03; 0.22)	promoting	2.08 [1.67; 2.59]	positive	0.53	4.1e-11	1.6e-10	risk factor	[10,12,15,16,25]
Farmworker	Unspecified farming (e.g., mixed farming, cereal farming, potato farming, pasture)	0.08 (0.03; 0.45)	promoting	1.11 [1.06; 1.21]	positive	0.52	1.7e-05	4.3e-05		
Farmworker	Unspecified specialized crop farming (e.g., soy farming, fruit arboriculture, truck farming)	0.11 (0.04; 0.43)	promoting	1.22 [1.13; 1.45]	positive	0.54	1.1e-11	4.7e-11		
Farmworker	Vinification	0.01 (9.2e-03; 0.02)	promoting	1.04 [1.02; 1.14]	positive	0.50	0.04	0.07		
Farmworker	Viticulture	0.41 (0.14; 1.98)	promoting	1.37 [1.19; 1.68]	positive	0.63	4.0e-38	3.4e-37	conflicting results	[35,36,40]
Farmworker	Wood production (e.g., lopping)	5.6e-03 (4.3e-03; 6.1e-03)	mitigating	1.00 [0.88; 1.32]	uncertain	0.50	0.67	0.69	uncertain	[38]
Farmworker	Working in a company with between 50 and 149 workers	2.0e-03 (2.9e-04; 0.01)	promoting	1.34 [0.98; 1.84]	uncertain	0.50	0.07	0.11		
Farmworker	Working in a company with over 299 workers	2.9e-04 (8.7e-05; 1.4e-03)	promoting	1.74 [1.29; 2.36]	positive	0.51	3.3e-04	7.9e-04		
Farmworker	Working in a company with fewer than 20 workers	0.02 (6.1e-04; 0.11)	mitigating	0.58 [0.50; 0.69]	negative	0.53	1.3e-10	4.7e-10		

95%CI: 95% confidence interval, HR: hazard ratio.

Table S16: Mean absolute SHAP value of each model's feature, directionality of each feature's effect on PD risk, univariate association with Cox model, and comparison with the literature for the sensitivity analysis 6

Population	Feature/Predictor	mean |SHAP| (95% CI)	SHAP direction	HR [95% CI]	Cox direction	Cox (C-index)	Cox p-value	Adjusted Cox p-value	Literature	Reference
Farm manager	Age	0.38 (0.03; 0.85)	promoting	14.3 [10.1; 20.4]	positive	0.63	1.3e-49	2.6e-49	risk factor	[4,5]
Farm manager	Being a male	0.05 (4.1e-03; 0.13)	promoting	1.10 [1.02; 1.18]	positive	0.63	0.01	0.01	risk factor	[14]
Farmworker	Age	0.29 (0.04; 0.62)	promoting	19.7 [9.40; 41.3]	positive	0.63	3.0e-15	5.9e-15	risk factor	[4,5]
Farmworker	Being a male	0.03 (0.02; 0.04)	promoting	1.01 [0.88; 1.16]	uncertain	0.63	0.98	0.98	risk factor	[14]

95%CI: 95% confidence interval, HR: hazard ratio.

RECORD Statement Checklist

	Item No.	STROBE items	Location in manuscript where items are reported	RECORD items	Location in manuscript where items are reported
**Title and abstract**					
	1	(a) Indicate the study's design with a commonly used term in the title or the abstract (b) Provide in the abstract an informative and balanced summary of what was done and what was found		RECORD 1.1: The type of data used should be specified in the title or abstract. When possible, the name of the databases used should be included.<br><br>RECORD 1.2: If applicable, the geographic region and timeframe within which the study took place should be reported in the title or abstract.<br><br>RECORD 1.3: If linkage between databases was conducted for the study, this should be clearly stated in the title or abstract.	1, 2<br><br>1, 2<br><br>1, 2
**Introduction**					
Background rationale	2	Explain the scientific background and rationale for the investigation being reported			3, 4
Objectives	3	State specific objectives, including any prespecified hypotheses			4
**Methods**					
Study Design	4	Present key elements of study design early in the paper			4-6
Setting	5	Describe the setting, locations, and relevant dates, including periods of recruitment, exposure, follow-up, and data collection			4-6
Participants	6	*(a) Cohort study* - Give the eligibility criteria, and the sources and methods of selection of participants. Describe methods of follow-up<br>*Case-control study* - Give the eligibility criteria, and the sources and methods of case ascertainment and control selection. Give the rationale for the choice of cases and controls<br>*Cross-sectional study* - Give the eligibility criteria, and the sources and methods of selection of participants<br><br>*(b) Cohort study* - For matched studies, give matching criteria and number of exposed and unexposed<br>*Case-control study* - For matched studies, give matching criteria and the number of controls per case		RECORD 6.1: The methods of study population selection (such as codes or algorithms used to identify subjects) should be listed in detail. If this is not possible, an explanation should be provided.<br><br>RECORD 6.2: Any validation studies of the codes or algorithms used to select the population should be referenced. If validation was conducted for this study and not published elsewhere, detailed methods and results should be provided.<br><br>RECORD 6.3: If the study involved linkage of databases, consider use of a flow diagram or other graphical display to demonstrate the data linkage process, including the number of individuals with linked data at each stage.	4-7<br><br>4-7<br><br>DNA
Variables	7	Clearly define all outcomes, exposures, predictors, potential confounders, and effect modifiers. Give diagnostic criteria, if applicable.		RECORD 7.1: A complete list of codes and algorithms used to classify exposures, outcomes, confounders, and effect modifiers should be provided. If these cannot be reported, an explanation should be provided.	5-7, Suppl. Mat.
Data sources/ measurement	8	For each variable of interest, give sources of data and details of methods of assessment (measurement).<br>Describe comparability of assessment methods if there is more than one group			
Bias	9	Describe any efforts to address potential sources of bias			5-9
Study size	10	Explain how the study size was arrived at			4-7, Suppl. Mat.
Quantitative variables	11	Explain how quantitative variables were handled in the analyses. If applicable, describe which groupings were chosen, and why			6-7, Suppl. Mat.
Statistical methods	12	(a) Describe all statistical methods, including those used to control for confounding<br>(b) Describe any methods used to examine subgroups and interactions<br>(c) Explain how missing data were addressed<br>(d) *Cohort study* - If applicable, explain how loss to follow-up was addressed<br>*Case-control study* - If applicable, explain how matching of cases and controls was addressed			7-9, Suppl. Mat.

		Cross-sectional study - If applicable, describe analytical methods taking account of sampling strategy<br>(e) Describe any sensitivity analyses			
Data access and cleaning methods		..		RECORD 12.1: Authors should describe the extent to which the investigators had access to the database population used to create the study population.<br><br>RECORD 12.2: Authors should provide information on the data cleaning methods used in the study.	4, 5<br><br>4, 5
Linkage		..		RECORD 12.3: State whether the study included person-level, institutional-level, or other data linkage across two or more databases. The methods of linkage and methods of linkage quality evaluation should be provided.	4, 5
**Results**					
Participants	13	(a) Report the numbers of individuals at each stage of the study (*e.g.*, numbers potentially eligible, examined for eligibility, confirmed eligible, included in the study, completing follow-up, and analysed)<br>(b) Give reasons for non-participation at each stage.<br>(c) Consider use of a flow diagram		RECORD 13.1: Describe in detail the selection of the persons included in the study (*i.e.,* study population selection) including filtering based on data quality, data availability and linkage. The selection of included persons can be described in the text and/or by means of the study flow diagram.	9, 10, Suppl. Mat.
Descriptive data	14	(a) Give characteristics of study participants (*e.g.*, demographic, clinical, social) and information on exposures and potential confounders<br>(b) Indicate the number of participants with missing data for each variable of interest<br>(c) *Cohort study* - summarise follow-up time (*e.g.*, average and total amount)			9, 10, Suppl. Mat.
Outcome data	15	*Cohort study* - Report numbers of outcome events or summary measures over time<br>*Case-control study* - Report numbers in each exposure category, or summary measures of exposure<br>*Cross-sectional study* - Report numbers of outcome events or summary measures			11-14, Suppl. Mat.
Main results	16	(a) Give unadjusted estimates and, if applicable, confounder-adjusted estimates and their precision (e.g., 95% confidence interval). Make clear which confounders were adjusted for and why they were included<br>(b) Report category boundaries when continuous variables were categorized<br>(c) If relevant, consider translating estimates of relative risk into absolute risk for a meaningful time period			11-14, Suppl. Mat.
Other analyses	17	Report other analyses done—e.g., analyses of subgroups and interactions, and sensitivity analyses			12-14, Suppl. Mat.
**Discussion**					
Key results	18	Summarise key results with reference to study objectives			19
Limitations	19	Discuss limitations of the study, taking into account sources of potential bias or imprecision. Discuss both direction and magnitude of any potential bias		RECORD 19.1: Discuss the implications of using data that were not created or collected to answer the specific research question(s). Include discussion of misclassification bias, unmeasured confounding, missing data, and changing eligibility over time, as they pertain to the study being reported.	23, 24
Interpretation	20	Give a cautious overall interpretation of results considering objectives, limitations, multiplicity of analyses, results from similar studies, and other relevant evidence			19-25
Generalisability	21	Discuss the generalisability (external validity) of the study results			24, 25

**Other Information**					
Funding	22	Give the source of funding and the role of the funders for the present study and, if applicable, for the original study on which the present article is based			27
Accessibility of protocol, raw data, and programming code		..		RECORD 22.1: Authors should provide information on how to access any supplemental information such as the study protocol, raw data, or programming code.	27

*Reference: Benchimol EI, Smeeth L, Guttmann A, Harron K, Moher D, Petersen I, Sørensen HT, von Elm E, Langan SM, the RECORD Working Committee. The REporting of studies Conducted using Observational Routinely-collected health Data (RECORD) Statement. *PLoS Medicine* 2015; in press.

DNA: does not applied, Suppl. Mat.: supplementary materials.

References

[1] Bohler S, Liu X, Krauskopf J, et al. Acetaminophen Overdose as a Potential Risk Factor for Parkinson's Disease. *Clin Transl Sci* 2019; 12(6): 609–616.

[2] Gagne JJ and Power MC. Anti-inflammatory drugs and risk of Parkinson disease: a meta-analysis. *Neurology* 2010; 74(12): 995–1002.

[3] Courtois É, Nguyen TTH, Fournier A, et al. Identifying Protective Drugs for Parkinson's Disease in Health-Care Databases Using Machine Learning. *Mov Disord* 2022; 37(12): 2376–2385.

[4] Ben-Shlomo Y, Darweesh S, Llibre-Guerra J, et al. The epidemiology of Parkinson's disease. *Lancet* 2024; 403(10423): 283–292.

[5] Sturm ET, Castro C, Mendez-Colmenares A, et al. Risk Factors for Brain Health in Agricultural Work: A Systematic Review. *Int J Environ Res Public Health* 2022; 19(6): 3373.

[6] Beydoun HA, Saquib N, Wallace RB, et al. Psychotropic medication use and Parkinson's disease risk amongst older women. *Ann Clin Transl Neurol* 2022; 9(8): 1163–1176.

[7] Dick FD, De Palma G, Ahmadi A, et al. Environmental risk factors for Parkinson's disease and parkinsonism: the Geoparkinson study. *Occup Environ Med* 2007; 64(10): 666–672.

[8] Camacho-Soto A, Faust I, Racette BA, et al. Herpesvirus Infections and Risk of Parkinson's Disease. *Neurodegener Dis* 2020; 20(2–3): 97–103.

[9] Delamarre A and Meissner WG. Epidemiology, environmental risk factors and genetics of Parkinson's disease. *Presse Med* 2017;46 (2 Pt 1): 175–181.

[10] Martino R, Candundo H, Lieshout PV, et al. Onset and progression factors in Parkinson's disease: A systematic review. *Neurotoxicology* 2017; 61: 132–141.

[11] Lin CH, Lin JW, Liu YC, et al. Risk of Parkinson's disease following anxiety disorders: a nationwide population-based cohort study. *Eur J Neurol* 2015; 22(9): 1280–1287.

[12] Chen Y, Sun X, Lin Y, et al. Non-Genetic Risk Factors for Parkinson's Disease: An Overview of 46 Systematic Reviews. *J Parkinsons Dis* 2021; 11(3): 919–935.

[13] Cheng CM, Wu YH, Tsai SJ, et al. Risk of developing Parkinson's disease among patients with asthma: a nationwide longitudinal study. *Allergy* 2015; 70(12): 1605–1612.

[14] Zhang J, Zhou W, Yu H, et al. Prediction of Parkinson's Disease Using Machine Learning Methods. *Biomolecules*. 2023; 13(12): 1761.

[15] Grotewold N and Albin RL. Update: Protective and risk factors for Parkinson disease. *Parkinsonism Relat Disord* 2024; 125: 107026.

[16] Ascherio A and Schwarzschild MA. The epidemiology of Parkinson's disease: risk factors and prevention. *Lancet Neurol* 2016; 15(12): 1257–1272.

[17] Reichmann H, Csoti I, Koschel J, et al. Life style and Parkinson's disease. *J Neural Transm (Vienna)* 2022; 129(9): 1235–1245.

[18] Lin J, Pang D, Li C, et al. Calcium channel blockers and Parkinson's disease: a systematic review and meta-analysis. *Ther Adv Neurol Disord* 2024; 17: 17562864241252713.

[19] Kummer BR, Diaz I, Wu X, et al. Associations between cerebrovascular risk factors and parkinson disease. *Ann Neurol* 2019; 86(4): 572–581.

[20] Alonso A, Rodríguez LA, Logroscino G, et al. Use of antidepressants and the risk of Parkinson's disease: a prospective study. *J Neurol Neurosurg Psychiatry* 2009; 80(6): 671–674.

[21] Gustafsson H, Nordström A and Nordström P. Depression and subsequent risk of Parkinson disease: A nationwide cohort study. *Neurology* 2015; 84(24): 2422–2429.

[22] Hwang YS, Kang MG, Yeom SW, et al. Increasing incidence of Parkinson's disease in patients with epilepsy: A Nationwide cohort study. *J Neurol Sci* 2024; 458: 122891.

[23] Mentis AA, Dardiotis E, Efthymiou V, et al. Non-genetic risk and protective factors and biomarkers for neurological disorders: a meta-umbrella systematic review of umbrella reviews. *BMC Med* 2021; 19(1): 6.

[24] Miyake Y, Tanaka K, Fukushima W, et al. Case-control study of risk of Parkinson's disease in relation to hypertension, hypercholesterolemia, and diabetes in Japan. *J Neurol Sci* 2010; 293(1–2): 82–86.

[25] Gialluisi A, De Bartolo MI, Costanzo S, et al. Risk and protective factors in Parkinson's disease: a simultaneous and prospective study with classical statistical and novel machine learning models. *J Neurol* 2023; 270(9): 4487–4497.

[26] Huang Y, Chen Q, Wang Z, et al. Risk factors associated with age at onset of Parkinson's disease in the UK Biobank. *NPJ Parkinsons Dis* 2024; 10(1): 3.

[27] Belvisi D, Pellicciari R, Fabbrini A, et al. Risk factors of Parkinson disease: Simultaneous assessment, interactions, and etiologic subtypes. *Neurology* 2020; 95(18): e2500–e2508.

[28] Pylkkö E, Courtois É, Paakinaho A, et al. Identifying Drugs Associated With Parkinson's Disease Risk Using Machine Learning. *Basic Clin Pharmacol Toxicol* 2026; 138(3): e70192.

[29] Reynoso A, Torricelli R, Jacobs BM, et al. Gene-Environment Interactions for Parkinson's Disease. *Ann Neurol* 2024; 95(4): 677–687.

[30] Heilbron K, Noyce AJ, Fontanillas P, et al. The Parkinson's phenome-traits associated with Parkinson's disease in a broadly phenotyped cohort. *NPJ Parkinsons Dis*. 2019; 5: 4.

[31] Johri M, Morrill V, Okine DN, et al. Midlife Vascular Risk Factors, Parkinson Disease, and Parkinson Disease-Dementia: The ARIC Study. *Neurology* 2025; 105(6): e213931.

[32] Paul R, Choudhury A, Chandra Boruah D, et al. Hypercholesterolemia causes psychomotor abnormalities in mice and alterations in cortico-striatal biogenic amine neurotransmitters: Relevance to Parkinson's disease. *Neurochem Int* 2017; 108: 15–26.

[33] Mohammadi S, Dolatshahi M and Rahmani F. Shedding light on thyroid hormone disorders and Parkinson disease pathology: mechanisms and risk factors. *J Endocrinol Invest* 2021; 44(1): 1–13.

[34] Alrouji M, Al-Kuraishy HM, Al-Mahammadawy AAA, et al. The potential role of cholesterol in Parkinson's disease neuropathology: perpetrator or victim. *Neurol Sci* 2023; 44(11): 3781–3794.

[35] Petit P, Berger F, Bonneterre V, et al. Investigating Parkinson's disease risk across farming activities using data mining and large-scale administrative health data. *NPJ Parkinsons Dis* 2025; 11(1): 13.

[36] Pouchieu C, Piel C, Carles C, et al. Pesticide use in agriculture and Parkinson's disease in the AGRICAN cohort study. *Int J Epidemiol* 2018; 47(1): 299–310.

[37] Harris MA, Tsui JK, Marion SA, et al. Association of Parkinson's disease with infections and occupational exposure to possible vectors. *Mov Disord* 2012; 27(9): 1111–1117.

[38] Semchuk KM, Love EJ and Lee RG. Parkinson's disease and exposure to agricultural work and pesticide chemicals. *Neurology* 1992; 42(7): 1328–1335.

[39] Firestone JA, Smith-Weller T, Franklin G, et al. Pesticides and risk of Parkinson disease: a population-based case-control study. *Arch Neurol* 2005; 62(1): 91–95.

[40] Moisan F, Spinosi J, Delabre L, et al. Association of Parkinson's Disease and Its Subtypes with Agricultural Pesticide Exposures in Men: A Case-Control Study in France. *Environ Health Perspect* 2015; 123(11): 1123–1129.
